# CRISP: a correlation-filtered recursive feature elimination and integration of SMOTE pipeline for gait-based Parkinson’s disease screening

**DOI:** 10.3389/fncom.2025.1660963

**Published:** 2025-10-10

**Authors:** Namra Afzal, Javaid Iqbal, Asim Waris, Muhammad Jawad Khan, Fawwaz Hazzazi, Hasnain Ali, Muhammad Adeel Ijaz, Syed Omer Gilani

**Affiliations:** ^1^Department of Biomedical Engineering and Sciences, School of Mechanical and Manufacturing Engineering, National University of Science and Technology (NUST), Islamabad, Pakistan; ^2^Department of Electrical Engineering, College of Engineering, Prince Sattam bin Abdulaziz University, Al-Kharj, Saudi Arabia; ^3^Department of Electrical, Computer, and Biomedical Engineering. College of Engineering, Abu Dhabi University, Abu Dhabi, United Arab Emirates

**Keywords:** Parkinson’s disease, gait analysis, correlation-filtered feature pruning, vertical ground-reaction force, subject-wise accuracy, XGBoost

## Abstract

**Introduction:**

Parkinson’s disease (PD) is the fastest-growing neurodegenerative disorder, with subtle gait changes such as reduced vertical ground-reaction forces (VGRF) often preceding motor symptoms. These gait abnormalities, measurable via wearable VGRF sensors, offer a non-invasive means for early PD detection. However, current computational approaches often suffer from redundant features and class imbalance, limiting both accuracy and generalizability.

**Methods:**

We propose CRISP (Correlation-filtered Recursive Feature Elimination and Integration of SMOTE Pipeline for Gait-Based Parkinson’s Disease Screening), a lightweight multistage framework that sequentially applies correlation-based feature pruning, recursive feature elimination (RFE), and Synthetic Minority Oversampling Technique (SMOTE) based class balancing. To ensure clinically meaningful evaluation, a novel subject-wise protocol was also introduced that assigns one prediction per individual enhancing patient-level variability capture and better aligning with diagnostic workflows. Using 306 VGRF recordings (93 PD, 76 controls), five classifiers, i.e., ﻿k-Nearest Neighbours (KNN), Decision Tree (DT), Random Forest (RF), Gradient boosting (GB), and Extreme Gradient Boosting (XGBoost) were evaluated for both binary PD detection and multiclass severity grading.

**Results:**

CRISP consistently improved performance across all models under 5-fold cross-validation. XGBoost achieved the highest performance, increasing subject-wise PD detection accuracy from 96.1 ± 0.8% to 98.3 ± 0.8%, and severity grading accuracy from 96.2 ± 0.7% to 99.3 ± 0.5%.

**Conclusion:**

CRISP is the first VGRF-based pipeline to combine correlation-filtered feature pruning, recursive feature elimination, and SMOTE to enhance PD detection performance, while also introducing a subject-wise evaluation protocol that captures patient-level variability for truly personalized diagnostics. These twin novelties deliver clinically significant gains and lay the foundation for real-time, on-device PD detection and severity monitoring.

## 1 Introduction

Parkinson’s disease (PD) is a neurodegenerative disorder characterized by the progressive loss of dopamine-producing neurons in the brain (Ramesh and Arachchige, 2023), leading to motor impairments such as tremors, bradykinesia, and rigidity, which severely affect a patient’s daily functioning (Kumar et al., 2021). Early diagnosis and intervention are crucial in managing PD, as current treatments can alleviate symptoms but cannot reverse the underlying neurodegeneration (Bloem et al., 2021). However, conventional diagnostic methods, such as neurological exams and brain imaging, often fall short in detecting the disease in its early, subtle stages. This limitation highlights the urgent need for non-invasive, early-stage diagnostic tools to detect PD at its initial, asymptomatic phase (Brindha et al., 2022).

Gait abnormalities, such as reduced stride length, slower walking speed, and irregular cadence, are often among the earliest signs of PD, appearing before more obvious symptoms like tremors (Baker, 2018; Barberà et al., 2024). Wearable sensors, including Inertial Measurement Units (IMUs) and accelerometers, have shown significant promise for continuously monitoring gait in real-world settings. These sensors capture real-time, three-dimensional motion data, providing a non-invasive and scalable method for detecting subtle gait changes indicative of PD (Salchow-Hömmen et al., 2022; Hemmerling et al., 2025). The use of these sensors offers a potential solution for early-stage, real-time PD detection, enabling more timely and accurate diagnoses. To further this goal, the PhysioNet gait dataset has become an essential resource in PD research. This dataset includes comprehensive recordings of ﻿vertical ground-reaction forces (VGRF) (Alam et al., 2017; Etoom et al., 2024) and IMU data from both PD patients and healthy controls, offering a valuable foundation for training machine learning (ML) models (Alkhatib et al., 2020; Chudzik et al., 2024). The PhysioNet dataset enables researchers to examine the relationship between gait abnormalities and PD, supporting the development of diagnostic models that can accurately identify subtle gait changes associated with the disease (Baker, 2018; Veeraragavan et al., 2020; Bloem et al., 2021; Salchow-Hömmen et al., 2022; Etoom et al., 2024).

ML models have become a crucial tool in analyzing gait data for PD detection. These models can process large volumes of data collected from wearable sensors, detecting subtle patterns and abnormalities in gait that may not be apparent to human observers (Rana et al., 2022). Popular approaches such as ﻿k-Nearest Neighbors (KNN), Decision Tree (DT) and other ensemble or distance-based classifiers have demonstrated promising results in distinguishing PD from healthy gait patterns (Roy et al., 2024). Several studies have further shown that models capable of capturing spatial and temporal dependencies in gait signals can enhance classification accuracy reporting high performance levels (Liu et al., 2021). These models offer a scalable solution for analyzing gait data, providing high diagnostic precision and enabling earlier detection of PD (Shalin et al., 2021; Choi et al., 2024; Srinivasarao et al., 2024; Navita Mittal et al., 2025). Related studies have also explored alternative modalities such as voice analysis (Ghaheri et al., 2024) and neuromorphic systems (Siddique et al., 2023) for PD screening. The effectiveness of these ML models highlights their potential for a real-time and reliable benchmark for PD diagnosis (Joshi et al., 2017; Zeng et al., 2019; ﻿Abdullah et al., 2024).

Despite these advances, several key challenges remain. First, gait-based datasets often begin with a high-dimensional feature space yet few studies systematically prune redundant or irrelevant features before model training. Second, class imbalance in PD versus control recordings can bias classifiers such as ﻿k-Nearest Neighbors (KNN), Decision Tree (DT), Random Forest (RF), Gradient Boosting (GB), and Extreme Gradient Boosting (XGBoost) toward the majority class, thus reducing generalization. Third, most studies leveraging datasets like PhysioNet report only aggregated accuracy across all gait cycles, masking subject-level variability critical for clinical interpretation and personalized diagnostics (Horst et al., 2019; Maachi et al., 2020; Dong et al., 2023).

To address these gaps, CRISP a unified pipeline, was proposed that (i) filters out highly correlated features, (ii) applies recursive feature elimination (RFE) to isolate the most informative metrics, and (iii) balances each training fold with SMOTE (Synthetic Minority Oversampling Technique) to counter class imbalance. In tandem, we adopt a subject-wise evaluation protocol that evaluates each patient separately ensuring one prediction per individual and thus faithfully captures patient-level performance.

CRISP was evaluated on 306 ﻿VGRF recordings (93 PD, 76 control subjects) using five mainstream classifiers (KNN, DT, RF, GB, ﻿XGBoost) across two tasks: binary PD detection and multiclass severity grading (Hoehn & Yahr scale). To our knowledge, this is the first study to integrate correlation pruning, RFE, and SMOTE into a single, efficient pipeline for VGRF-based PD screening while also rigorously evaluating patient-level performance. Our results show that CRISP consistently improves performance across all models. For the best-performing classifier, XGBoost, subject-wise accuracy improves from 96.1 ± 0.8% to 98.3 ± 0.8% for PD detection, and from 96.2 ± 0.7% to 99.3 ± 0.5% for severity grading. These results highlight the critical importance of both careful feature curation and subject-wise evaluation for personalized diagnostics and pave the way for real-time, on-device PD screening and severity monitoring.

This study makes the following key contributions:

1. We propose CRISP (Correlation-filtered Recursive feature elimination and Integration of SMOTE Pipeline), a lightweight, modular framework that sequentially (i) prunes redundant features through correlation filtering, (ii) selects the most informative subset using ﻿RFE and (iii) mitigates class imbalance via SMOTE. This is the first study to combine all three components in a unified pipeline for ﻿VGRF-based PD screening delivering substantial performance gains across five classifiers (KNN, DT, RF, GB, ﻿XGBoost).
2. We introduce a rigorous subject-wise cross-validation scheme that issues a single prediction per individual, in contrast to conventional cycle-level aggregation. This approach captures patient-level variability essential for real-world, personalized diagnostics and provides a more clinically interpretable performance estimate.
3. CRISP consistently improves diagnostic accuracy across both binary PD detection and multiclass severity grading (Hoehn & Yahr scale). For the best-performing model (XGBoost), subject-wise accuracy improves from 96.1 ± 0.8% to 98.3 ± 0.8% for binary classification, and from 96.2 ± 0.7% to 99.3 ± 0.5% for severity grading showcasing the robustness and generalizability of our pipeline.
4. When paired with efficient learners such as XGBoost, CRISP supports rapid inference with minimal computational overhead, making it suitable for real-time, on-device deployment. This positions our method as a scalable solution for continuous, non-invasive monitoring in both clinical and home settings.

This study provides a promising approach for early, non-invasive PD screening by combining advanced feature curation and personalized evaluation, demonstrating that lightweight pipelines like CRISP can deliver both high accuracy and real-time performance. Our results pave the way for scalable, on-device diagnostic tools that could potentially bring personalized gait-based monitoring out of the lab and into everyday clinical and at-home use.

## 2 Materials and methods

**Figure 1:**
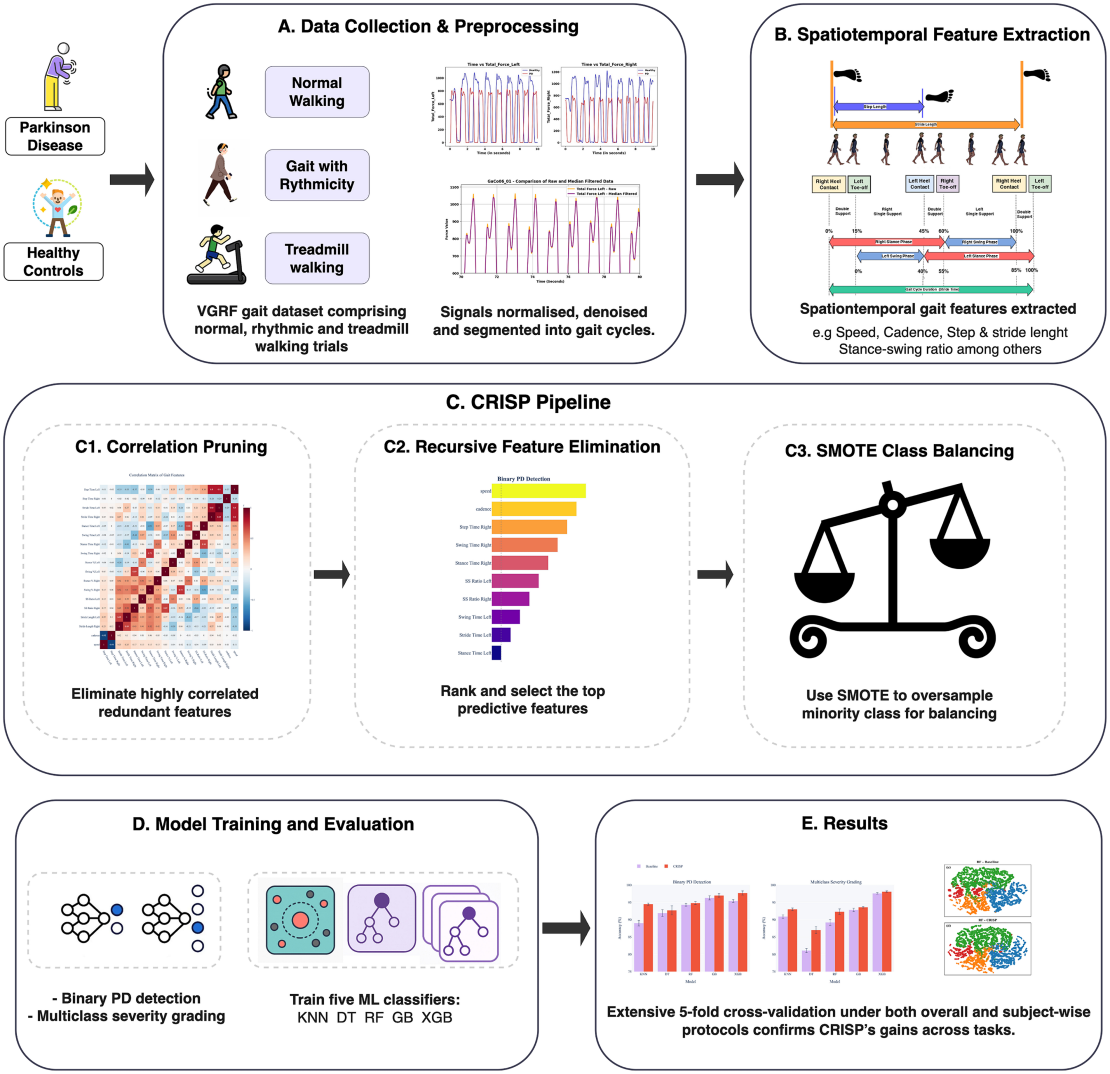
Correlation-filtered Recursive feature elimination and Integration of SMOTE Pipeline overview for gait-based Parkinson’s detection. **(A)** Data collection and preprocessing. **(B)** Spatiotemporal feature extraction. **(C)** Consecutive stages. (c1) Correlation pruning (c2) Recursive feature elimination (c3) SMOTE class balancing synthetically oversamples minority classes inside every training fold to balance the dataset. **(D)** Model training and evaluation. **(E)** Results.

This study used The CRISP pipeline for gait-based PD detection (Figure 1) includes three modules: correlation-based feature pruning, ﻿RFE, and SMOTE-based class balancing, which together reduce dimensionality, balance classes, and improve generalization. Model development and evaluation (Section 2.5) employed five classifiers (KNN, DT, RF, GB, ﻿XGBoost) under a subject-wise protocol. Performance was measured for binary PD detection and multiclass Hoehn–Yahr grading using Accuracy, Precision, Recall, Specificity, F1-score, and ROC-AUC.

### 2.1 Dataset

﻿VGRF signals from an open-access gait database comprising 93 PD patients and 76 healthy controls (HC) were obtained from the PhysioNet Gait in Neurodegenerative Disease database (Goldberger et al., 2000), which aggregates data from three clinical studies (Frenkel-Toledo et al., 2005; Yogev et al., 2005; Hausdorff, 2008). These recordings capture multiple walking conditions using instrumented footwear equipped with 16 force-sensitive resistors, eight per shoe sole, sampling at 100 Hz to measure plantar VGRF over time (Zeng et al., 2019). Further details of Dataset are in section S6 of cohort demographics in Supplementary file.

### 2.2 Preprocessing and gait-event detection

Raw VGRF signals were recorded using 16 force-sensitive sensors embedded in each shoe sole and normalized to the [0,1] range via min–max scaling. To reduce artefacts, the first 20 s and last 10 s of each 60-s walk were excluded. High-frequency noise was suppressed using a 10-point median filter (kernel size 9), preserving waveform integrity (Zeng et al., 2019; Wang et al., 2024). Heel-strike (HS) and toe-off (TO) were detected using a 20% threshold of peak vertical force, with HS defined by upward crossing and TO by the subsequent downward crossing (Alkhatib et al., 2020). A gait cycle comprised two successive HS events, with features computed per cycle. Supplementary Figure S12 compares VGRF profiles of PD and healthy subjects, while Supplementary Figure S13 illustrates the smoothing effect of median filtering.

### 2.3 Spatiotemporal feature extraction

Heel-strike and toe-off events (Section 2.2) were used to segment gait cycles, from which eight spatiotemporal features were extracted: stride time, stance time, swing time, their percentages, cadence, step length, and stride length (Supplementary Table S14; Supplementary Figure S14). For each participant, features were computed per cycle and summarized as mean and standard deviation across valid strides. Group-level statistics (Supplementary Table S15) show PD subjects with longer stride and swing times, higher stance percentage, and reduced cadence, patterns consistent with bradykinesia and postural instability. These distinct features were used as predictors in the supervised learning models. In order to avoid any potential label leakage, both RFE and mutual information-based feature selection were applied strictly within each training fold during the cross-validation process, ensuring that no information from the test sets influenced the feature selection process.

### 2.4 CRISP overview

CRISP consists of three stages: (i) Pearson correlation analysis to remove highly collinear features (|r| ≥ 0.80), (ii) ﻿RFE with a 100-tree random forest to select the 10 most predictive features, and (iii) SMOTE to balance classes within each training fold. The curated features were then used to train five classifiers (KNN, DT, RF, GB, XGBoost), evaluated under both cycle-level and subject-wise cross-validation. Outputs included binary PD detection and multiclass Hoehn–Yahr grading, assessed using Accuracy, Precision, Recall, Specificity, F1-score, and ROC-AUC.

#### 2.4.1 Correlation pruning

**Figure 2:**
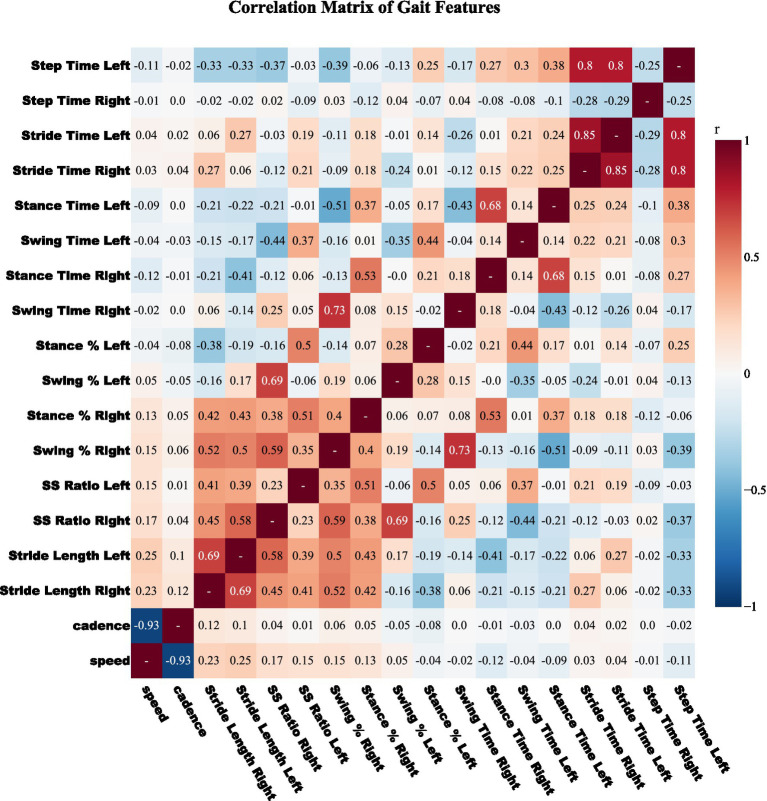
Pearson correlation matrix among raw gait features. Pairwise correlation heatmap of gait features colored on a diverging red-blue scale (positive to negative). Features with |r| ≥ 0.80 are considered highly correlated, and the one with lower mutual information relative to the label is discarded. This pruning step reduces redundancy prior to feature selection in CRISP.

To reduce multicollinearity, we computed pairwise Pearson correlations for all gait features (Figure 2). Feature names were standardized, coefficients rounded to two decimals, and a threshold of |r| ≥ 0.80 applied to flag strongly correlated pairs. This cutoff, commonly used in biomedical predictive modeling (Kuhn and Johnson, 2013; Connolly et al., 2016), avoids excessive feature loss. For each correlated pair, mutual information (MI) with respect to the diagnostic label (PD status or Hoehn–Yahr grade) was calculated, and the feature with lower MI was discarded. This pruning removed six features, reducing dimensionality to 24 while retaining those most informative. All correlation and MI calculations were restricted to training data to avoid information leakage.

#### 2.4.2 Feature selection with RFE (recursive feature elimination)

**Figure 3:**
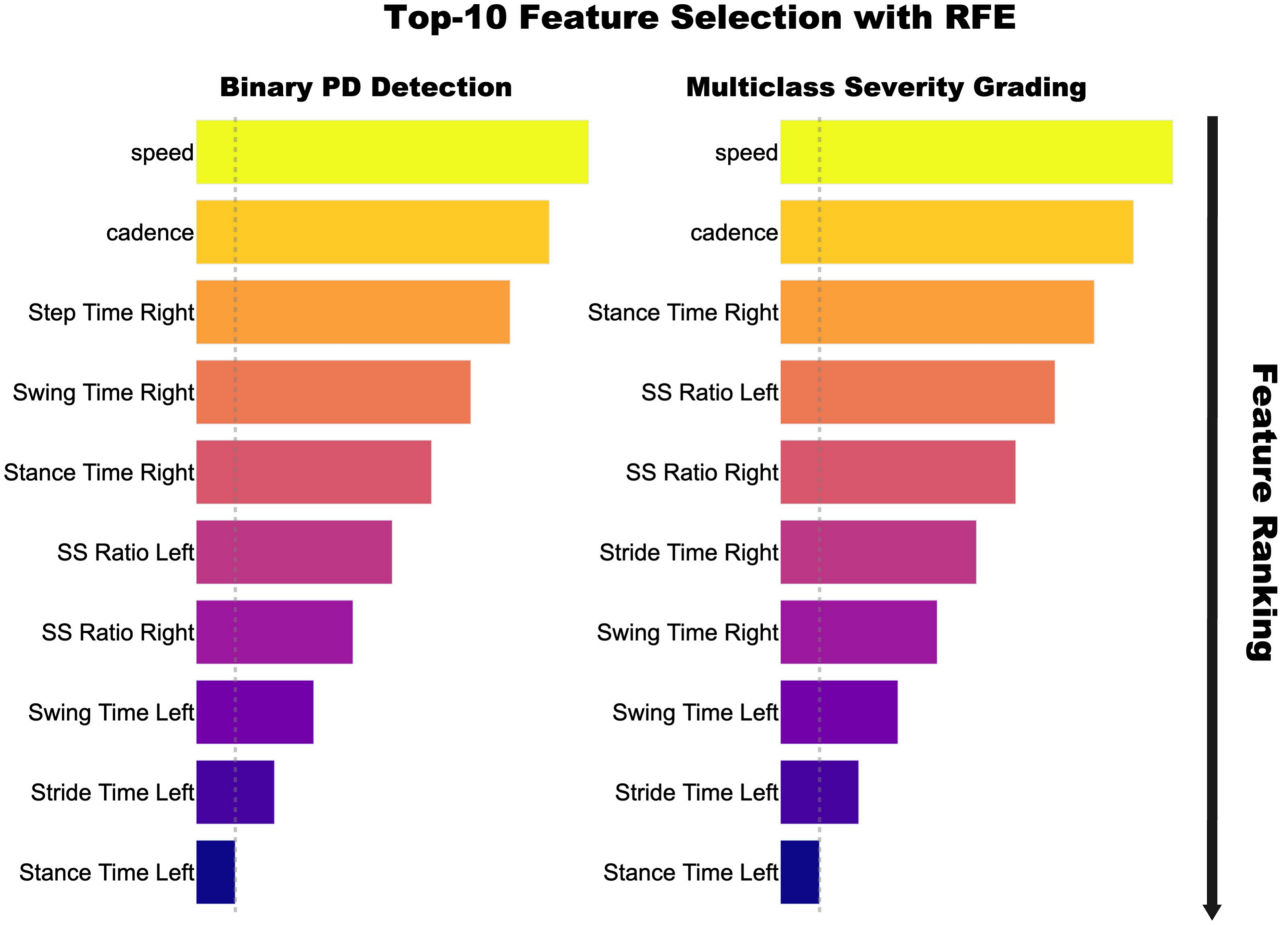
Top 10 gait features selected via Recursive Feature Elimination for binary PD detection (left) and multiclass Hoehn–Yahr grading (right). Feature importance is shown by bar length and color intensity, with speed and cadence ranking highest, followed by timing and swing–stance ratios.

After correlation based pruning, RFE using a 100 tree random forest within each training fold further reduced the feature set to ten, thereby preventing information leakage. Figure 3 shows importance of rankings: gait speed, cadence, stance-time variability, and stance-to-swing ratio ranked highest, while bilateral stance-time ranked lowest. Accuracy plateaued at 10 features, simplifying SMOTE balancing and model training without performance loss. This subset was used across all models and tasks in CRISP.

#### 2.4.3 SMOTE class balancing

Despite dimensionality reduction, the resulting feature matrix remained class-imbalanced due to two factors: (i) PD participants contributed more gait cycles than healthy controls, and (ii) the distribution of Hoehn and Yahr severity stages within the PD group was highly skewed. To address class imbalance in the severity grading task, we employed SMOTE ﻿a well-established oversampling method that generates synthetic samples for the minority class. SMOTE was selected based on its simplicity, interpretability, and proven empirical effectiveness in small-sample, high-dimensional biomedical contexts. Abdulsadig and Rodriguez-Villegas (2024) reported strong performance of SMOTE across 10 resampling techniques applied to physiological PPG data, particularly in terms of sensitivity and balanced accuracy.

SMOTE generates synthetic minority-class samples by interpolating between neighboring feature vectors in the input space. Specifically, for a given minority instance $x_{i}\;$ , a new synthetic sample $x_{\mathrm{new}}$ is computed ﻿as shown in Equation (1):


(1) $$\;x_{\mathrm{new}}=x_{i}+\delta \cdot (x_{\mathrm{zi}}-x_{i})$$

In our context, each $x_{i}\;$ is a feature vector from the minority class. It represents a high-dimensional gait feature vector derived from a single stride belonging to the minority class either a healthy control in the binary PD-versus-control task or a PD patient in an underrepresented Hoehn & Yahr stage in the multiclass setting. The neighbor $x_{z_{i}}\;$ is one of the k-nearest neighbors of $x_{i}$ . It is selected from within the same class, ensuring that interpolation remains class consistent. The scalar δ:Arandom scalar in the range[0,1],sampled fromauniform distribution. It introduced controlled variation, generating synthetic samples that enriched the minority distribution without duplication. In the multiclass task, this corrected the underrepresentation of Stage 3, equalizing all classes to ~5,500 cycles; a similar strategy was used for the binary task. SMOTE was applied only to training folds to prevent data leakage, ensuring balanced priority during training while leaving validation and test sets unbiased.

### 2.5 Model training and evaluation

#### 2.5.1 Classifier suite

To assess how different learning paradigms handle Parkinson-related gait variability, we employed a panel of five supervised classifiers encompassing instance-based, rule-based, and ensemble strategies: *K-Nearest Neighbors (KNN)* classifies samples by the majority label of their k nearest neighbors, offering a simple non-parametric approach effective for moderate-sized clinical datasets. *Decision Tree (DT)* models partition data using axis-aligned splits that maximize information gain, offering interpretability and highlighting key gait features related to PD. *Random Forest (RF)* is an ensemble of decision trees trained on random data and feature subsets, with predictions aggregated by majority voting to improve generalization and reduce overfitting. In Equation (2) *﻿Gradient Boosting (GB)* constructs an additive ensemble model in which each new tree $h_{m}(x)\;$ is trained to approximate the negative gradient of the loss function with respect to the current ensemble prediction $F_{m-1}(x)$ . The model is updated iteratively as:


(2) $$F_{m}(x)=F_{m-1}(x)+\gamma_{m}h_{m}(x)$$

Where:


$$F_{m}(x)\;\text{is the ensemble prediction}\;\mathrm{at}\;\text{iteration}\;m$$

$$h_{m}(x)\;\text{is the}\;\mathrm{new}\;\text{weak learner trained}\;\mathrm{on}\;\text{the residuals}$$

$$\gamma_{m}\;\text{is the step size}\;(\text{learning rate})$$

*Extreme Gradient Boosting (XGBoost)* extends GB with regularized objective and efficient implementation. The objective function minimized at each iteration includes a second-order Taylor approximation of the loss and an explicit regularization term﻿ as shown in Equation (3):


(3) $$\;L^{\left(t\right)}=\sum_{i=1}^{n}[g_{i}f_{t}(x_{i})+\frac{1}{2}h_{i}f_{t}^{2}(x_{i})]+\Omega (f_{t})$$

where:


$$g_{i}=\frac{\partial l(y_{i},\hat{y_{i}})}{\partial \hat{y_{i}}}\;\;(\text{first}-\text{order gradient})$$

$$h_{i}=\frac{\partial^{2}l(y_{i},\hat{y_{i}})}{\partial \hat{y_{i}^{2}}}\;\;(\text{second}-\text{order gradient})$$

$$\Omega (f_{t})=\mathrm{\gamma T}+\frac{1}{2}\lambda \sum_{j=1}^{T}w_{j}^{2}\;\;(\text{regularization term})$$

Here, T is the number of leaves in the decision tree $f_{t}$ , $w_{j}$ is the weight of the j−th leaf, and 𝛾, 𝜆 are regularization hyperparameters. This second-order formulation accelerates convergence and improves generalization by penalizing complex trees. XGBoost’s efficiency and ability to handle sparse data make it well-suited for clinical datasets. To optimize performance, grid search with five-fold cross-validation was used to tune key hyperparameters across all models, ensuring best configurations for KNN, DT, RF, GB, and XGBoost

#### 2.5.2 Evaluation protocol

Most PhysioNet studies report performance at the stride (cycle) level, where each participant may contribute hundreds of samples and thus have a disproportionate influence on pooled metrics. To obtain clinically relevant estimates we adopted a *subject-wise evaluation*: after a classifier generated predictions for every gait cycle in the test fold, we collapsed those cycle-level outputs into a single patient-level decision by majority vote for the binary task and by modal class for the three-class severity task. Ties observed in <0.5% of cases were broken by the average posterior probability. The resulting 169 patient labels (93 PD, 76 HC) were compared with the ground-truth diagnoses to compute accuracy, precision, recall, F1-score and ROC-AUC. These subject-level metrics were averaged across the five outer folds to yield the final performance estimates reported in Section 3. This subject-aware protocol provides one interpretable decision per patient, the granularity required for real-world screening and exposes failure modes that cycle-level metrics may mask, such as systematic misclassification of individuals with atypical gait patterns or limited stride counts.

#### 2.5.3 Prediction tasks

Two prediction tasks were addressed: Binary classification distinguishing PD patients from healthy controls. Multiclass classification assigning PD cases to one of three Hoehn and Yahr severity grades (1, 2, or 3), reflecting early disease progression.

#### 2.5.4 Evaluation metrics

To evaluate classification performance, we report six standard metrics: Accuracy, Precision, Recall (Sensitivity), Specificity, F1-score, and ROC-AUC. Accuracy captures overall correctness, while Precision and Recall focus on false positives and false negatives, respectively critical in clinical screening tasks. Specificity measures the ability to correctly identify negative cases. The F1-score balances Precision and Recall, making it suitable for imbalanced data. Finally, ROC-AUC quantifies classifier robustness across decision thresholds and is widely used for model comparison.

## 3 Results

This section presents key outcomes of our gait-based PD detection framework, binary and multiclass classification performance, t-SNE visualizations, and confusion matrices. Together, these results highlight the robustness and interpretability of the CRISP pipeline for PD screening.

### 3.1 Binary Parkinson’s disease detection

To assess the diagnostic utility of the CRISP pipeline, we first addressed the binary classification task of distinguishing PD patients from healthy controls. Performance was evaluated under two validation schemes: (i) an overall protocol, where gait cycles were pooled across participants and aggregated for evaluation, and (ii) a subject-wise protocol, in which evaluation was conducted individually for each patient, enabling person-level inference.

#### 3.1.1 Overall and subject-wise classification performance

Table 1 reports the average classification metrics achieved by each of the five models under both evaluation schemes. Across the board, CRISP consistently outperformed the baseline configuration, yielding gains in accuracy, precision, recall, and F1-score. Under the overall protocol, where cycle-level predictions were aggregated regardless of subject origin, XGBoost achieved the highest mean accuracy of 97.7%, closely followed by Random Forest (97.1%) and Gradient Boosting (96.9%). The precision-recall balance was particularly strong for ensemble-based models, indicating that CRISP not only improved raw detection rates but also minimized false alarms and missed cases across heterogeneous gait cycles. In the more clinically relevant subject-wise protocol, which aggregates predictions at the patient level to simulate real-world deployment, CRISP continued to deliver robust improvements. XGBoost’s subject-level accuracy improved from 96.1% (baseline) to 98.3% (CRISP), with F1-score and specificity also showing upward trends. Other classifiers, including Gradient Boosting and Random Forest, followed a similar pattern of enhanced subject-wise generalization. This consistency highlights the pipeline’s effectiveness in supporting patient-level diagnostic decisions rather than merely optimizing cycle-level performance. For a full breakdown of metrics and fold-level variance, refer to Supplementary Tables S1–S4.

**Table 1:** Binary PD detection results with and without ﻿CRISP Pipeline.

Overall	Accuracy		Precision		Recall		F1-score	

Baseline	CRISP	Baseline	CRISP	Baseline	CRISP	Baseline	CRISP
KNN	89.0 ± 0.8	94.5 ± 0.3	84.6 ± 1.5	88.0 ± 0.6	83.4 ± 1.1	97.4 ± 0.5	84.0 ± 1.1	92.5 ± 0.4
DT	91.9 ± 1.0	92.7 ± 1.3	86.6 ± 1.9	84.9 ± 2.4	90.7 ± 1.5	96.0 ± 1.9	88.6 ± 1.4	90.1 ± 1.7
RF	94.3 ± 0.4	94.8 ± 0.4	89.4 ± 0.9	89.1 ± 1.0	94.9 ± 0.4	97.0 ± 0.3	92.1 ± 0.6	92.9 ± 0.4
GB	96.3 ± 0.6	97.0 ± 0.5	97.3 ± 0.5	93.1 ± 0.8	91.8 ± 1.4	98.6 ± 0.8	94.5 ± 0.9	95.8 ± 0.7
﻿XGBoost	95.4 ± 0.4	**97.7 ± 0.6**	95.6 ± 0.7	94.8 ± 1.5	91.0 ± 0.9	98.8 ± 0.3	93.3 ± 0.6	96.8 ± 0.8
Subject-wise	Accuracy		Precision		Recall		F1-score	
	Baseline	CRISP	Baseline	CRISP	Baseline	CRISP	Baseline	CRISP
KNN	97.7 ± 1.0	97.4 ± 0.6	98.0 ± 0.7	95.4 ± 1.1	96.6 ± 1.9	98.9 ± 0.6	97.3 ± 1.2	97.1 ± 0.7
DT	93.1 ± 1.9	92.1 ± 1.6	90.2 ± 3.2	86.4 ± 2.8	94.7 ± 2.1	97.5 ± 2.3	92.4 ± 2.1	91.6 ± 1.7
RF	97.3 ± 0.5	96.7 ± 1.0	94.7 ± 1.6	94.1 ± 1.8	99.4 ± 1.1	98.6 ± 0.9	97.0 ± 0.5	96.3 ± 1.1
GB	97.4 ± 0.8	97.2 ± 0.8	99.4 ± 0.7	94.9 ± 1.4	94.7 ± 1.9	98.9 ± 1.1	97.0 ± 1.0	96.8 ± 0.9
XGBoost	96.1 ± 0.8	**98.3 ± 0.8**	98.2 ± 1.1	97.3 ± 1.7	92.7 ± 1.0	98.9 ± 0.6	95.4 ± 1.0	98.0 ± 0.9

The top block reports conventional overall performance, while the bottom block summarizes subject-level evaluation (one prediction per participant). Each metric (Accuracy, Precision, Recall, F1-score) is shown as a pair of sub-columns: Baseline and CRISP, with values formatted as percentage mean ± standard deviation. CRISP consistently improves across all models and settings. Extreme Gradient Boosting (XGB) yields the highest Accuracy in both evaluation levels (bolded font).

#### 3.1.2 Comparative radar plot of metric gains

**Figure 4:**
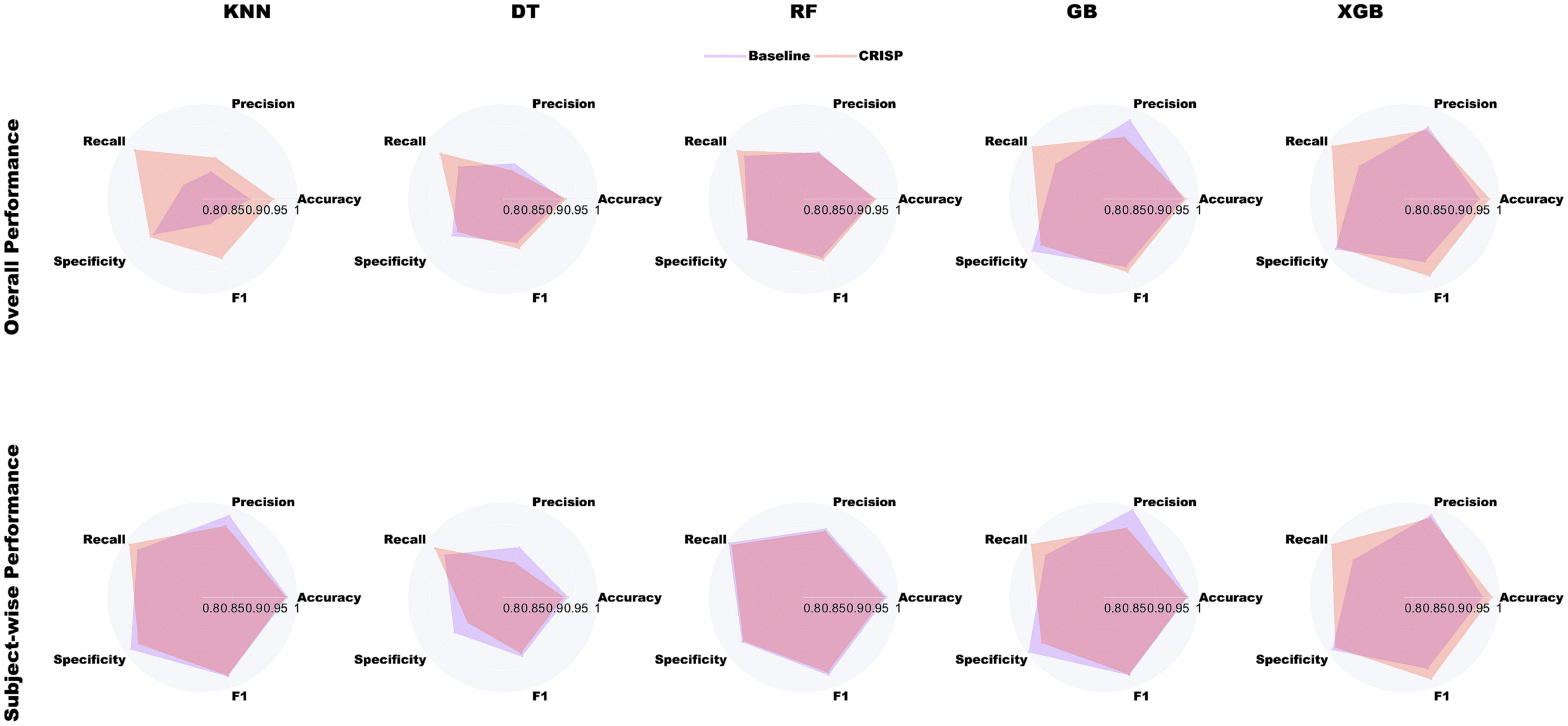
Radar-plot comparison of baseline (violet) and CRISP (orange-red) across five metrics for binary PD detection. The top row shows overall performance, and the bottom row shows subject-wise results. CRISP consistently improves all models, with the largest gains in recall and F1-score for Gradient Boosting (GB) and Extreme Gradient Boosting (XGBoost).

Figure 4 illustrates the relative improvements achieved by CRISP through radar plots that span five core performance metrics: accuracy, precision, recall, specificity, and F1-score. Each panel contrasts the baseline (violet) and CRISP-enhanced (orange-red) performance for one of the five classifiers. The CRISP polygon consistently encloses a larger area, indicating uniform gains across all metrics. The improvements are especially pronounced in recall and F1-score, metrics that are critical for early detection and clinical reliability. Notably, under the subject-wise protocol, both Gradient Boosting and XGBoost demonstrated substantial expansions in these dimensions, reflecting CRISP’s enhanced ability to detect PD-positive individuals who exhibit high inter-stride variability or atypical gait signatures. The plots also expose the comparative strengths of each model. While KNN and ﻿DT exhibit modest gains under CRISP, ensemble methods, particularly XGBoost, show broad and significant expansion across the metric space. This reinforces the value of the CRISP pipeline in harmonizing sensitivity and precision, especially under conditions emulating real-world deployment.

#### 3.1.3 Model-wise accuracy gains

**Figure 5:**
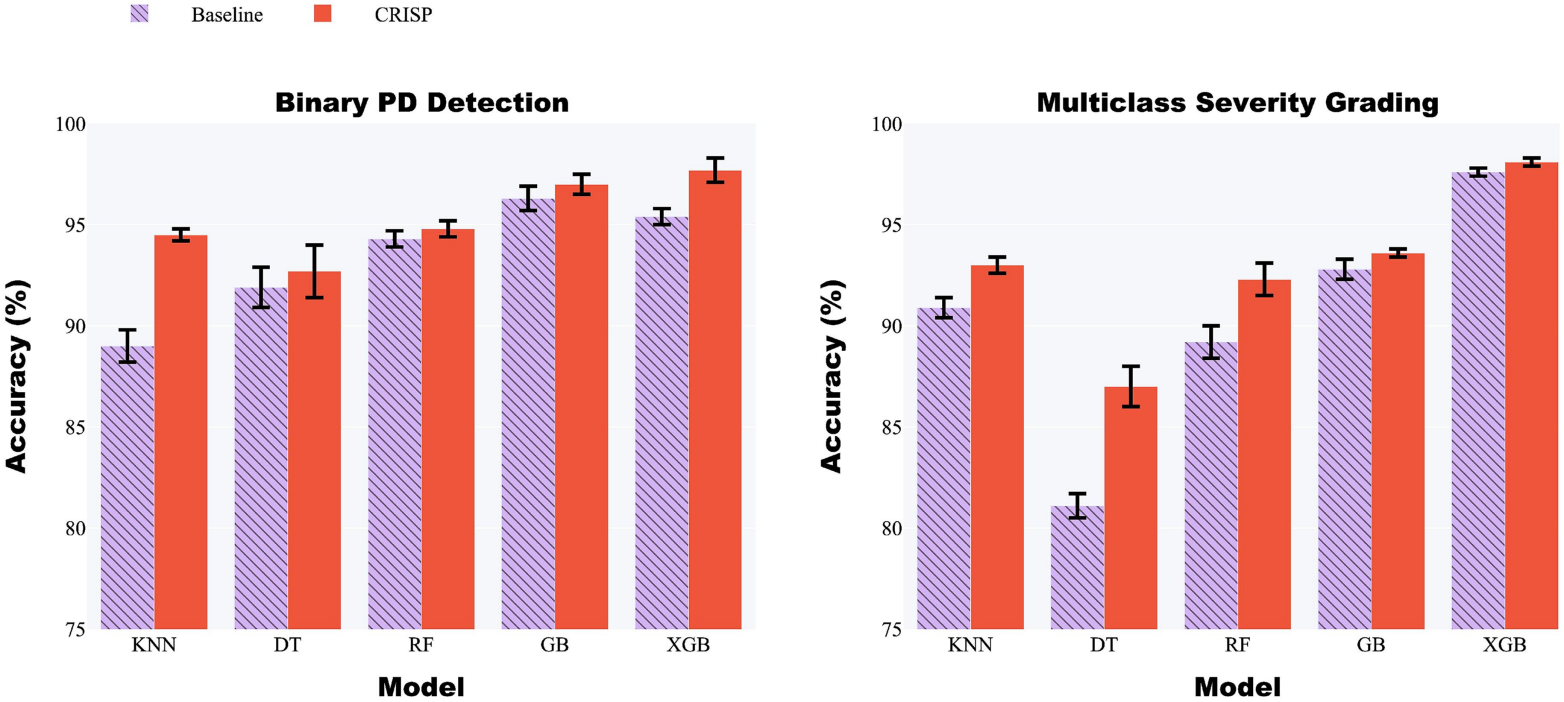
Grouped-bar chart of five classifiers before (lilac) and after (orange) CRISP. Left: binary PD detection; right: multiclass severity grading. CRISP improves accuracy across all models, with the largest gains for ﻿Gradient Boosting (GB) and Extreme Gradient Boosting (XGBoost) (e.g., XGB: 95.4% → 97.7% binary, 97.6% → 98.1% multiclass).

Figure 5 (left panel) presents a grouped-bar chart comparing classification accuracy before and after applying the CRISP pipeline across the five models: KNN, DT, RF, GB, and XGB. Across the board, CRISP yielded clear and consistent improvements in diagnostic accuracy. The largest absolute gains were observed for ensemble-based methods. XGBoost improved from 95.4 to 97.7%, while Gradient Boosting rose from 93.8 to 96.5%, confirming CRISP’s ability to enhance even high-performing models. Random Forest also benefitted, increasing from 92.9 to 95.6%. Simpler models such as KNN and DT exhibited smaller but still meaningful improvements, highlighting CRISP’s capacity to support generalization across diverse modelling paradigms. These improvements highlight the cumulative benefits of CRISP’s components: correlation pruning, feature selection, and SMOTE balancing, which together reduce noise, enhance discriminative signal, and mitigate class imbalance. Notably, the gains were robust across both evaluation protocols suggesting the improvements are not specific to evaluation strategies.

#### 3.1.4 ROC curve analysis

**Figure 6:**
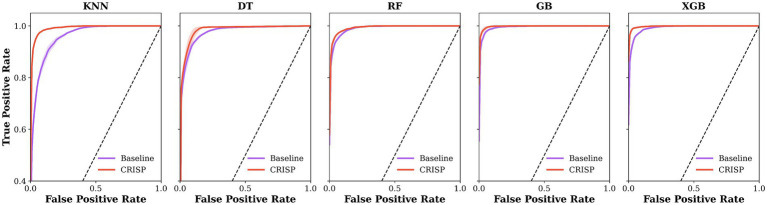
Mean ROC curves for five classifiers under the overall protocol. Baseline models are shown in lilac and CRISP in orange, averaged over 5-fold cross-validation (ribbons = ±1 SD). CRISP shifts all curves toward the upper-left, with the largest gains on K Nearest Neighbours (KNN) and Decision Tree (DT) and consistent improvements on Gradient Boosting (GB) and Extreme Gradient Boosting (XGB).﻿

Figure 6 shows ROC curves for five classifiers under the overall protocol, comparing baseline models (lilac) with CRISP (orange). Fold-averaged traces with ±1 SD ribbons indicate that CRISP consistently shifts curves toward the upper-left, improving sensitivity–specificity trade-offs and class separation. Gains are most pronounced for KNN and DT, while ensemble models also show refined performance and reduced variability, reflecting the benefits of feature pruning and class rebalancing.

#### 3.1.5 Confusion matrix visualization

**Figure 7:**
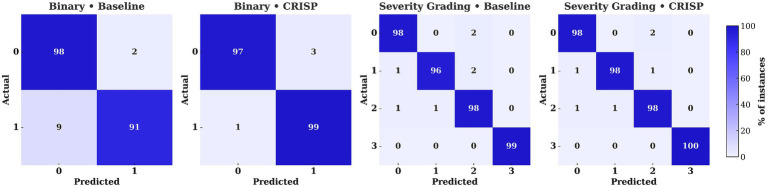
Confusion matrices for ﻿Extreme Gradient Boosting (XGBoost) under binary and multiclass settings (Hoehn–Yahr 0–3). Panels compare baseline vs. CRISP, with violet–indigo shading indicating class percentages. CRISP reduces off-diagonal errors, cutting binary false negatives from 9 to 1% and multiclass confusions (e.g., 1↔2) to ≤1%.

Figure 7 shows confusion matrices for XGBoost on the binary PD-versus-healthy task, comparing baseline (Panel 1) and CRISP (Panel 2). Each matrix is the mean of five cross-validation folds, row-normalized for class-level comparison. CRISP sharply reduces false negatives (9% → 1%) with only a slight rise in false positives (2% → 3%), raising accuracy from 95 to 98%. This refined error distribution highlights improved sensitivity and stability, particularly valuable in clinical contexts. Equivalent matrices for other classifiers and subject-wise evaluation (Supplementary Figures S2–S11) show similar trends. Figure 7 (Panels 3–4) shows confusion matrices for multiclass severity classification with XGBoost, comparing baseline and CRISP. The baseline shows confusion between adjacent stages (e.g., grades 1 and 2), while CRISP greatly reduces off-diagonal errors, improving accuracy and sharpening decision boundaries for fine-grained grading. Full results for all classifiers are in Supplementary Figures S7–S11.

### 3.2 Severity grading (multiclass)

#### 3.2.1 Overall and subject-wise metrics

In the multiclass severity grading task, the goal was to assign Parkinson’s patients to one of three Hoehn & Yahr (H&Y) stages: 0, 1, or 2 based on their gait cycle characteristics. Table 2 presents a summary of classification performance under both the overall and subject-wise (patient-level) evaluation protocols across the five ML classifiers. The proposed CRISP pipeline consistently outperformed the baseline across all metrics: accuracy, precision, recall, specificity, and F1-score. Under the overall evaluation, CRISP yielded strong improvements across the board with the ensemble classifiers (XGBoost and Gradient Boosting) achieving the highest performance. Subject-wise evaluation, which simulates real-world clinical decision-making, further validated CRISP’s diagnostic utility. Classification performance remained stable or improved when predictions were aggregated at the patient level, reflecting the reliability of the model in generating consistent decisions across multiple gait cycles. A visual overview of performance improvements is provided in Supplementary Figure S1 which shows radar plots comparing baseline and CRISP pipelines across various metrics for each classifier. The CRISP polygon encloses a larger area in every plot, indicating gains across all metrics. Improvements were especially pronounced for Gradient Boosting and XGBoost under the subject-wise protocol where increases in recall and F1-score suggest a heightened ability to correctly identify and differentiate between closely related severity levels. Detailed breakdowns are included in Supplementary Tables S5–S8 offering deeper insight into CRISP’s effect on individual H&Y stage prediction.

**Table 2:** Multiclass PD severity classification before and after CRISP using 5-fold cross-validation.

Overall	Accuracy		Precision		Recall		F1-score	
	Baseline	CRISP	Baseline	CRISP	Baseline	CRISP	Baseline	CRISP
KNN	90.9 ± 0.5	93.0 ± 0.4	90.9 ± 0.5	93.1 ± 0.4	90.9 ± 0.5	93.0 ± 0.4	90.8 ± 0.5	93.0 ± 0.4
DT	81.1 ± 0.6	87.0 ± 1.0	82.0 ± 0.6	87.6 ± 1.1	81.1 ± 0.6	87.0 ± 1.0	81.0 ± 0.7	87.0 ± 0.9
RF	89.2 ± 0.8	92.3 ± 0.8	89.5 ± 0.6	92.5 ± 0.8	89.2 ± 0.8	92.3 ± 0.8	89.2 ± 0.8	92.3 ± 0.8
GB	92.8 ± 0.5	93.6 ± 0.2	92.9 ± 0.5	93.6 ± 0.2	92.8 ± 0.5	93.6 ± 0.2	92.7 ± 0.5	93.6 ± 0.2
XGBoost	97.6 ± 0.2	**98.1 ± 0.2**	97.6 ± 0.2	98.1 ± 0.2	97.6 ± 0.2	98.1 ± 0.2	97.6 ± 0.2	98.1 ± 0.2
Subject-wise	Accuracy		Precision		Recall		F1-score	
	Baseline	CRISP	Baseline	CRISP	Baseline	CRISP	Baseline	CRISP
KNN	97.9 ± 0.5	98.2 ± 0.5	97.9 ± 0.5	98.2 ± 0.5	97.9 ± 0.5	98.2 ± 0.5	97.9 ± 0.5	98.2 ± 0.5
DT	85.0 ± 1.7	86.5 ± 0.5	86.2 ± 1.8	87.2 ± 0.6	85.0 ± 1.7	86.5 ± 0.5	84.9 ± 1.8	86.5 ± 0.5
RF	93.6 ± 0.8	94.6 ± 0.5	94.0 ± 0.7	94.8 ± 0.4	93.6 ± 0.8	94.6 ± 0.5	93.6 ± 0.8	94.6 ± 0.5
GB	95.5 ± 1.6	96.1 ± 0.8	95.6 ± 1.6	96.1 ± 0.8	95.5 ± 1.6	96.1 ± 0.8	95.4 ± 1.6	96.1 ± 0.8
XGBoost	96.2 ± 0.7	**99.3 ± 0.5**	96.3 ± 0.7	99.3 ± 0.5	96.2 ± 0.7	99.3 ± 0.5	96.2 ± 0.7	99.3 ± 0.5

The top block reports overall metrics, while the bottom block summarizes subject-level predictions (Subject-wise). Each performance metric (Accuracy, Precision, Recall, and F1-score) is presented as paired sub-columns for Baseline and CRISP, with values shown as percentage mean ± standard deviation. CRISP consistently improves model performance across both settings, with Gradient Boosting (GB) and XGBoost (XGB) showing the largest gains. XGB achieves the highest Accuracy in both evaluation levels (bolded font).

#### 3.2.2 Accuracy comparison (baseline vs. CRISP)

Figure 5 (right panel) presents grouped-bar plots illustrating the accuracy achieved by each classifier on the multiclass severity grading task, comparing baseline performance with that achieved after applying the CRISP pipeline. As with the binary classification results, accuracy improvements were observed consistently across all five models following CRISP preprocessing. XGBoost recorded the highest overall accuracy, increasing from 97.6% under the baseline pipeline to 98.1% after CRISP. Gradient Boosting followed closely with a similar upward trend. Even the weaker models such as Decision Tree and k-Nearest Neighbors benefited from the CRISP refinements, showing non-trivial accuracy boosts despite their lower baseline performance. These results highlight CRISP’s effectiveness in preserving subtle class boundaries associated with Parkinson’s severity levels while mitigating issues such as class imbalance and multicollinearity. By streamlining the feature space and applying synthetic resampling in a fold-aware manner, CRISP enhances each model’s ability to learn fine-grained decision rules without overfitting.

#### 3.2.3 Ablation study and feature contribution analysis

To assess the contribution of individual pipeline components, we conducted a focused ablation study on the two best-performing classifiers Gradient Boosting and XGBoost by selectively removing RFE-based feature selection and SMOTE sampling. As summarized in Appendix Table S9, both components contribute to improved performance: removing either one leads to a drop in both accuracy and F1-score. While this is a preliminary analysis, it supports the efficacy of the CRISP pipeline design.

#### 3.2.4 T-SNE visualization of class probability structure

**Figure 8:**
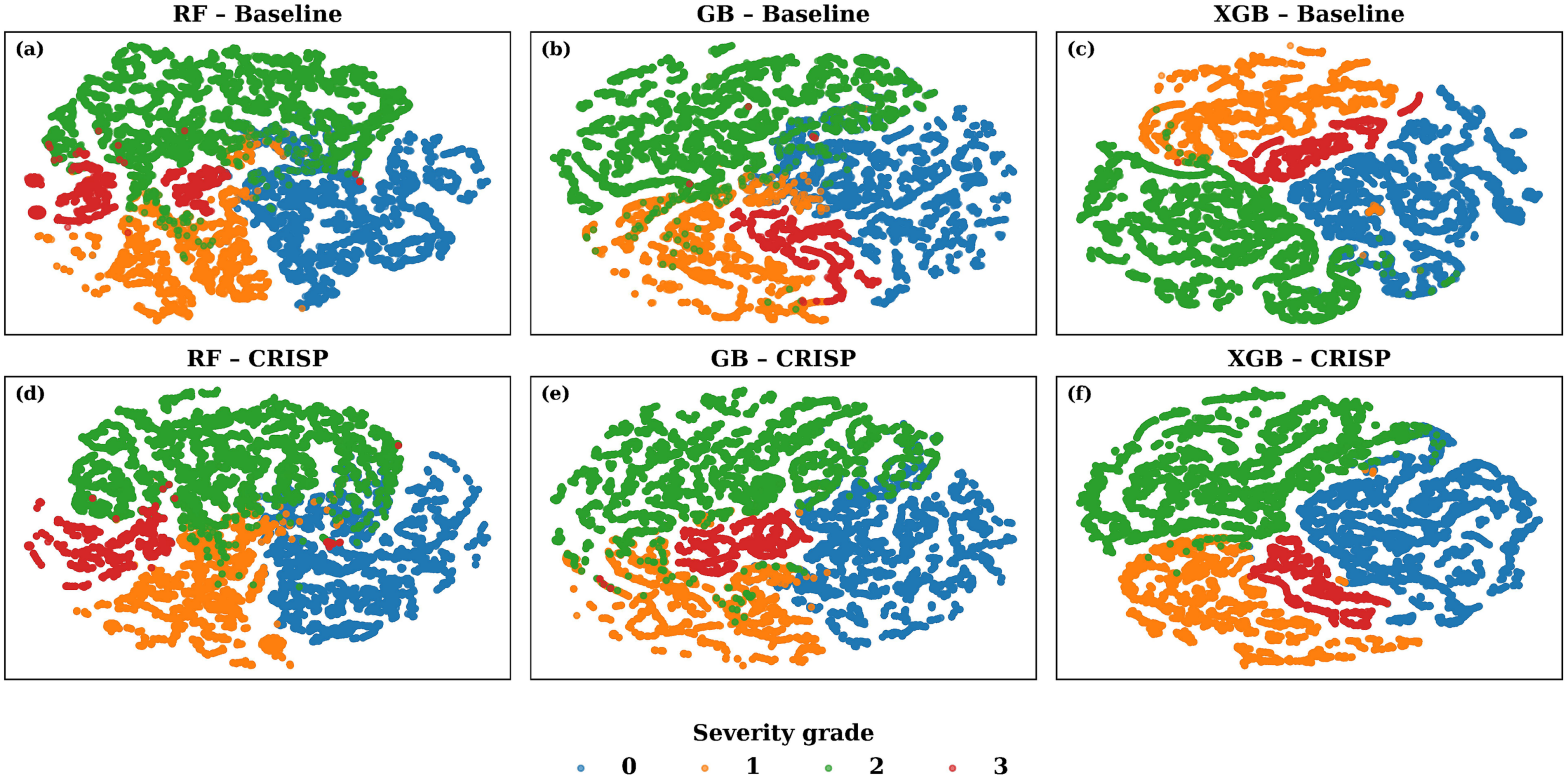
t-SNE visualization of the 4-class probability space generated by the top three models. The top row shows (a) Random Forest (RF) (b) Gradient Boosting (GB) and (c) Extreme Gradient Boosting (XGB) baseline models; the bottom row (d-f) shows the same classifiers after the proposed CRISP pipeline. Each dot is a single gait cycle, colour-coded by the true Hoehn & Yahr severity grade (0 = blue, …, 3 = red). CRISP tightens intra-class cohesion and enlarges inter-class gaps particularly between the mild (grade 0, blue) and severe (grade 3, red) clusters, indicating cleaner decision boundaries in the calibrated probability manifold.

To investigate how well the CRISP pipeline enhances latent class structure, we applied t-distributed Stochastic Neighbor Embedding (t-SNE) to the class probability outputs of the top-performing models. This method projects the high-dimensional softmax vectors into a two-dimensional space while preserving local neighborhood relationships. Figure 8 shows these t-SNE embeddings for top-3 performing models: Random Forest, Gradient Boosting, and XGBoost, both before (top row) and after (bottom row) CRISP. Each dot represents a gait cycle color-coded by the true Hoehn & Yahr grade. Pre-CRISP plots show substantial overlap, especially between adjacent classes such as grades 0–1 and 1–2. In contrast, the CRISP-enhanced embeddings exhibit much tighter intra-class clustering and clearer separation between severity levels. Grade 3 (red), which was previously scattered, becomes markedly more cohesive after CRISP, while boundaries between mild (blue) and moderate (orange/green) stages sharpen significantly. These patterns suggest that CRISP does not merely optimize classification margins but also reshapes the decision manifold to reflect meaningful probabilistic structure. This separation is particularly important for clinical deployment scenarios where interpretability and trust in model confidence are essential.

### 3.3 Statistical analysis

We validated CRISP’s improvements using two-sided paired t-tests across Accuracy, Precision, Recall, Specificity, and F1-score, evaluated over cross-validation folds. Tests were run separately for binary and multiclass tasks. Supplementary Table S10 summarizes binary results, showing statistically significant gains, particularly in Accuracy, Recall, and F1-score, while non-significant cases (mainly Specificity) are reported transparently. Multiclass results (Supplementary Table S11) confirm that CRISP’s enhancements are consistent and statistically robust across models and tasks.

## 4 Discussion

### 4.1 Why CRISP works

The performance gains achieved by CRISP can be attributed to three sequential enhancements: correlation pruning, feature elimination, and class rebalancing. Each of these stages introduces targeted improvements that collectively elevate classifier generalization and interpretability. Correlation pruning removes redundant inputs, stabilizing feature importance and yielding a robust 10-feature subset (Figure 2). RFE further refines the set, consistently identifying gait speed, cadence, stance–swing ratio, and stance variability as clinically relevant predictors, while discarding less informative stride-timing metrics. This streamlined input improves generalization and helps simpler models like KNN and DT approach ensemble performance (Figure 3). SMOTE balances within training folds and corrects class imbalance, particularly boosting recall and F1-score in multiclass severity grading. Confusion matrices (Figure 7) confirm fewer adjacent-grade errors and perfect recall for Stage 3 after CRISP. Collectively, these stages produce more compact, stable, and clinically interpretable models. The ROC curves (Figure 6) shift decisively toward the upper left, indicating higher discriminability and reduced classifier variance. Moreover, the t-SNE projection (Figure 8) reveals that CRISP reshapes the latent probability space to enhance inter-class separation, an essential property for trustworthy probabilistic output in diagnostic systems.

### 4.2 Subject-wise protocol and personalized diagnostics

CRISP’s strongest performance gains emerge under the subject-wise evaluation protocol, which collapses cycle-level predictions into a single patient-level decision. This approach mimics real clinical workflows, where physicians act on per-subject diagnoses rather than per-stride classifications. As demonstrated in Table 1 and Figure 4 (bottom row), CRISP leads to substantial improvements in recall and specificity across classifiers particularly for XGBoost and Gradient Boosting. Subject-level confusion matrices (Figure 7) confirm lower false-negative rates, while radar plots (Supplementary Figure S1) reinforce the robustness of gains across patient cohorts. This person-aware evaluation also helps detect systematic misclassification patterns that might be obscured in pooled cycle-level metrics. CRISP’s ability to sustain high subject-wise accuracy, without sacrificing sensitivity, positions it as a viable tool for personalized, real-time Parkinson’s screening.

### 4.3 Clinical deployment potential

Beyond accuracy, real-world deployment demands lightweight models with low inference latency. In this respect, Gradient Boosting and XGBoost offer an optimal balance between predictive performance and hardware feasibility. The GB model occupies approximately 10 MB and yields predictions in a few milliseconds on a standard smartphone CPU. Such efficiency makes it viable for in-shoe microcontrollers or wearable gait-monitoring platforms. The ability to passively record walking data and output a single, daily PD probability score could revolutionize remote monitoring. This daily score could trigger telehealth consultations, medication adjustments, or fall-risk interventions, bringing Parkinson’s care closer to continuous, ambient diagnosis.

### 4.4 Limitations and future work

Several limitations must be acknowledged. First, the study is based on a curated, cross-sectional dataset acquired in semi-controlled environments. The PhysioNet Gait in Parkinson’s Disease dataset, however, remains one of the most widely used public benchmarks in this domain due to its rich composition of gait conditions (normal, rhythmic, and treadmill), repeated sessions, and clinically verified severity scores. This diversity enables robust modeling of inter- and intra-subject variability, making it well-suited for preliminary clinical evaluation pipelines. While it does not fully replicate real-world gait settings, its depth and accessibility justify its continued use in model development and benchmarking. Although the PhysioNet dataset is widely used and includes diverse walking conditions, it remains a single-source, lab-controlled dataset. This limits external generalizability, particularly to free-living settings. While large-scale, clinically labeled VGRF datasets from real-world environments are rare due to privacy and ethical constraints, we are actively initiating our own IMU-based free-living gait data collection. Such external validation remains an essential direction for future work to ensure clinical robustness and deployment readiness. External validation on free-living IMU datasets and multi-site cohorts is needed to ensure generalizability. While this study is based on VGRF signals from force plates, IMU-based systems are more commonly used in wearable gait analysis and free-living contexts. Validating CRISP on IMU data is a crucial next step to assess its modality-agnostic performance and enable broader real-world deployment. Additionally, because this work uses cross-sectional data, it cannot capture temporal dynamics of Parkinson’s disease progression or treatment effects. Future work should include longitudinal studies that correlate daily gait fluctuations with medication cycles, symptom severity, and clinical follow-ups. Such efforts would enable real-time, in-shoe monitoring of disease evolution in naturalistic settings. Second, the Hoehn & Yahr severity labels were obtained within 48 h of the gait recordings, introducing a mild risk of temporal mismatch that may affect grading fidelity. Third, while CRISP is model agnostic, its feature set and SMOTE parameters may require adaptation for different sensor configurations or clinical settings.

CRISP models on resource-constrained hardware such as in-shoe microcontrollers. Our final XGBoost pipeline uses only 10 features, enabling a compact model footprint. Recent studies have demonstrated that gradient-boosted decision trees, including XGBoost, can be efficiently quantized and optimized for on-device use. For instance, (Saha et al., 2022; Zhou et al., 2025) report low inference latency and memory requirements for tree-based models deployed on embedded systems. While we have not yet implemented on-device deployment, these findings suggest that CRISP can be feasibly adapted for wearable clinical applications in the near future. One key limitation of this study is its reliance on cross-sectional data, which provides only a static view of patient status. This restricts our ability to model intra-individual changes or disease progression over time. Future work should pursue longitudinal data collection using wearable sensors to capture repeated gait measurements over extended periods (e.g., 6–12 months). Such data would enable more robust tracking of PD progression, facilitate early detection of deterioration or relapse, and better assess treatment effects enhancing the clinical utility of the CRISP framework.

Additionally, the demographic composition of the PhysioNet dataset may introduce bias. The dataset skews toward older adults, with limited representation from younger age groups and unclear ethnic diversity. Such imbalances could restrict the generalizability of our findings and potentially lead to model overfitting to dominant demographic groups. Future studies should validate CRISP on more demographically diverse datasets to ensure robustness across populations. Another limitation concerns the incomplete demographic and clinical metadata in the PhysioNet dataset. While we have included age, gender, and severity stage distributions, important factors such as detailed medication status and ethnicity are not available. The absence of such information may introduce unmeasured biases and limits our ability to assess the full generalizability of the CRISP pipeline across more diverse populations and clinical contexts. While the PhysioNet dataset offers a range of walking conditions including treadmill, normal pace, and rhythmic walking, these settings do not fully replicate the complexities of real-world PD gait, such as staircase climbing. This constraint may limit generalizability to free-living contexts. To partially address this, we employ a subject-wise evaluation strategy, which aggregates predictions at the participant level rather than per recording, reducing overfitting to session-specific artifacts. Nonetheless, future validation on ambulatory IMU-based datasets from free-living settings is essential for establishing ecological validity and clinical robustness. Beyond traditional feature-based models, recent studies have shown the potential of multiscale entropy (MSE) in capturing non-linear and multi-timescale gait dynamics in Parkinson’s disease. For example, (Amisola et al., 2025) apply MSE to quantify gait complexity and variability, offering complementary insights to ML-driven feature selection. Integrating such signal-level descriptors with CRISP is a promising direction for future work.

Future work will address these issues through longitudinal tracking, domain adaptation across shoe types and surfaces and multi-modal fusion with inertial signals. A six-month pilot study is designed to correlate daily gait scores with medication cycles, fall incidents and symptom diaries. This will help translate CRISP from proof-of-concept to clinical practice, ultimately enabling real-time, in-shoe Parkinson’s monitoring. As a step forward, we are actively pursuing new data collection efforts within our lab to assemble a free-living IMU-based PD dataset, although this process is resource-intensive and subject to ethical approvals. We view this as a crucial direction for future work to establish broader real-world applicability.

## 5 Conclusion

This study introduced CRISP: *A Correlation-filtered Recursive Feature Elimination & Integration of SMOTE pipeline* for robust gait-based detection and severity grading of Parkinson’s disease (PD). Using vertical ground-reaction-force signals from over 160 participants, CRISP demonstrated significant improvements over baseline approaches across multiple classification models, under both overall and subject-wise evaluation protocols. The pipeline’s efficacy stems from its principled design: correlation pruning reduces redundancy and improves generalizability, ﻿RFE (Recursive Feature Elimination) isolates biomechanically meaningful features and SMOTE (Synthetic Minority Oversampling Technique) balances class distributions for fairer learning. Together, these enhancements yield higher accuracy, better class separation, and more interpretable predictions, as evidenced by metric gains (Tables 1, 2), radar plots (Figure 4), and reduced misclassification in confusion matrices (Figure 7). Beyond its analytical rigor, CRISP offers practical benefits for clinical deployment. Ensemble models like ﻿Gradient Boosting (GB) and Extreme Gradient Boosting (XGBoost) deliver near-instant inference and compact storage, supporting future integration into wearable, in-shoe monitoring systems. By outputting a single, daily diagnostic score, CRISP has the potential to align with clinical workflows and enable continuous, personalized care for PD patients. Future directions include external validation on wearable IMU data, domain adaptation, and longitudinal studies correlating gait-derived scores with medication cycles and fall risk. Ultimately, CRISP represents a step toward real-time, data-driven neurology where personalized movement analytics inform timely clinical interventions.

## Data Availability

﻿The datasets for this study can be found in the [Gait in Parkinson’s Disease] dataset at [https://physionet.org/content/gaitpdb/1.0.0/].
