# Supplementary figures and images for: CRISP: a correlation-filtered recursive feature elimination and integration of SMOTE pipeline for gait-based Parkinson’s disease screening
Source: Front Comput Neurosci. 2025 Oct 10;19:1660963. doi: 10.3389/fncom.2025.1660963 (PMC12549659; doi:10.3389/fncom.2025.1660963)

Binary • Baseline

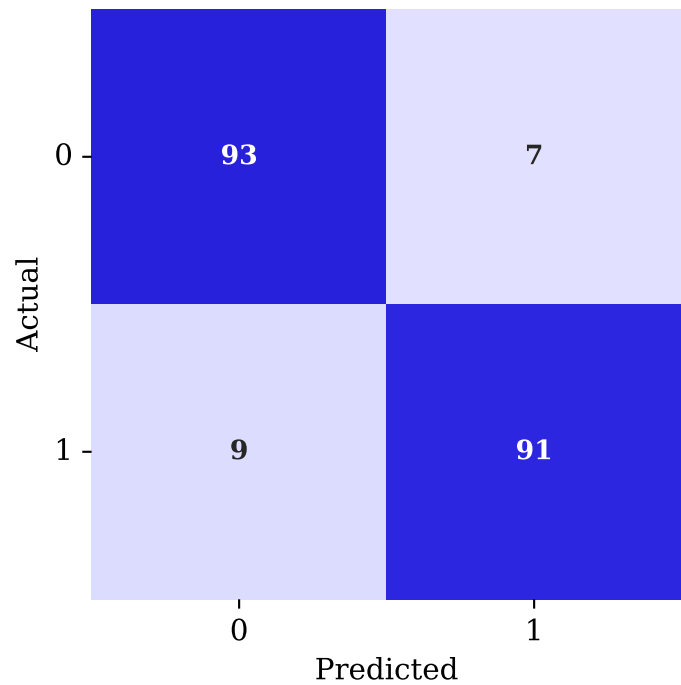

Binary • CRISP

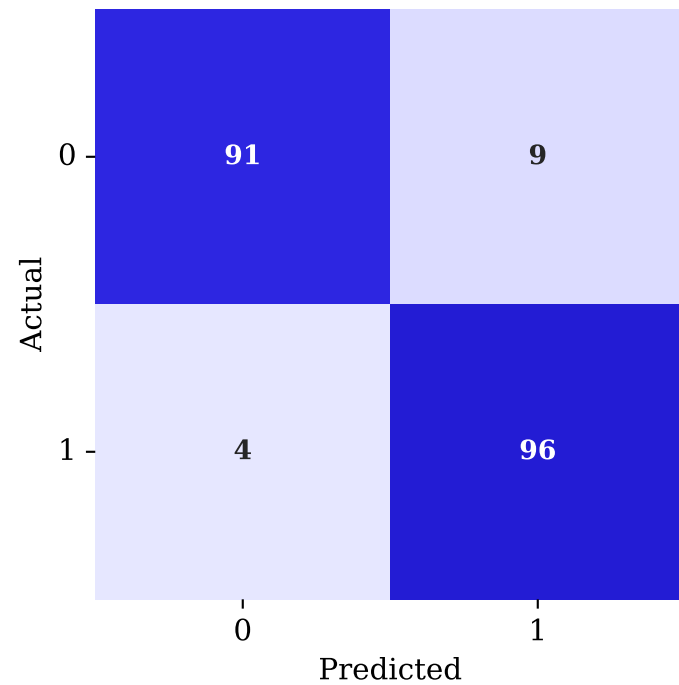

Multiclass • Baseline

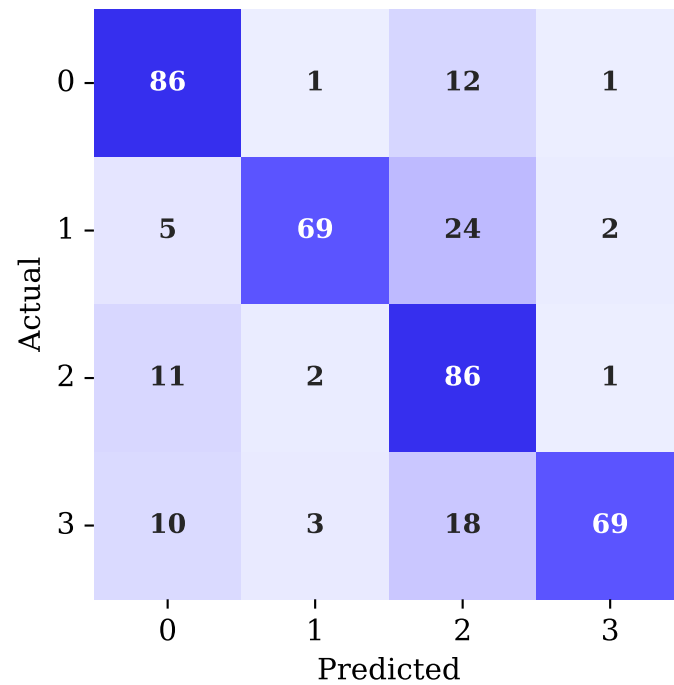

Multiclass • CRISP

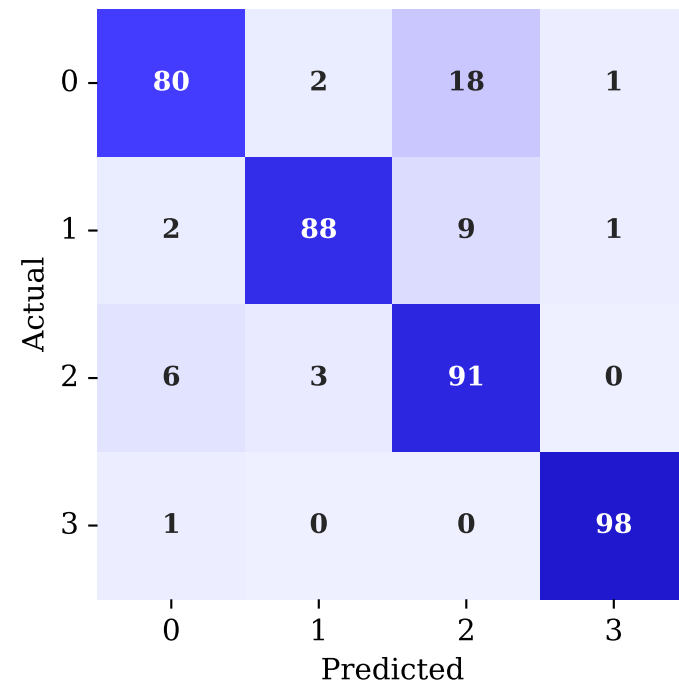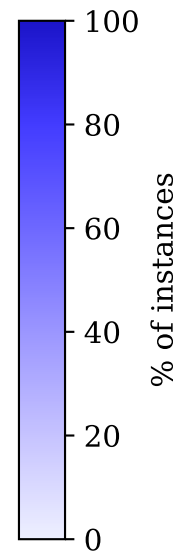

Supplement: Supplementary file 1 [file Data_Sheet_1.zip › confusion_matrices/dt/dt_overall_confusion_matrices_row.pdf]

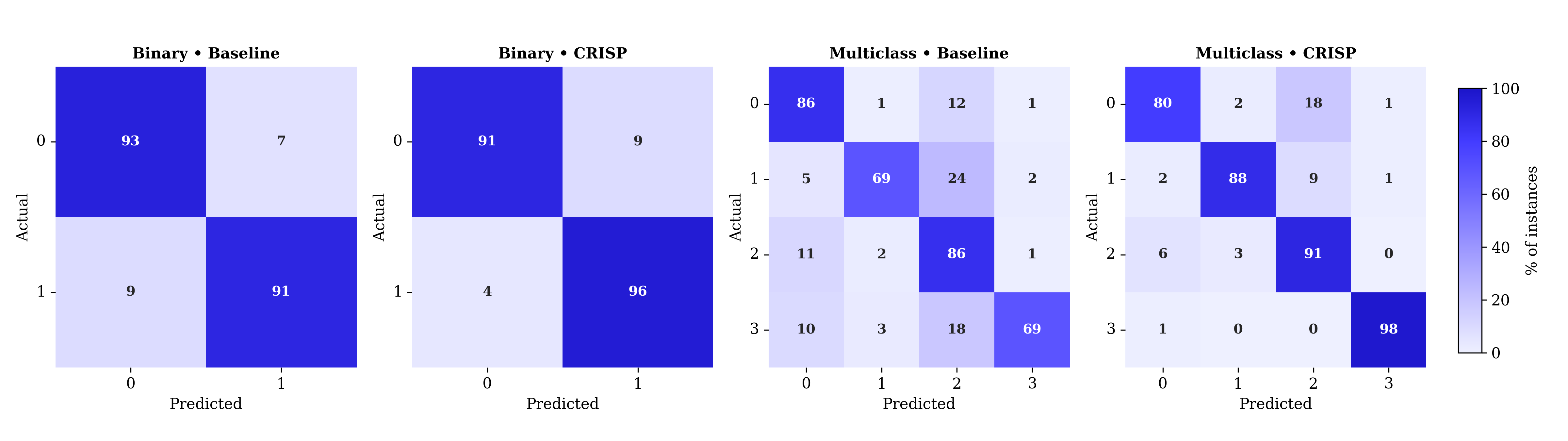

Supplement: Supplementary file 1 [file Data_Sheet_1.zip › confusion_matrices/dt/dt_overall_confusion_matrices_row.png]

Binary • Baseline

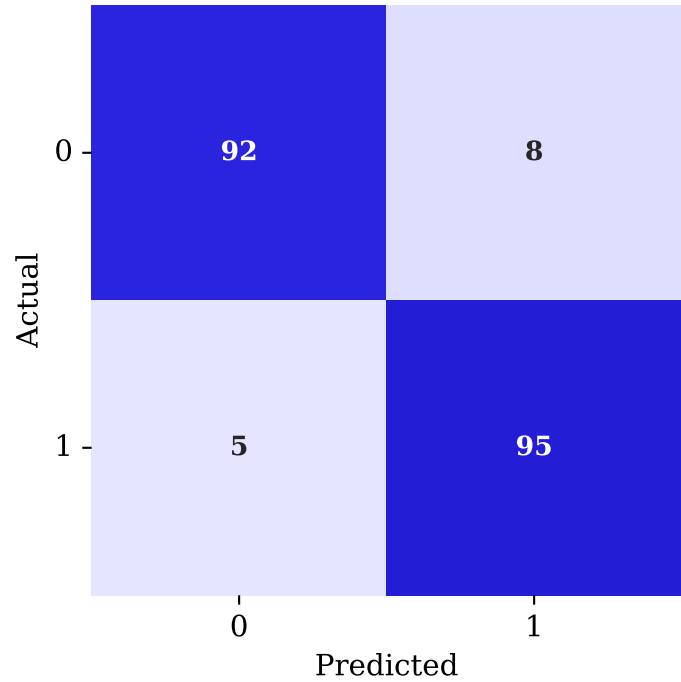

Binary • CRISP

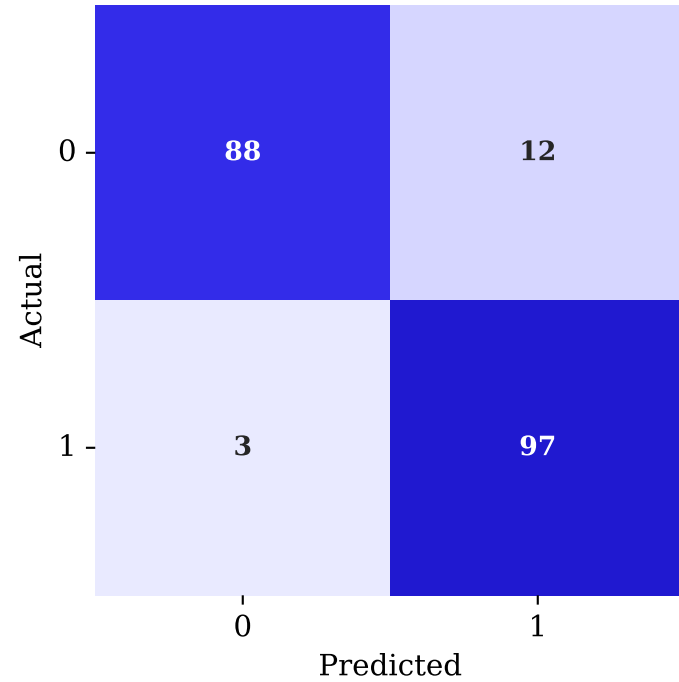

Multiclass • Baseline

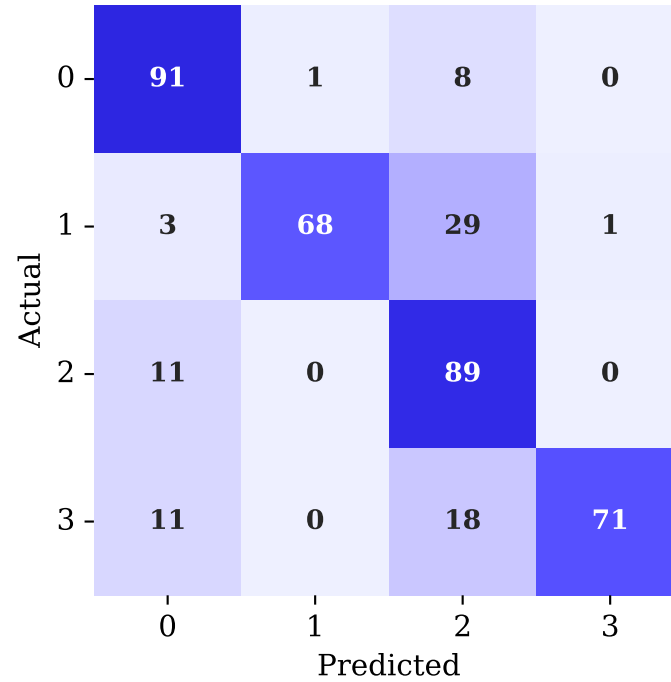

Multiclass • CRISP

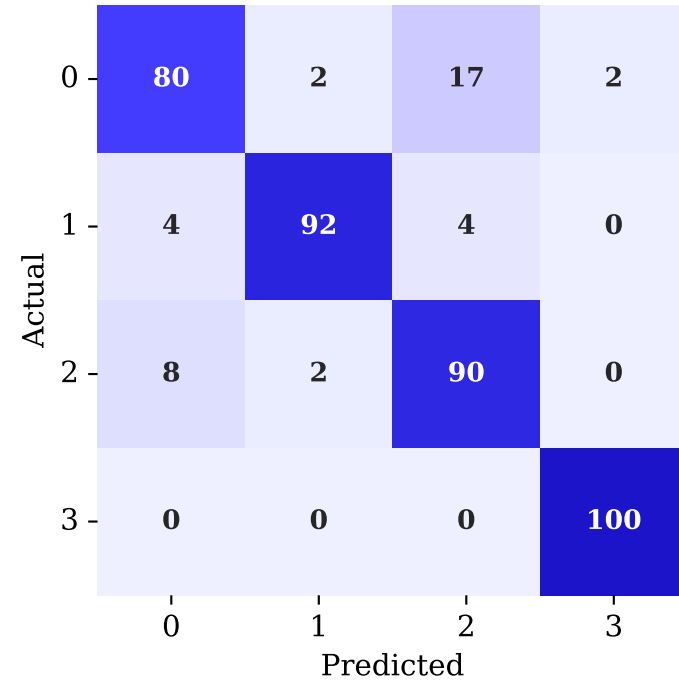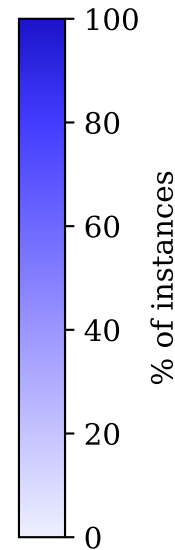

Supplement: Supplementary file 1 [file Data_Sheet_1.zip › confusion_matrices/dt/dt_subjectwise_confusion_matrices_row.pdf]

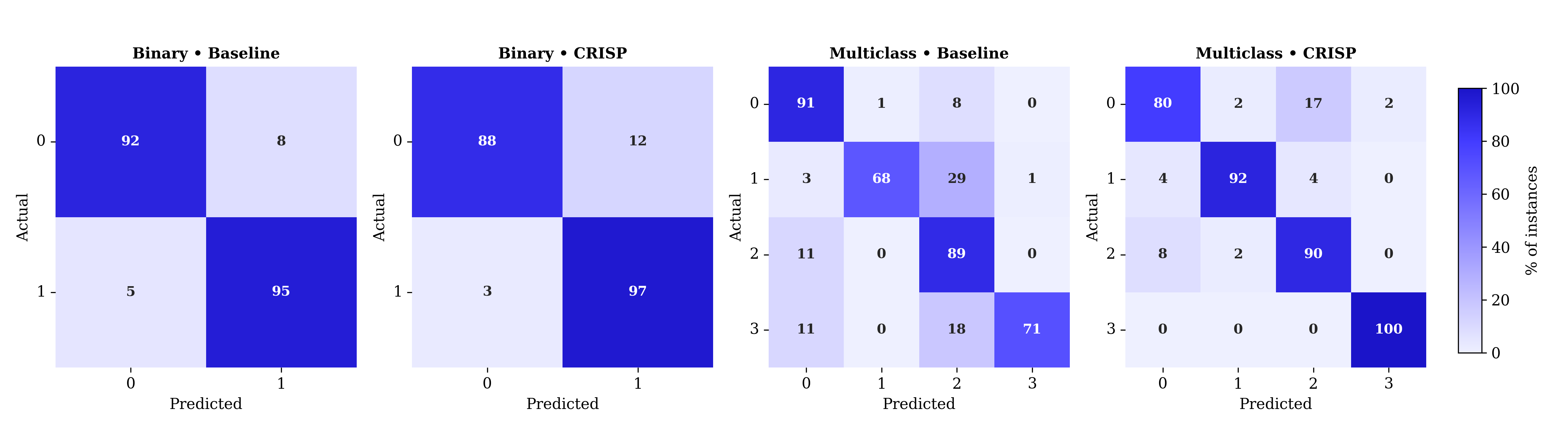

Supplement: Supplementary file 1 [file Data_Sheet_1.zip › confusion_matrices/dt/dt_subjectwise_confusion_matrices_row.png]

Binary • Baseline

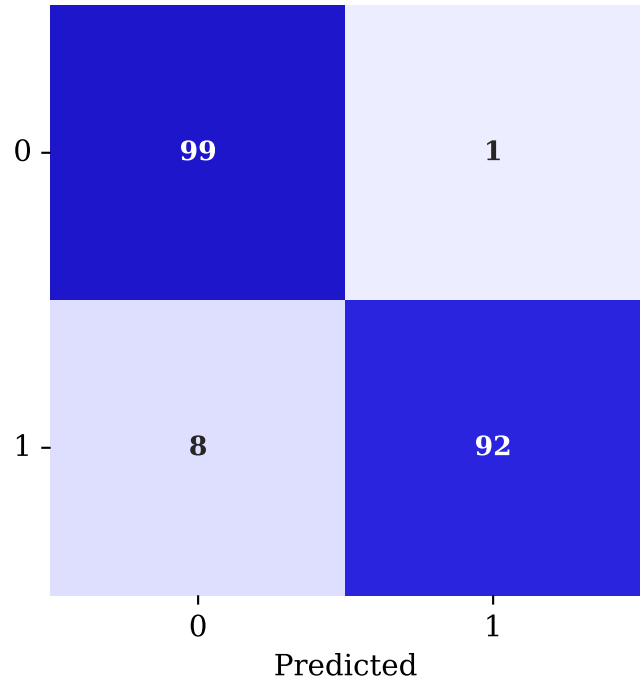

Binary • CRISP

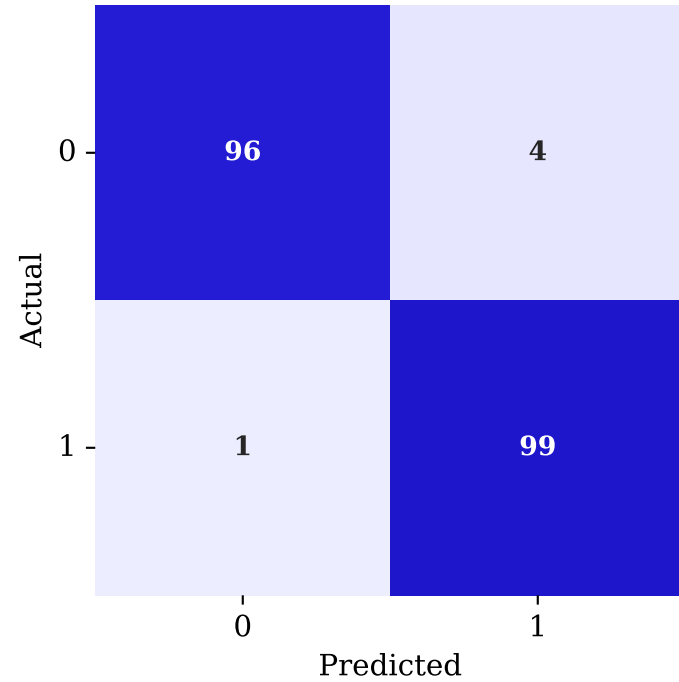

Multiclass • Baseline

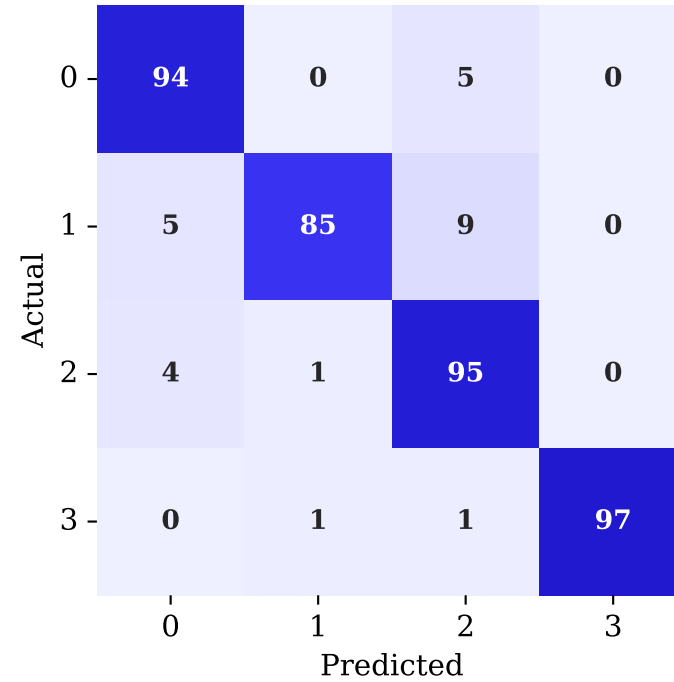

Multiclass • CRISP

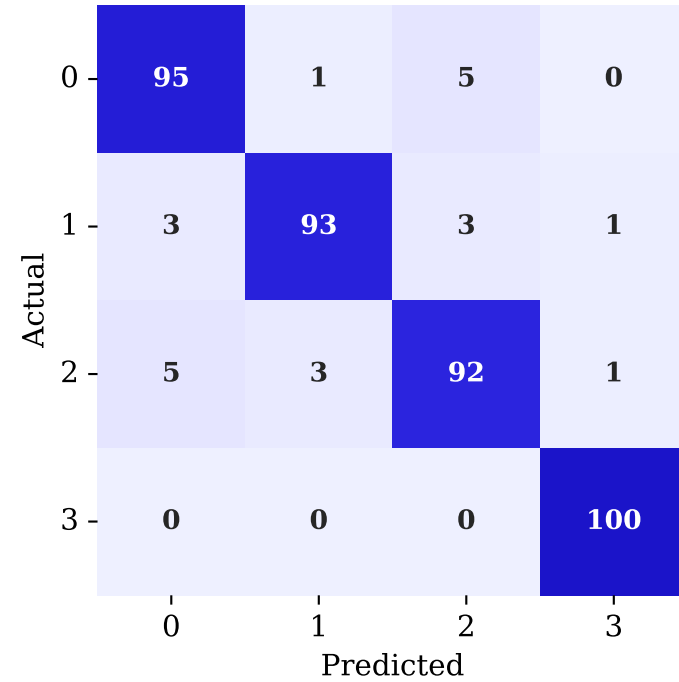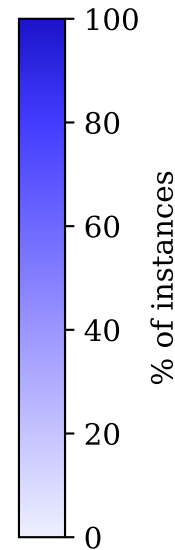

Supplement: Supplementary file 1 [file Data_Sheet_1.zip › confusion_matrices/gb/gb_overall_confusion_matrices_row.pdf]

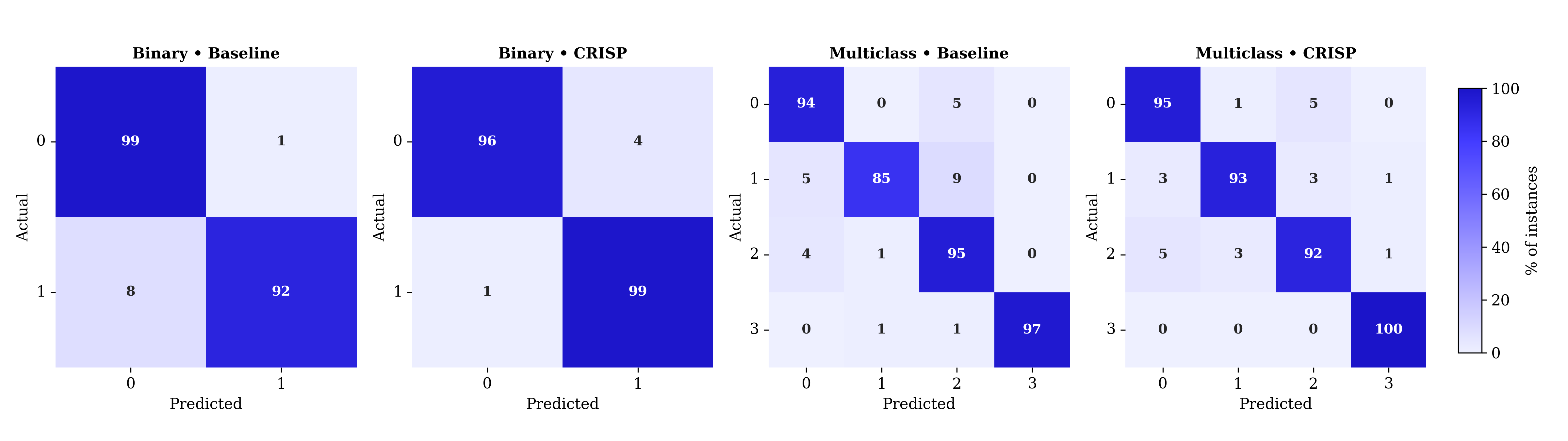

Supplement: Supplementary file 1 [file Data_Sheet_1.zip › confusion_matrices/gb/gb_overall_confusion_matrices_row.png]

Binary • Baseline

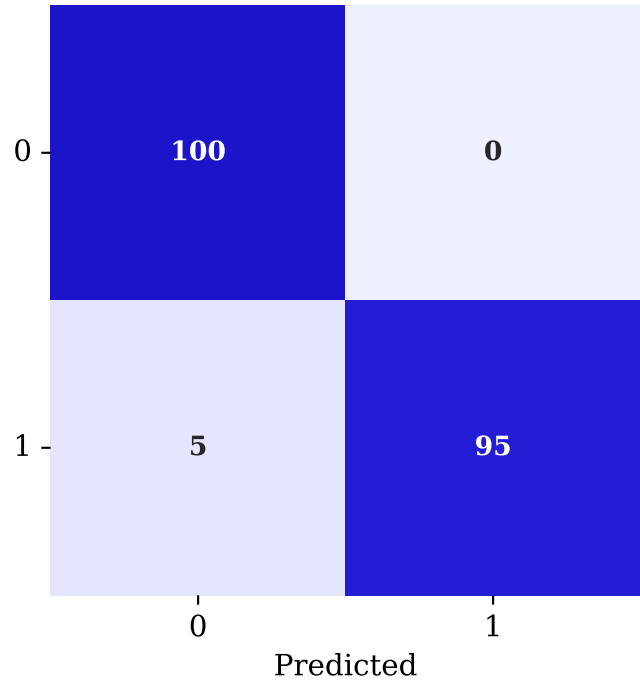

Binary • CRISP

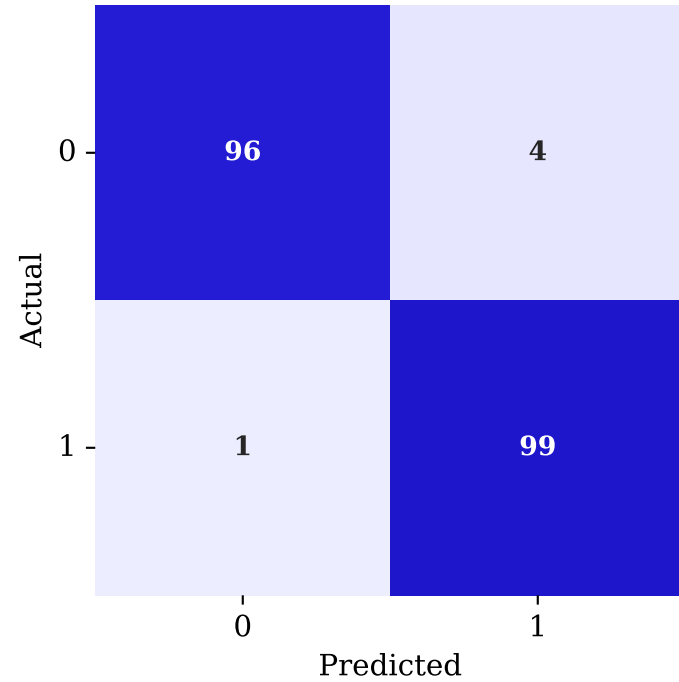

Multiclass • Baseline

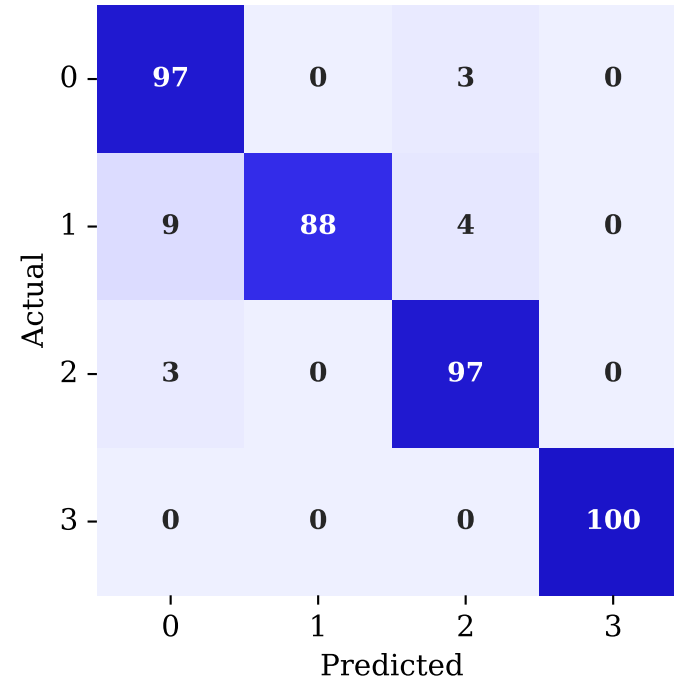

Multiclass • CRISP

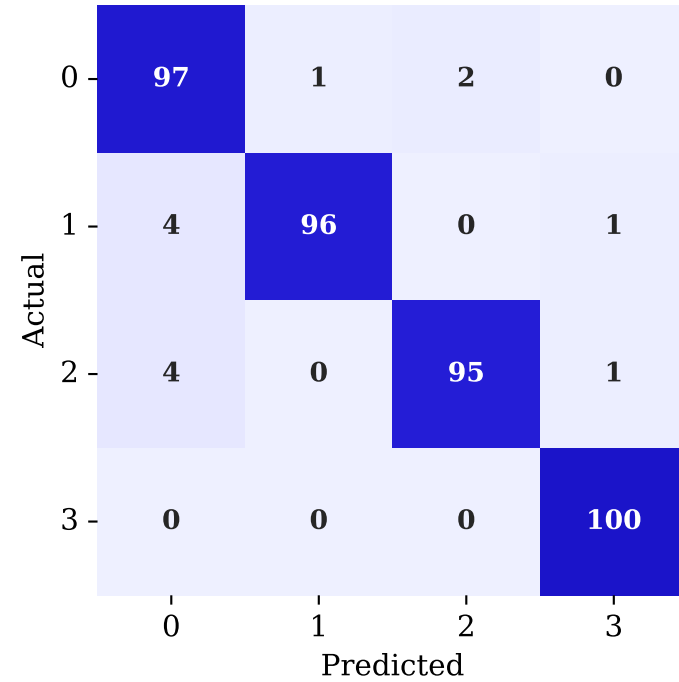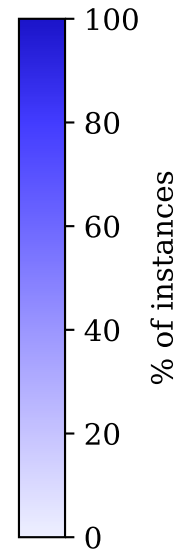

Supplement: Supplementary file 1 [file Data_Sheet_1.zip › confusion_matrices/gb/gb_subjectwise_confusion_matrices_row.pdf]

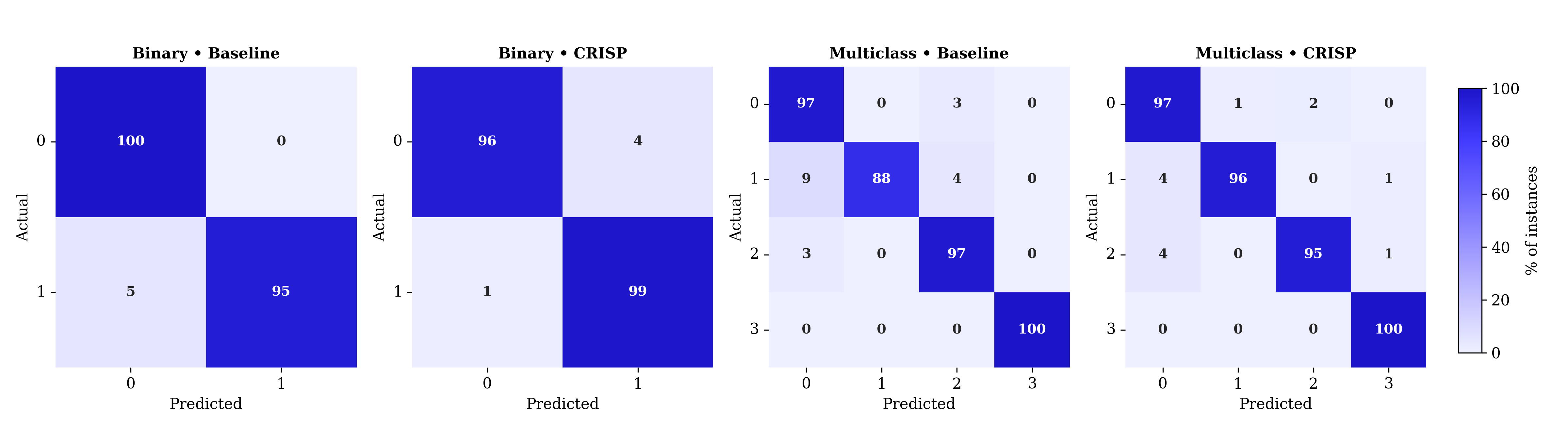

Supplement: Supplementary file 1 [file Data_Sheet_1.zip › confusion_matrices/gb/gb_subjectwise_confusion_matrices_row.png]

Binary • Baseline

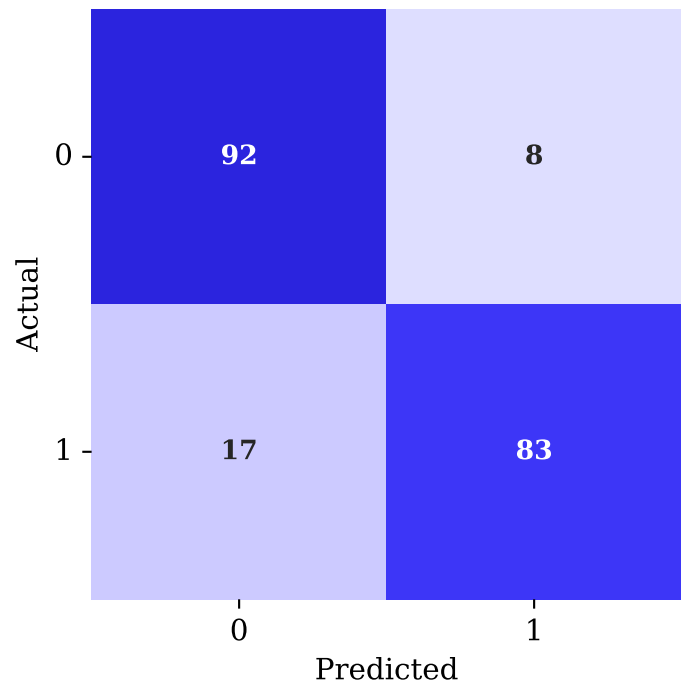

Binary • CRISP

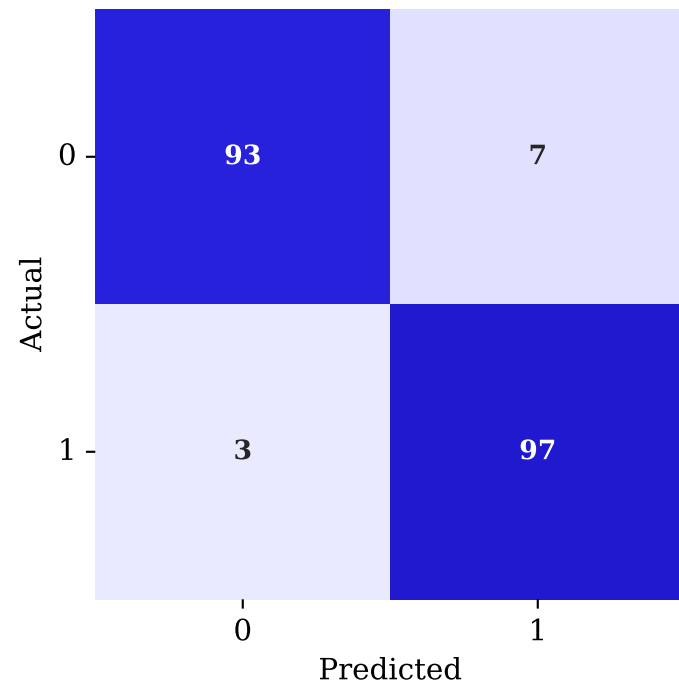

Multiclass • Baseline

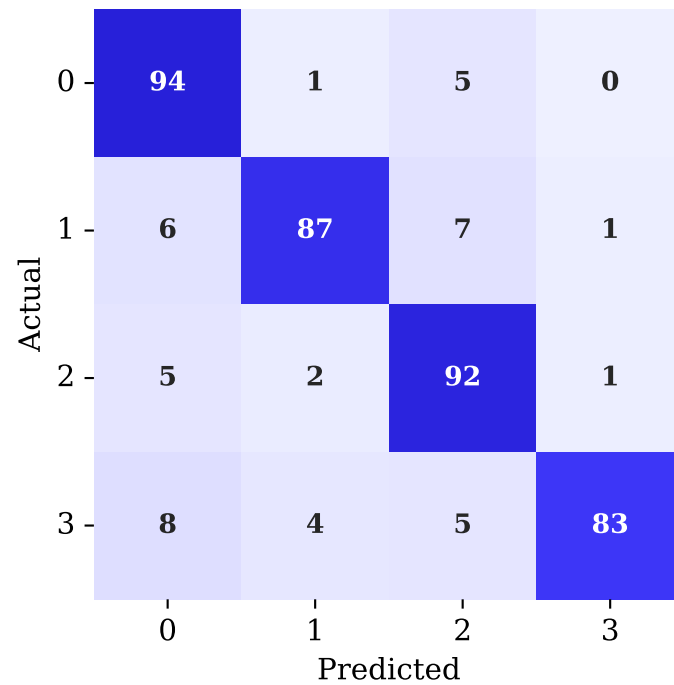

Multiclass • CRISP

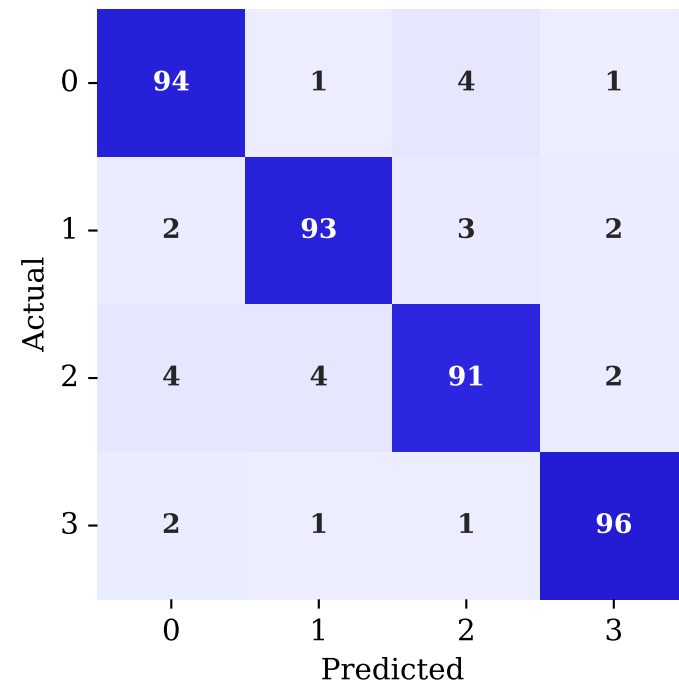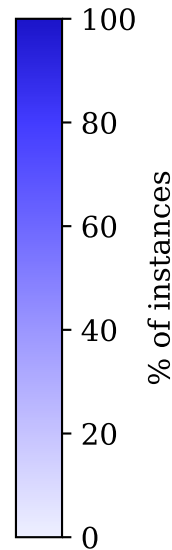

Supplement: Supplementary file 1 [file Data_Sheet_1.zip › confusion_matrices/knn/knn_overall_confusion_matrices_row.pdf]

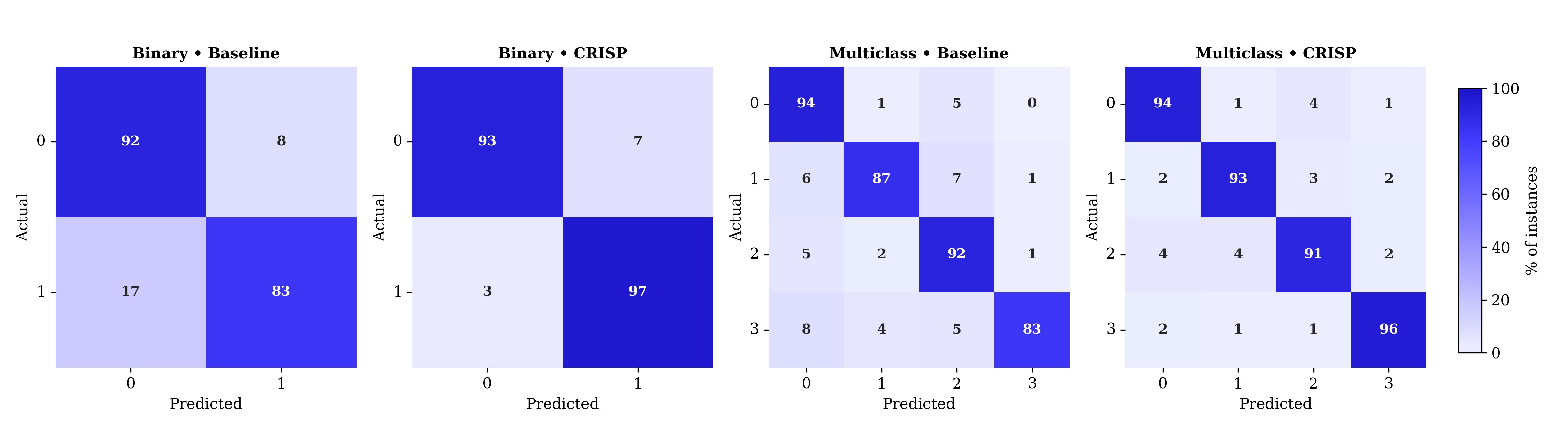

Supplement: Supplementary file 1 [file Data_Sheet_1.zip › confusion_matrices/knn/knn_overall_confusion_matrices_row.png]

Binary • Baseline

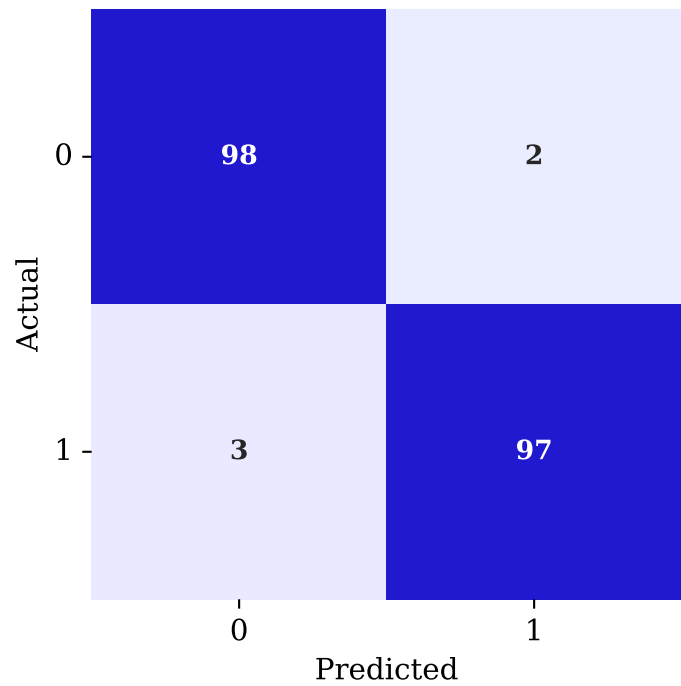

Binary • CRISP

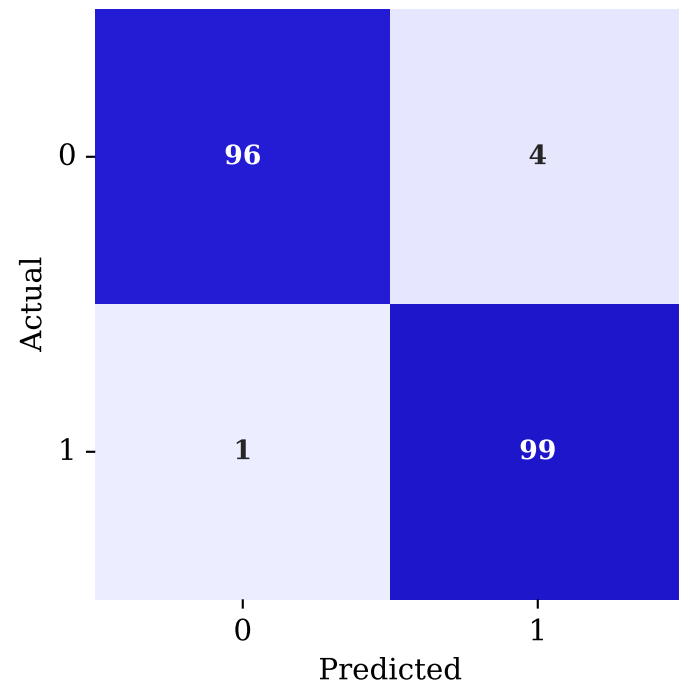

Multiclass • Baseline

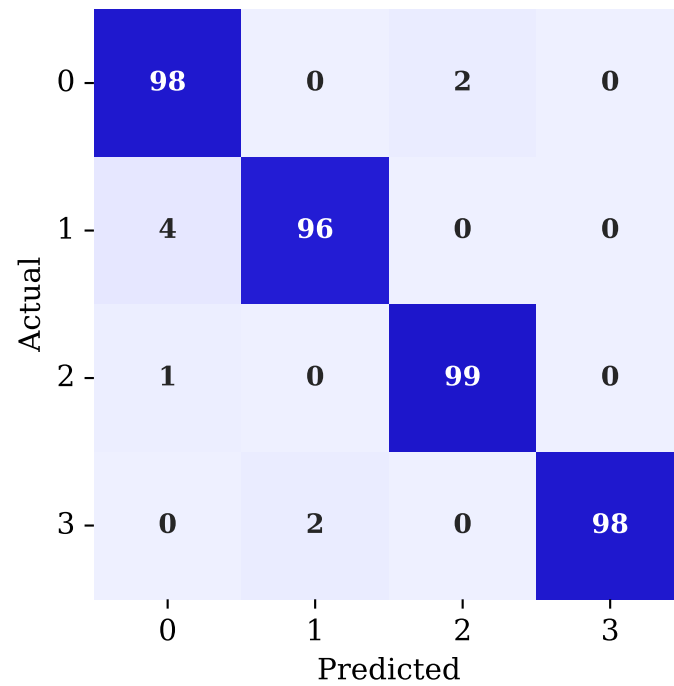

Multiclass • CRISP

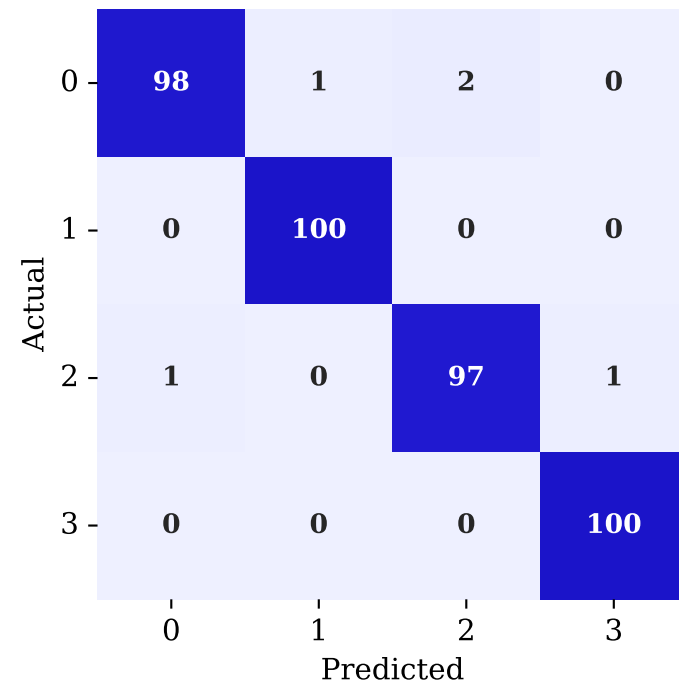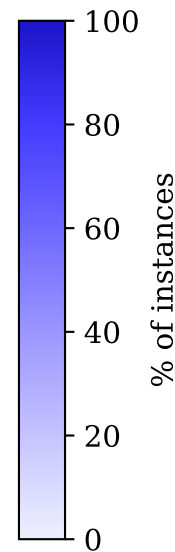

Supplement: Supplementary file 1 [file Data_Sheet_1.zip › confusion_matrices/knn/knn_subjectwise_confusion_matrices_row.pdf]

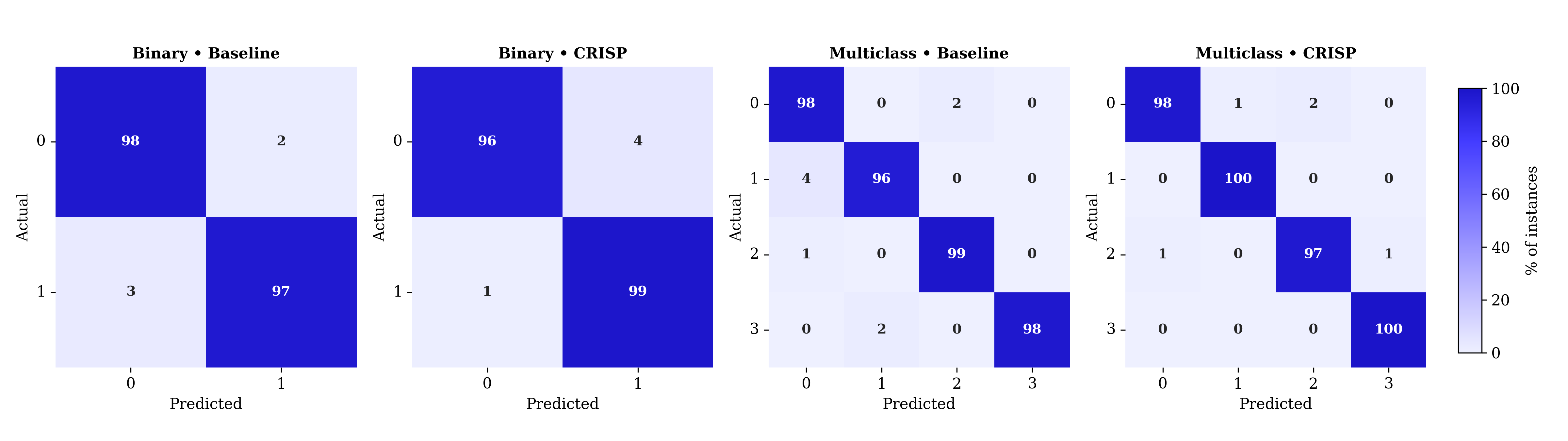

Supplement: Supplementary file 1 [file Data_Sheet_1.zip › confusion_matrices/knn/knn_subjectwise_confusion_matrices_row.png]

Binary • Baseline

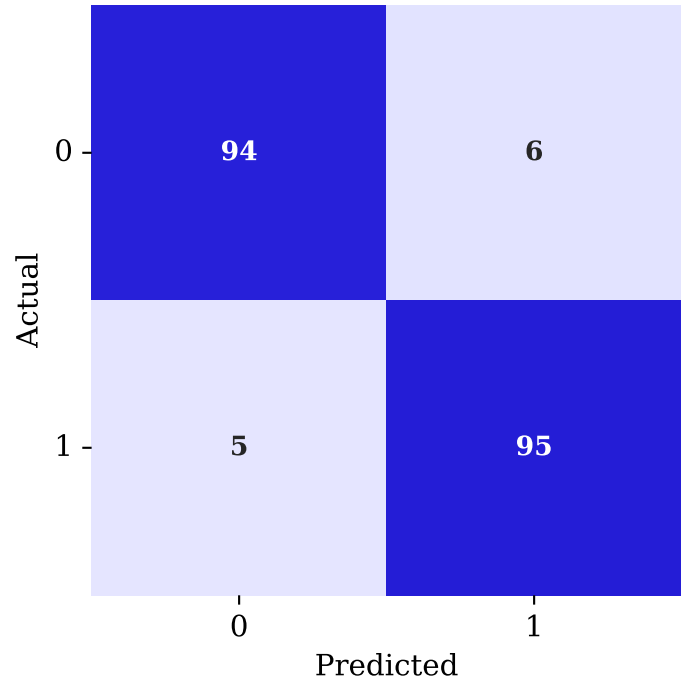

Binary • CRISP

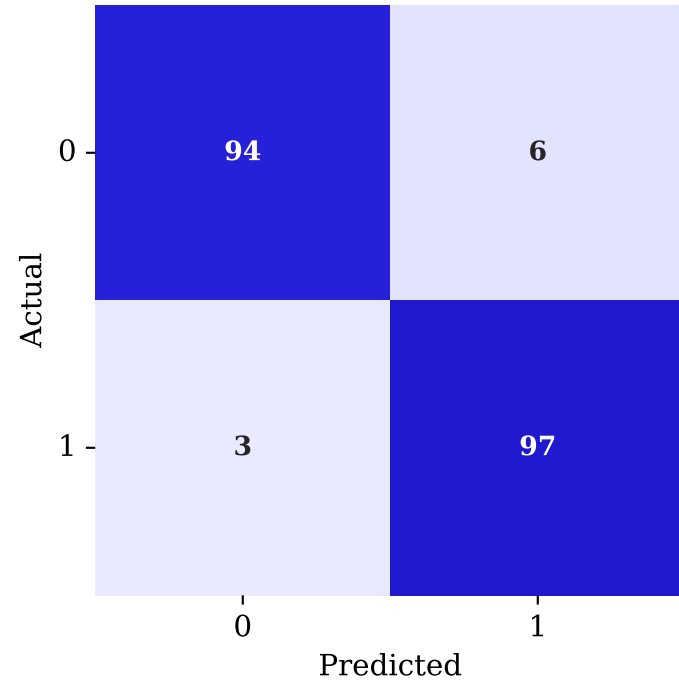

Multiclass • Baseline

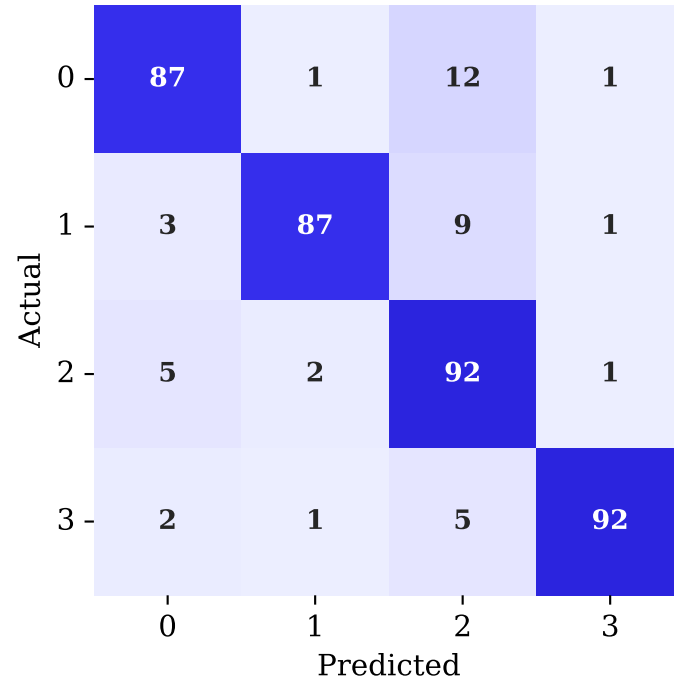

Multiclass • CRISP

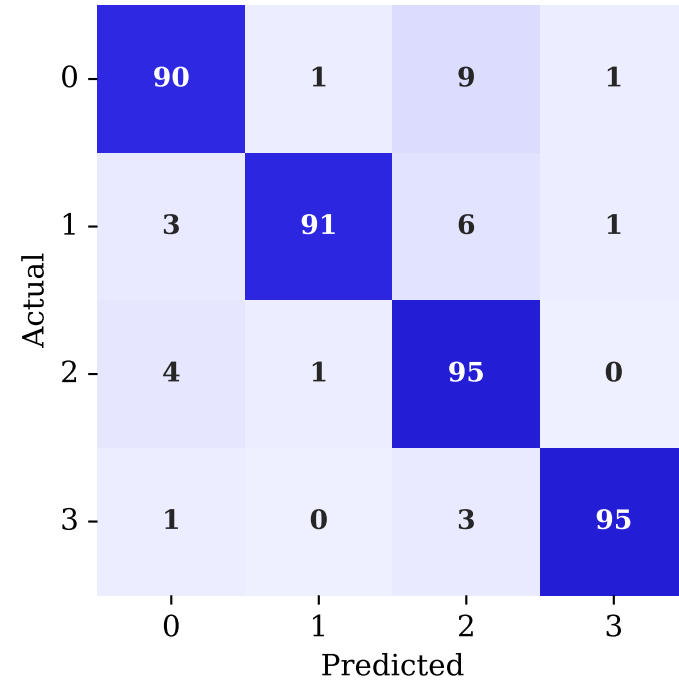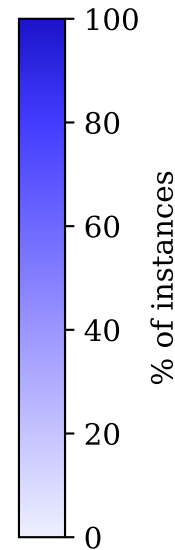

Supplement: Supplementary file 1 [file Data_Sheet_1.zip › confusion_matrices/rf/rf_overall_confusion_matrices_row.pdf]

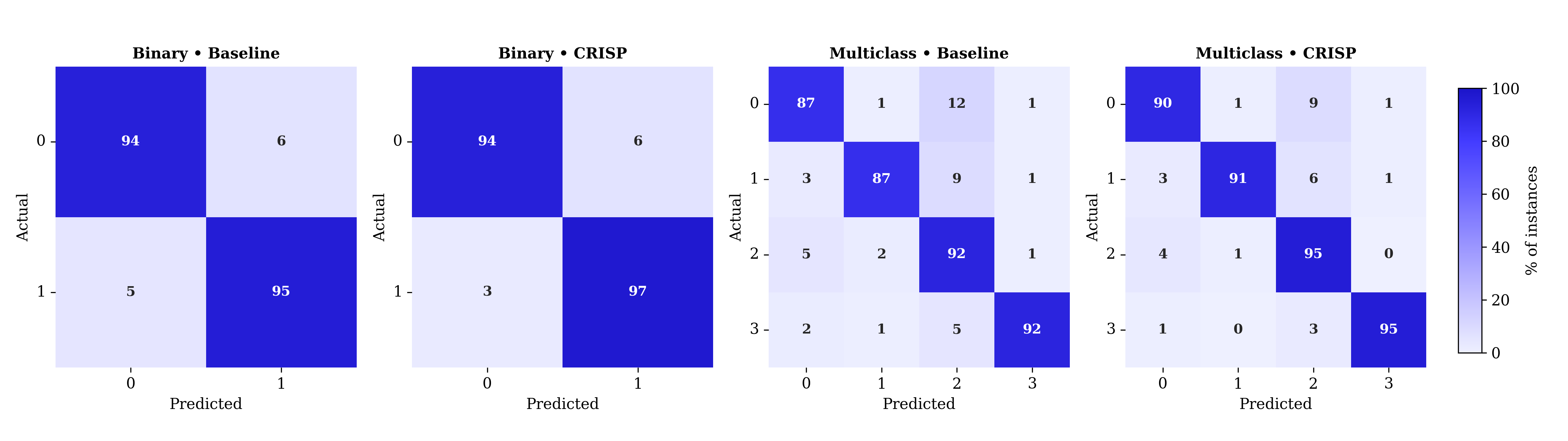

Supplement: Supplementary file 1 [file Data_Sheet_1.zip › confusion_matrices/rf/rf_overall_confusion_matrices_row.png]

Binary • Baseline

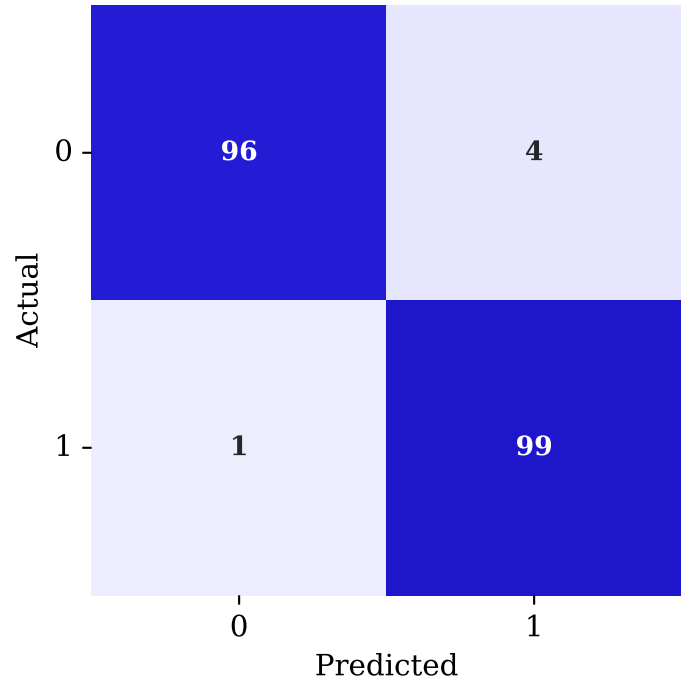

Binary • CRISP

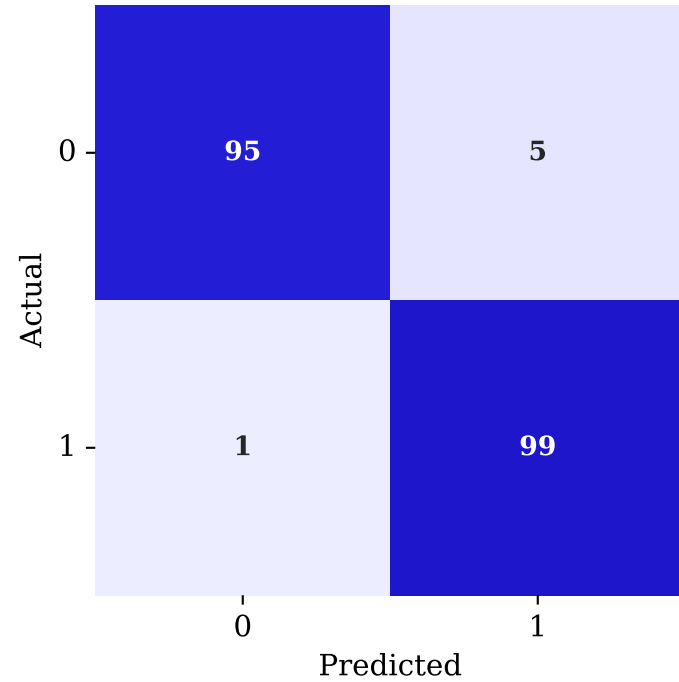

Multiclass • Baseline

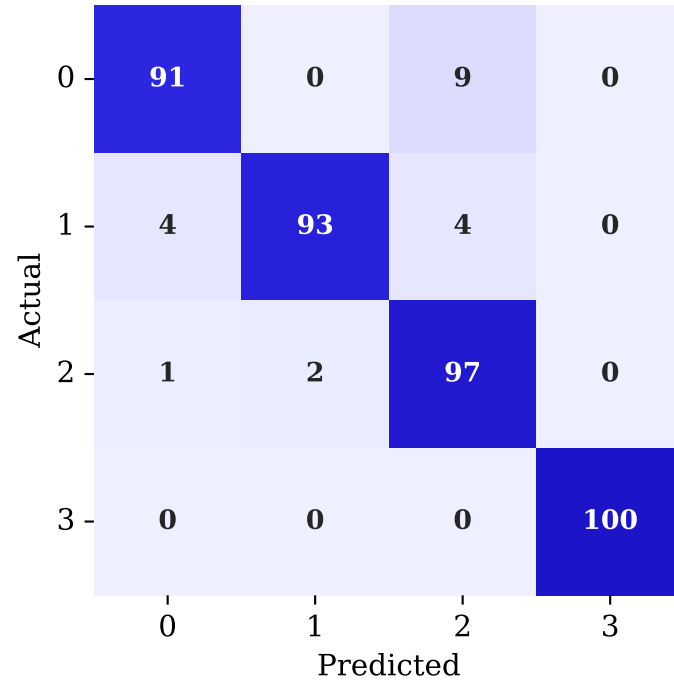

Multiclass • CRISP

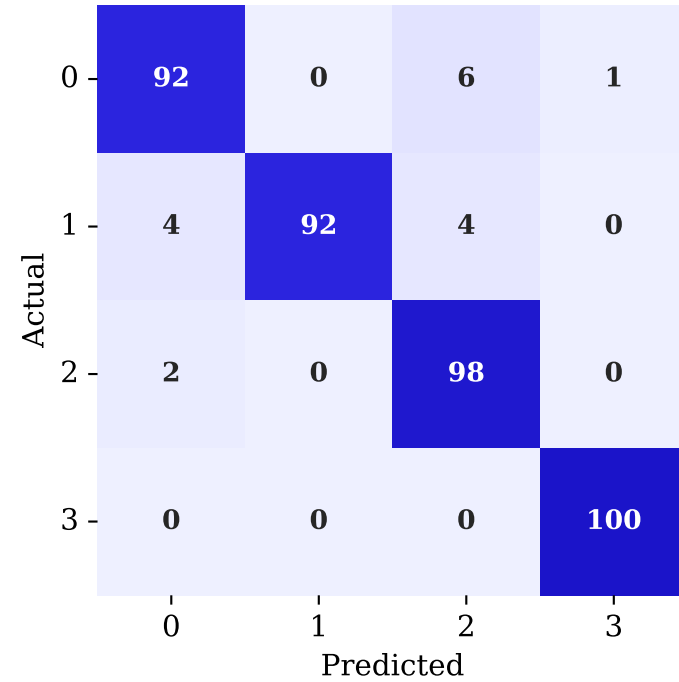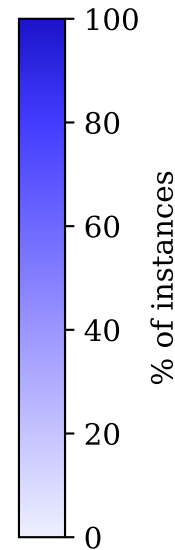

Supplement: Supplementary file 1 [file Data_Sheet_1.zip › confusion_matrices/rf/rf_subjectwise_confusion_matrices_row.pdf]

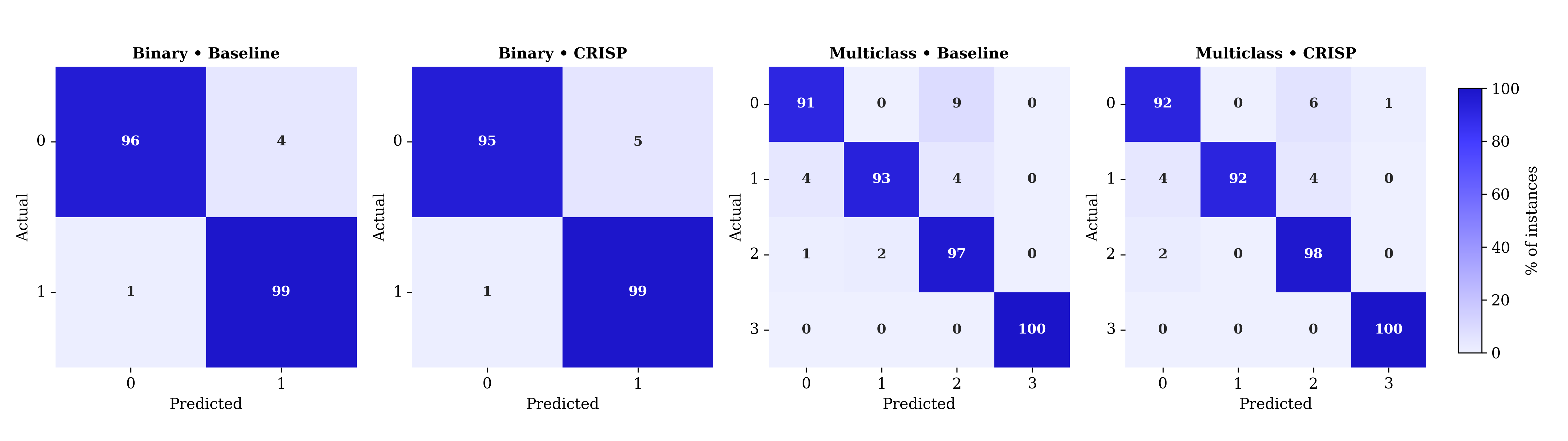

Supplement: Supplementary file 1 [file Data_Sheet_1.zip › confusion_matrices/rf/rf_subjectwise_confusion_matrices_row.png]

Binary • Baseline

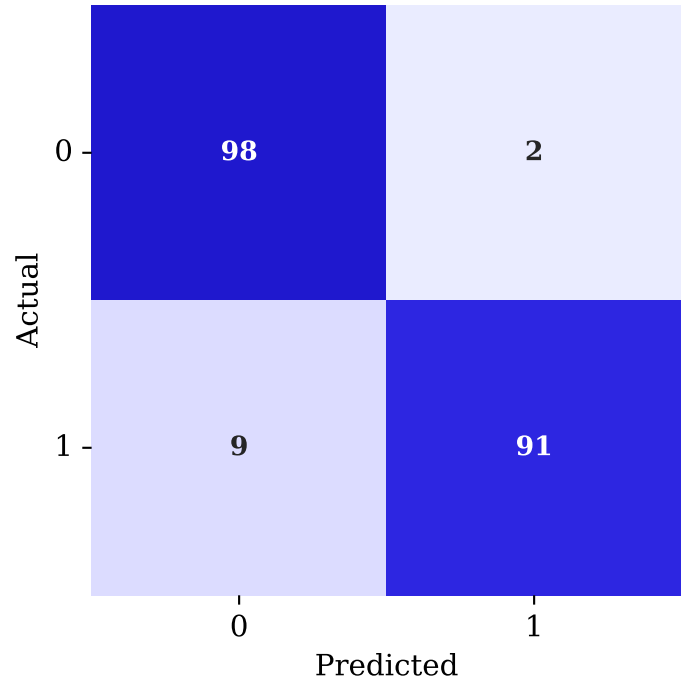

Binary • CRISP

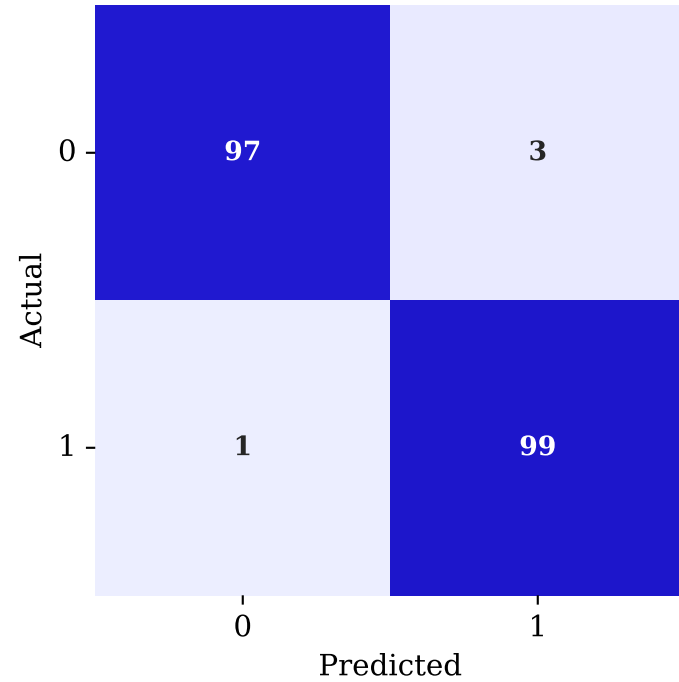

Multiclass • Baseline

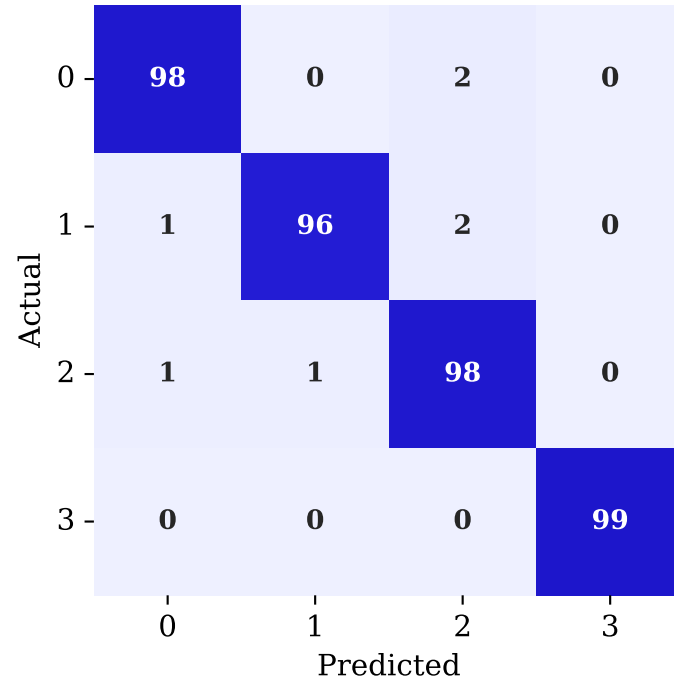

Multiclass • CRISP

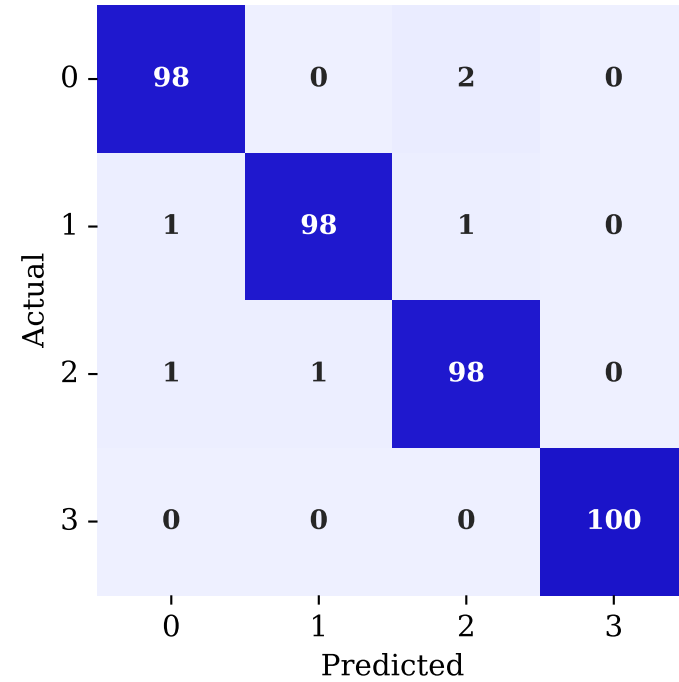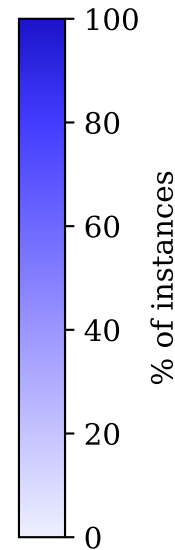

Supplement: Supplementary file 1 [file Data_Sheet_1.zip › confusion_matrices/xgb/xgb_overall_confusion_matrices_row.pdf]

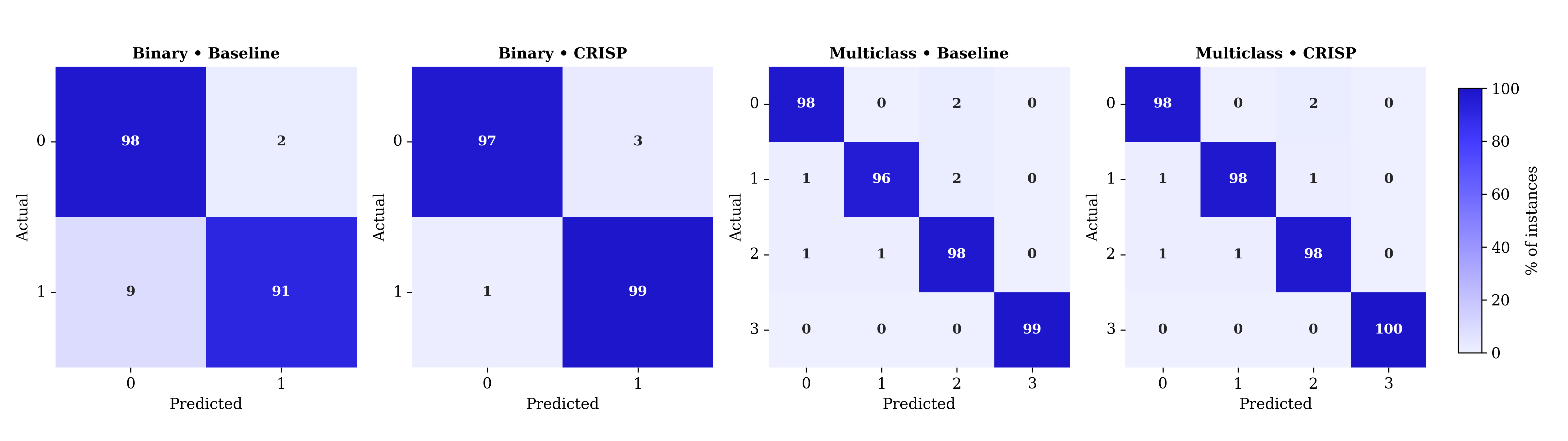

Supplement: Supplementary file 1 [file Data_Sheet_1.zip › confusion_matrices/xgb/xgb_overall_confusion_matrices_row.png]

Binary • Baseline

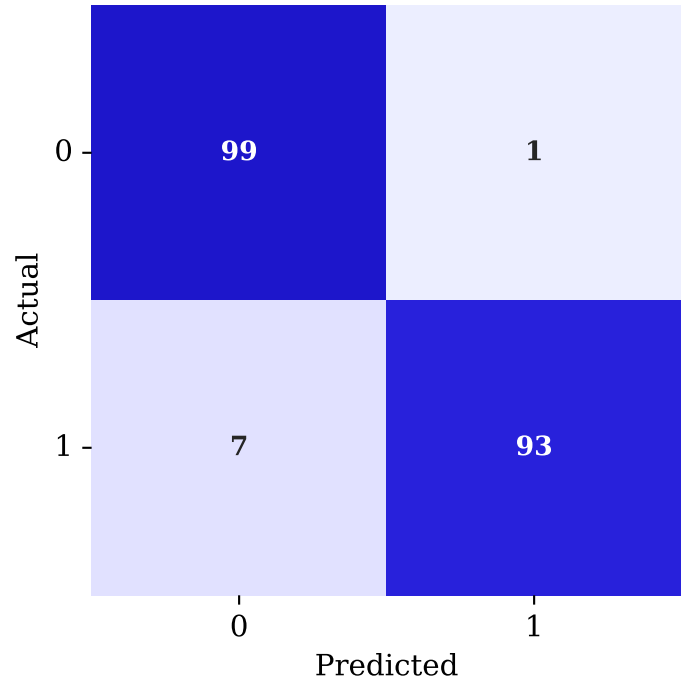

Binary • CRISP

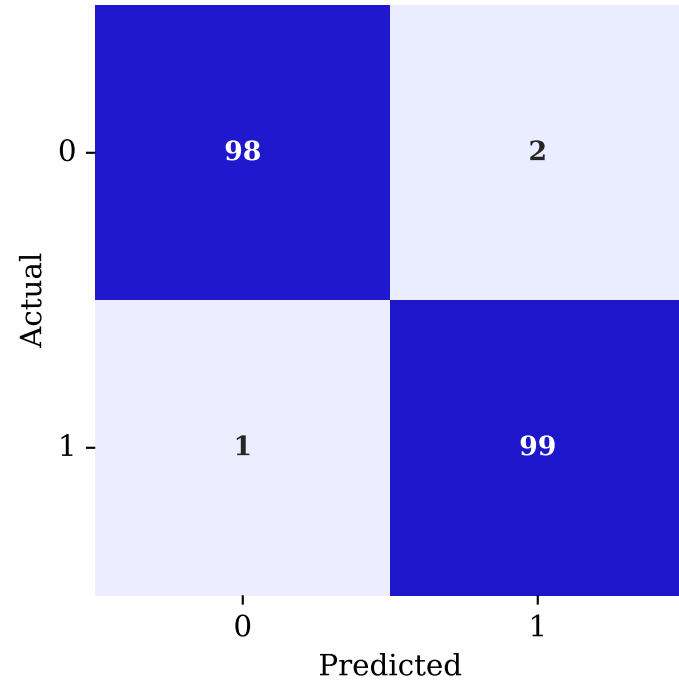

Multiclass • Baseline

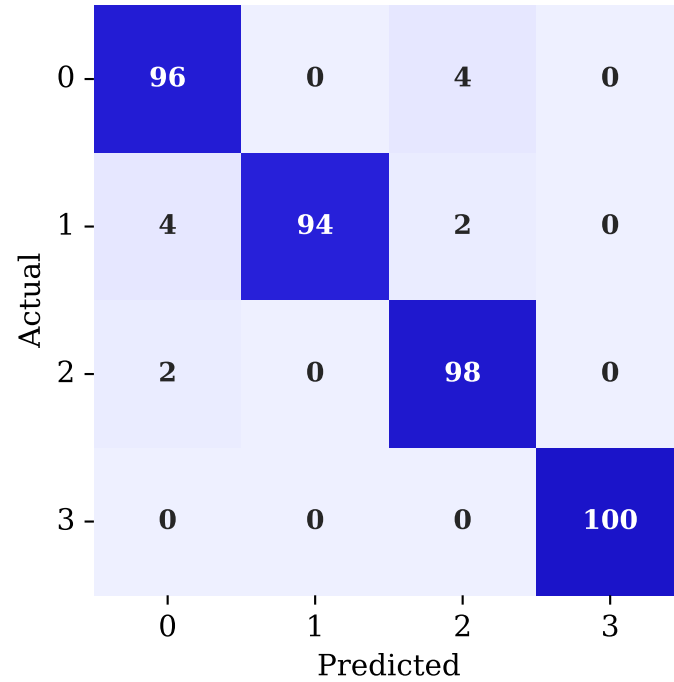

Multiclass • CRISP

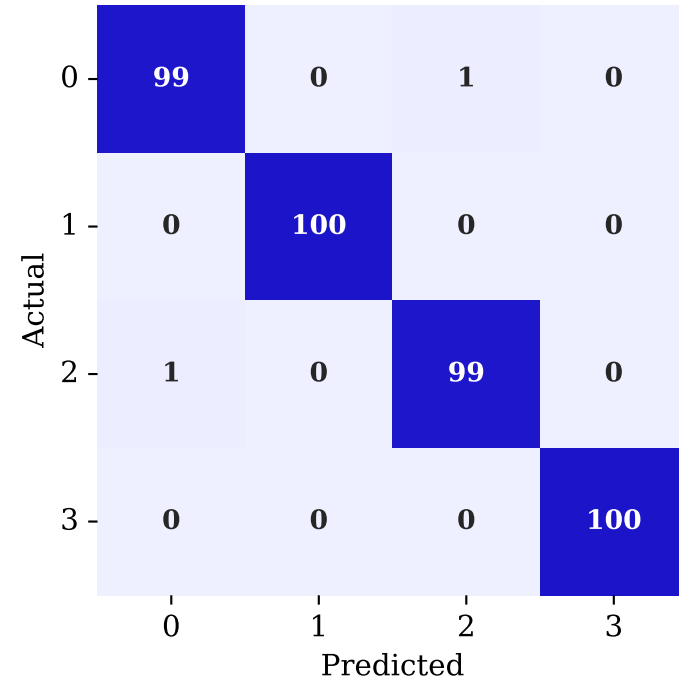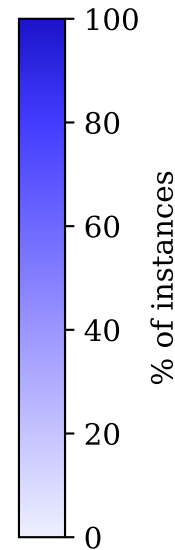

Supplement: Supplementary file 1 [file Data_Sheet_1.zip › confusion_matrices/xgb/xgb_subjectwise_confusion_matrices_row.pdf]

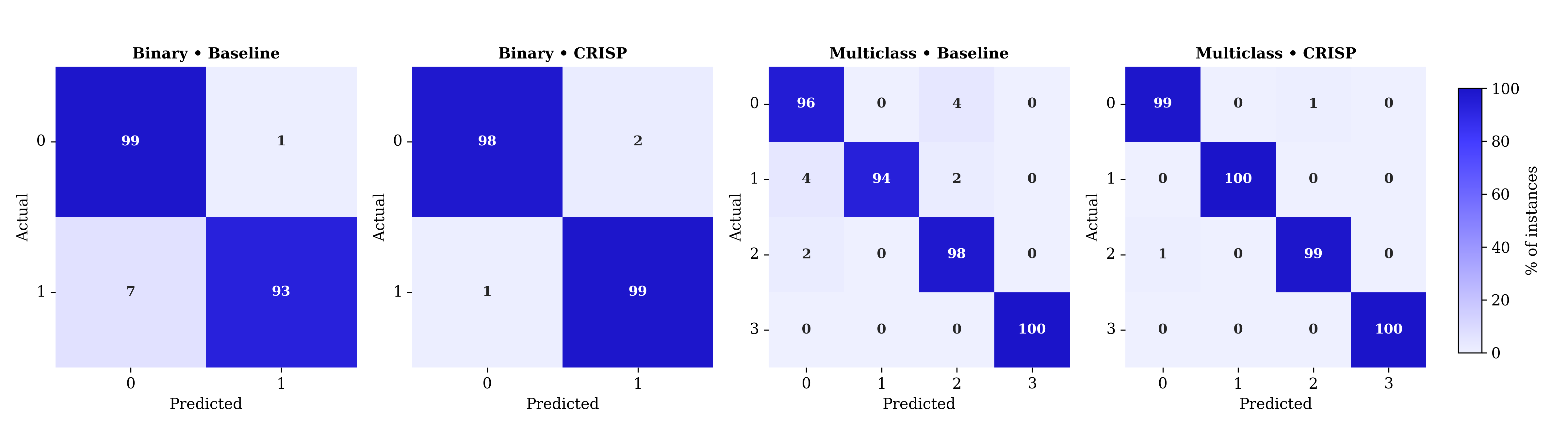

Supplement: Supplementary file 1 [file Data_Sheet_1.zip › confusion_matrices/xgb/xgb_subjectwise_confusion_matrices_row.png]
