# Supplementary material for: CRISP: a correlation-filtered recursive feature elimination and integration of SMOTE pipeline for gait-based Parkinson’s disease screening
Source: Front Comput Neurosci. 2025 Oct 10;19:1660963. doi: 10.3389/fncom.2025.1660963 (PMC12549659; doi:10.3389/fncom.2025.1660963)
Supplement: Supplementary file 2 [file Supplementary_file_1.docx]

**CRISP: A Correlation-filtered Recursive Feature Elimination & Integration of SMOTE Pipeline for Gait-Based Parkinson’s Disease Screening**

**Supplementary Material**

**S1. Binary PD Detection – Detailed Metrics**

**S1.1 Overall Protocol**

***Table S1.*** Performance of baseline classifiers over 5-fold CV (mean ± std) *before* applying our CRISP method (Correlation-filtered Recursive feature elimination & Integration of SMOTE)

| Models | Mean Accuracy | Mean Precision | Mean Recall | Mean Specificity | Mean F1-score |
| --- | --- | --- | --- | --- | --- |
| KNN | 0.890 ± 0.008 | 0.846 ± 0.015 | 0.834 ± 0.011 | 0.919 ± 0.009 | 0.840 ± 0.011 |
| DT | 0.919 ± 0.010 | 0.866 ± 0.019 | 0.907 ± 0.015 | 0.925 ± 0.012 | 0.886 ± 0.014 |
| RF | 0.943 ± 0.004 | 0.894 ± 0.009 | 0.949 ± 0.004 | 0.940 ± 0.005 | 0.921 ± 0.006 |
| GB | 0.963 ± 0.006 | 0.973 ± 0.005 | 0.918 ± 0.014 | 0.986 ± 0.002 | 0.945 ± 0.009 |
| XGB | 0.954 ± 0.004 | 0.956 ± 0.007 | 0.910 ± 0.009 | 0.978 ± 0.004 | 0.933 ± 0.006 |

**Table S2.** Performance of the same classifiers over 5-fold CV (mean ± std) *after* applying our CRISP method (Correlation-filtered Recursive feature elimination & Integration of SMOTE).

| Models | Mean Accuracy | Mean Precision | Mean Recall | Mean Specificity | Mean F1-score |
| --- | --- | --- | --- | --- | --- |
| KNN | 0.945 ± 0.003 | 0.880 ± 0.006 | 0.974 ± 0.005 | 0.929 ± 0.004 | 0.925 ± 0.004 |
| DT | 0.927 ± 0.013 | 0.849 ± 0.024 | 0.960 ± 0.019 | 0.909 ± 0.017 | 0.901 ± 0.017 |
| RF | 0.948 ± 0.004 | 0.891 ± 0.010 | 0.970 ± 0.003 | 0.937 ± 0.007 | 0.929 ± 0.004 |
| GB | 0.970 ± 0.005 | 0.931 ± 0.008 | 0.986 ± 0.008 | 0.961 ± 0.005 | 0.958 ± 0.007 |
| XGB | 0.977 ± 0.006 | 0.948 ± 0.015 | 0.988 ± 0.003 | 0.971 ± 0.009 | 0.968 ± 0.008 |

**S1.2 Subject-wise Protocol**

**Table S3.** Subject‐wise performance of baseline classifiers over 5-fold CV (mean ± std) *before* applying our CRISP method (Correlation-filtered Recursive feature elimination & Integration of SMOTE).

| Models | Mean Accuracy | Mean Precision | Mean Recall | Mean Specificity | Mean F1-score |
| --- | --- | --- | --- | --- | --- |
| KNN | 0.977 ± 0.010 | 0.980 ± 0.007 | 0.966 ± 0.019 | 0.985 ± 0.005 | 0.973 ± 0.012 |
| DT | 0.931 ± 0.019 | 0.902 ± 0.032 | 0.947 ± 0.021 | 0.919 ± 0.029 | 0.924 ± 0.021 |
| RF | 0.973 ± 0.005 | 0.947 ± 0.016 | 0.994 ± 0.011 | 0.956 ± 0.014 | 0.970 ± 0.005 |
| GB | 0.974 ± 0.008 | 0.994 ± 0.007 | 0.947 ± 0.019 | 0.996 ± 0.005 | 0.970 ± 0.010 |
| XGB | 0.961 ± 0.008 | 0.982 ± 0.011 | 0.927 ± 0.010 | 0.987 ± 0.008 | 0.954 ± 0.010 |

**Table S4.** Subject‐wise performance of the same classifiers over 5-fold CV (mean ± std) *after* applying our CRISP method (Correlation-filtered Recursive feature elimination & Integration of SMOTE).

| Models | Mean Accuracy | Mean Precision | Mean Recall | Mean Specificity | Mean F1-score |
| --- | --- | --- | --- | --- | --- |
| KNN | 0.974 ± 0.006 | 0.954 ± 0.011 | 0.989 ± 0.006 | 0.963 ± 0.009 | 0.971 ± 0.007 |
| DT | 0.921 ± 0.016 | 0.864 ± 0.028 | 0.975 ± 0.023 | 0.880 ± 0.030 | 0.916 ± 0.017 |
| RF | 0.967 ± 0.010 | 0.941 ± 0.018 | 0.986 ± 0.009 | 0.952 ± 0.015 | 0.963 ± 0.011 |
| GB | 0.972 ± 0.008 | 0.949 ± 0.014 | 0.989 ± 0.011 | 0.959 ± 0.013 | 0.968 ± 0.009 |
| XGB | 0.983 ± 0.008 | 0.973 ± 0.017 | 0.989 ± 0.006 | 0.978 ± 0.014 | 0.980 ± 0.009 |

**S2. Multiclass Severity Grading – Detailed Metrics**

**S2.1 Overall Protocol**

**Table S5.** Overall 5-Fold CV performance (mean ± std) of baseline multiclass classifiers for Parkinson’s Disease severity classification (Hoehn & Yahr scale) before applying our CRISP pipeline (Correlation-filtered RFE & Integration of SMOTE).

| Model | Mean Accuracy | Mean Precision | Mean Recall | Mean Specificity | Mean F1-Score |
| --- | --- | --- | --- | --- | --- |
| KNN | 0.909±0.005 | 0.909±0.005 | 0.909±0.005 | 0.966±0.002 | 0.908±0.005 |
| DT | 0.811±0.006 | 0.820±0.006 | 0.811±0.006 | 0.928±0.003 | 0.810±0.007 |
| RF | 0.892±0.008 | 0.895±0.006 | 0.892±0.008 | 0.959±0.003 | 0.892±0.008 |
| GB | 0.928±0.005 | 0.929±0.005 | 0.928±0.005 | 0.972±0.002 | 0.927±0.005 |
| XGB | 0.976±0.002 | 0.976±0.002 | 0.976±0.002 | 0.991±0.001 | 0.976±0.002 |

**Table S6.** Overall 5-Fold CV performance (mean ± std) of multiclass classifiers for Parkinson’s Disease severity classification (Hoehn & Yahr scale) after applying our CRISP pipeline.

| Model | Mean Accuracy | Mean Precision | Mean Recall | Mean Specificity | Mean F1-score |
| --- | --- | --- | --- | --- | --- |
| KNN | 0.930±0.004 | 0.931±0.004 | 0.930±0.004 | 0.973±0.001 | 0.930±0.004 |
| DT | 0.870±0.010 | 0.876±0.011 | 0.870±0.010 | 0.934±0.006 | 0.870±0.009 |
| RF | 0.923±0.008 | 0.925±0.008 | 0.923±0.008 | 0.961±0.005 | 0.923±0.008 |
| GB | 0.936±0.002 | 0.936±0.002 | 0.936±0.002 | 0.970±0.002 | 0.936±0.002 |
| XGB | 0.981±0.002 | 0.981±0.002 | 0.981±0.002 | 0.991±0.001 | 0.981±0.002 |

**S2.2 Subject-wise Protocol**

**Table S7.** Subject-wise evaluation metrics (mean ± std) of baseline multiclass classifiers for Parkinson’s Disease severity classification (Hoehn & Yahr scale) before applying our CRISP pipeline.

| Model | Mean Accuracy | Mean Precision | Mean Recall | Mean Specificity | Mean F1-Score |
| --- | --- | --- | --- | --- | --- |
| KNN | 0.979±0.005 | 0.979±0.005 | 0.979±0.005 | 0.992±0.002 | 0.979±0.005 |
| DT | 0.850±0.017 | 0.862±0.018 | 0.850±0.017 | 0.941±0.007 | 0.849±0.018 |
| RF | 0.936±0.008 | 0.940±0.007 | 0.936±0.008 | 0.976±0.003 | 0.936±0.008 |
| GB | 0.955±0.016 | 0.956±0.016 | 0.955±0.016 | 0.981±0.006 | 0.954±0.016 |
| XGB | 0.962±0.007 | 0.963±0.007 | 0.962±0.007 | 0.985±0.003 | 0.962±0.007 |

**Table S8.** Subject-wise evaluation metrics (mean ± std) of multiclass classifiers for Parkinson’s Disease severity classification (Hoehn & Yahr scale) after applying our CRISP pipeline.

| Model | Mean Accuracy | Mean Precision | Mean Recall | Mean Specificity | Mean F1-Score |
| --- | --- | --- | --- | --- | --- |
| KNN | 0.982±0.005 | 0.982±0.005 | 0.982±0.005 | 0.994±0.002 | 0.982±0.005 |
| DT | 0.865±0.005 | 0.872±0.006 | 0.865±0.005 | 0.949±0.003 | 0.865±0.005 |
| RF | 0.946±0.005 | 0.948±0.004 | 0.946±0.005 | 0.979±0.001 | 0.946±0.005 |
| GB | 0.961±0.008 | 0.961±0.008 | 0.961±0.008 | 0.985±0.003 | 0.961±0.008 |
| XGB | 0.993±0.005 | 0.993±0.005 | 0.993±0.005 | 0.997±0.002 | 0.993±0.005 |

**Table S9.** Ablation analysis on Gradient Boosting (GB) and XGBoost (XGB) models showing overall accuracy and F1-score with and without key CRISP component (Overall)

| Model | Configuration | Accuracy | F1 Score |
| --- | --- | --- | --- |
| GB | CRISP | 97.0 ± 0.5 | 95.8 ± 0.7 |
| GB | w/o RFE | 95.3 ± 0.7 | 93.1 ± 0.9 |
| GB | w/o SMOTE | 95.9 ± 0.6 | 94.0 ± 0.8 |
| XGB | CRISP | 97.7 ± 0.6 | 96.8 ± 0.8 |
| XGB | w/o RFE | 96.0 ± 0.6 | 94.1 ± 1.0 |
| XGB | w/o SMOTE | 96.5 ± 0.5 | 94.9 ± 0.8 |

| \| \| **Table S10.** Paired t-test results for binary classification (PD detection): This table compares CRISP-enhanced models against their respective baselines using paired t-tests across Accuracy, Precision, Recall, Specificity, and F1-score. CRISP demonstrates significant performance gains in most cases, particularly in Accuracy, Recall, and F1-score.   \| **Model** \| **Metric** \| **T-stat** \| **p-value** \| **Significant (p < 0.05)** \| \| --- \| --- \| --- \| --- \| --- \| \| KNN \| Accuracy \| 6.608 \| 0.0027 \| ✓ \| \| KNN \| Precision \| 7.028 \| 0.0022 \| ✓ \| \| KNN \| Recall \| 6.608 \| 0.0027 \| ✓ \| \| KNN \| Specificity \| 5.375 \| 0.0058 \| ✓ \| \| KNN \| F1 \| 6.707 \| 0.0026 \| ✓ \| \| DT \| Accuracy \| 23.541 \| 0.0002 \| ✓ \| \| DT \| Precision \| 21.83 \| 0.0001 \| ✓ \| \| DT \| Recall \| 23.541 \| 0.0004 \| ✓ \| \| DT \| Specificity \| 2.438 \| 0.0713 \| ✗ \| \| DT \| F1 \| 25.563 \| 0.0004 \| ✓ \| \| RF \| Accuracy \| 11.739 \| 0.0003 \| ✓ \| \| RF \| Precision \| 10.972 \| 0.0004 \| ✓ \| \| RF \| Recall \| 11.739 \| 0.0003 \| ✓ \| \| RF \| Specificity \| 1.126 \| 0.3231 \| ✗ \| \| RF \| F1 \| 11.698 \| 0.0003 \| ✓ \| \| GB \| Accuracy \| 3.01 \| 0.0396 \| ✓ \| \| GB \| Precision \| 2.685 \| 0.0549 \| ✗ \| \| GB \| Recall \| 3.01 \| 0.0396 \| ✓ \| \| GB \| Specificity \| -1.356 \| 0.2467 \| ✗ \| \| GB \| F1 \| 3.048 \| 0.0381 \| ✓ \| \| XGB \| Accuracy \| 4.289 \| 0.0128 \| ✓ \| \| XGB \| Precision \| 4.253 \| 0.0131 \| ✓ \| \| XGB \| Recall \| 4.289 \| 0.0128 \| ✓ \| \| XGB \| Specificity \| 0.402 \| 0.7085 \| ✗ \| \| XGB \| F1 \| 4.314 \| 0.0125 \| ✓ \| \| \| --- \| --- \| --- \| --- \| --- \| --- \| --- \| --- \| --- \| --- \| --- \| --- \| --- \| --- \| --- \| --- \| --- \| --- \| --- \| --- \| --- \| --- \| --- \| --- \| --- \| --- \| --- \| --- \| --- \| --- \| --- \| --- \| --- \| --- \| --- \| --- \| --- \| --- \| --- \| --- \| --- \| --- \| --- \| --- \| --- \| --- \| --- \| --- \| --- \| --- \| --- \| --- \| --- \| --- \| --- \| --- \| --- \| --- \| --- \| --- \| --- \| --- \| --- \| --- \| --- \| --- \| --- \| --- \| --- \| --- \| --- \| --- \| --- \| --- \| --- \| --- \| --- \| --- \| --- \| --- \| --- \| --- \| --- \| --- \| --- \| --- \| --- \| --- \| --- \| --- \| --- \| --- \| --- \| --- \| --- \| --- \| --- \| --- \| --- \| --- \| --- \| --- \| --- \| --- \| --- \| --- \| --- \| --- \| --- \| --- \| --- \| --- \| --- \| --- \| --- \| --- \| --- \| --- \| --- \| --- \| --- \| --- \| --- \| --- \| --- \| --- \| --- \| --- \| --- \| --- \| --- \|   **Table S11.** Paired t-test results for multiclass classification (PD severity grading): This table summarizes statistical comparisons between CRISP-enhanced and baseline models across multiclass classification metrics. Most improvements, especially in Recall and F1-score, are statistically significant, highlighting CRISP's robustness across severity levels.   \| Model \| Metric \| T-stat \| p-value \| Significant (p < 0.05) \| \| --- \| --- \| --- \| --- \| --- \| \| KNN \| Accuracy \| 6.608 \| 0.0027 \| ✓ \| \| KNN \| Precision \| 7.028 \| 0.0022 \| ✓ \| \| KNN \| Recall \| 6.608 \| 0.0027 \| ✓ \| \| KNN \| Specificity \| 5.375 \| 0.0058 \| ✓ \| \| KNN \| F1 \| 6.707 \| 0.0026 \| ✓ \| \| DT \| Accuracy \| 23.541 \| 0.0002 \| ✓ \| \| DT \| Precision \| 21.131 \| 0.0004 \| ✓ \| \| DT \| Recall \| 23.541 \| 0.0002 \| ✓ \| \| DT \| Specificity \| 2.438 \| 0.0713 \| ✗ \| \| DT \| F1 \| 25.563 \| 0.0001 \| ✓ \| \| RF \| Accuracy \| 11.739 \| 0.0003 \| ✓ \| \| RF \| Precision \| 10.972 \| 0.0004 \| ✓ \| \| RF \| Recall \| 11.739 \| 0.0003 \| ✓ \| \| RF \| Specificity \| 1.126 \| 0.3231 \| ✗ \| \| RF \| F1 \| 11.698 \| 0.0003 \| ✓ \| \| GB \| Accuracy \| 3.01 \| 0.0396 \| ✓ \| \| GB \| Precision \| 2.685 \| 0.0549 \| ✗ \| \| GB \| Recall \| 3.01 \| 0.0396 \| ✓ \| \| GB \| Specificity \| -1.356 \| 0.2467 \| ✗ \| \| GB \| F1 \| 3.048 \| 0.0381 \| ✓ \| \| XGB \| Accuracy \| 4.289 \| 0.0128 \| ✓ \| \| XGB \| Precision \| 4.253 \| 0.0131 \| ✓ \| \| XGB \| Recall \| 4.289 \| 0.0128 \| ✓ \| \| XGB \| Specificity \| 0.402 \| 0.7085 \| ✗ \| \| XGB \| F1 \| 4.314 \| 0.0125 \| ✓ \| \| \| --- \| --- \| --- \| --- \| --- \| --- \| --- \| --- \| --- \| --- \| --- \| --- \| --- \| --- \| --- \| --- \| --- \| --- \| --- \| --- \| --- \| --- \| --- \| --- \| --- \| --- \| --- \| --- \| --- \| --- \| --- \| --- \| --- \| --- \| --- \| --- \| --- \| --- \| --- \| --- \| --- \| --- \| --- \| --- \| --- \| --- \| --- \| --- \| --- \| --- \| --- \| --- \| --- \| --- \| --- \| --- \| --- \| --- \| --- \| --- \| --- \| --- \| --- \| --- \| --- \| --- \| --- \| --- \| --- \| --- \| --- \| --- \| --- \| --- \| --- \| --- \| --- \| --- \| --- \| --- \| --- \| --- \| --- \| --- \| --- \| --- \| --- \| --- \| --- \| --- \| --- \| --- \| --- \| --- \| --- \| --- \| --- \| --- \| --- \| --- \| --- \| --- \| --- \| --- \| --- \| --- \| --- \| --- \| --- \| --- \| --- \| --- \| --- \| --- \| --- \| --- \| --- \| --- \| --- \| --- \| --- \| --- \| --- \| --- \| --- \| --- \| --- \| --- \| --- \| --- \| --- \| --- \| --- \| --- \| --- \| --- \| --- \| --- \| --- \| --- \| --- \| --- \| --- \| --- \| --- \| --- \| --- \| --- \| --- \| --- \| --- \| --- \| --- \| --- \| --- \| --- \| --- \| --- \| --- \| --- \| --- \| --- \| --- \| --- \| --- \| --- \| --- \| --- \| --- \| --- \| --- \| --- \| --- \| --- \| --- \| --- \| --- \| --- \| --- \| --- \| --- \| --- \| --- \| --- \| --- \| --- \| --- \| --- \| --- \| --- \| --- \| --- \| --- \| --- \| --- \| --- \| --- \| --- \| --- \| --- \| --- \| --- \| --- \| --- \| --- \| --- \| --- \| --- \| --- \| --- \| --- \| --- \| --- \| --- \| --- \| --- \| --- \| --- \| --- \| --- \| --- \| --- \| --- \| --- \| --- \| --- \| --- \| --- \| --- \| --- \| --- \| --- \| --- \| --- \| --- \| --- \| --- \| --- \| --- \| --- \| --- \| --- \| --- \| --- \| --- \| --- \| --- \| --- \| --- \| --- \| --- \| --- \| --- \| --- \| --- \| --- \| --- \| --- \| --- \| --- \| --- \| --- \| \|  \| \|  \| |
| --- | --- | --- | --- | --- | --- | --- | --- | --- | --- | --- | --- | --- | --- | --- | --- | --- | --- | --- | --- | --- | --- | --- | --- | --- | --- | --- | --- | --- | --- | --- | --- | --- | --- | --- | --- | --- | --- | --- | --- | --- | --- | --- | --- | --- | --- | --- | --- | --- | --- | --- | --- | --- | --- | --- | --- | --- | --- | --- | --- | --- | --- | --- | --- | --- | --- | --- | --- | --- | --- | --- | --- | --- | --- | --- | --- | --- | --- | --- | --- | --- | --- | --- | --- | --- | --- | --- | --- | --- | --- | --- | --- | --- | --- | --- | --- | --- | --- | --- | --- | --- | --- | --- | --- | --- | --- | --- | --- | --- | --- | --- | --- | --- | --- | --- | --- | --- | --- | --- | --- | --- | --- | --- | --- | --- | --- | --- | --- | --- | --- | --- | --- | --- | --- | --- | --- | --- | --- | --- | --- | --- | --- | --- | --- | --- | --- | --- | --- | --- | --- | --- | --- | --- | --- | --- | --- | --- | --- | --- | --- | --- | --- | --- | --- | --- | --- | --- | --- | --- | --- | --- | --- | --- | --- | --- | --- | --- | --- | --- | --- | --- | --- | --- | --- | --- | --- | --- | --- | --- | --- | --- | --- | --- | --- | --- | --- | --- | --- | --- | --- | --- | --- | --- | --- | --- | --- | --- | --- | --- | --- | --- | --- | --- | --- | --- | --- | --- | --- | --- | --- | --- | --- | --- | --- | --- | --- | --- | --- | --- | --- | --- | --- | --- | --- | --- | --- | --- | --- | --- | --- | --- | --- | --- | --- | --- | --- | --- | --- | --- | --- | --- | --- | --- | --- | --- | --- | --- | --- | --- | --- | --- | --- | --- | --- | --- |
|  |
|  |
|  |
|  |
|  |
|  |
|  |
|  |
|  |
|  |
|  |

**S3. Confusion Matrix Visualizations**


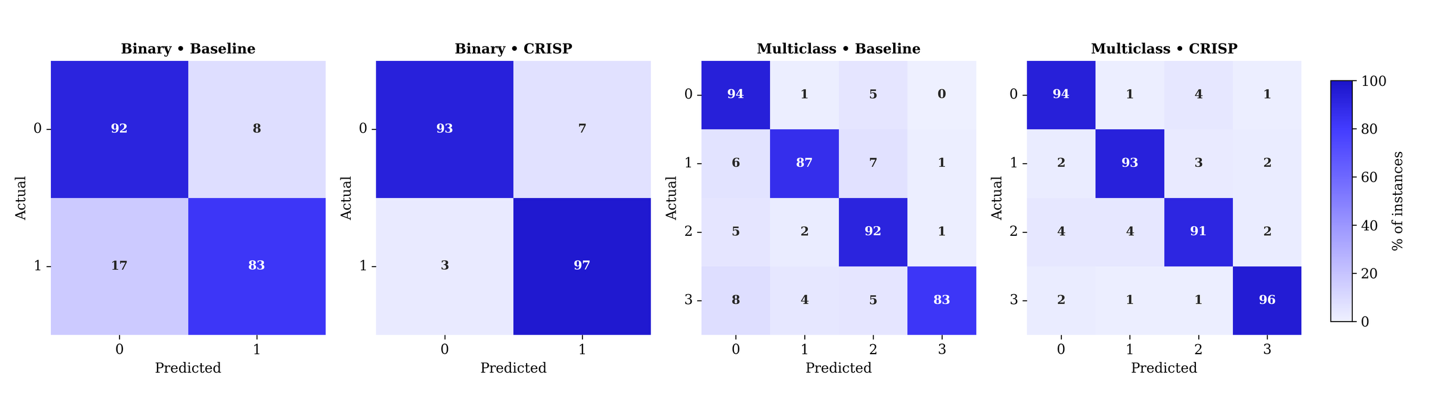


Figure S2. Confusion matrices (percentage view) for the K-Nearest Neighbors (KNN) model under the overall evaluation protocol. Panels are ordered left → right: (1) Binary baseline, (2) Binary with CRISP, (3) Multiclass baseline, (4) Multiclass with CRISP (Hoehn–Yahr grades 0–3). CRISP sharply reduces binary false-negatives from 17% to 3%. In the multiclass case, class separation improves across all grades, especially grades 1–2 and 2–3. Axes indicate true (rows) and predicted (columns) labels.


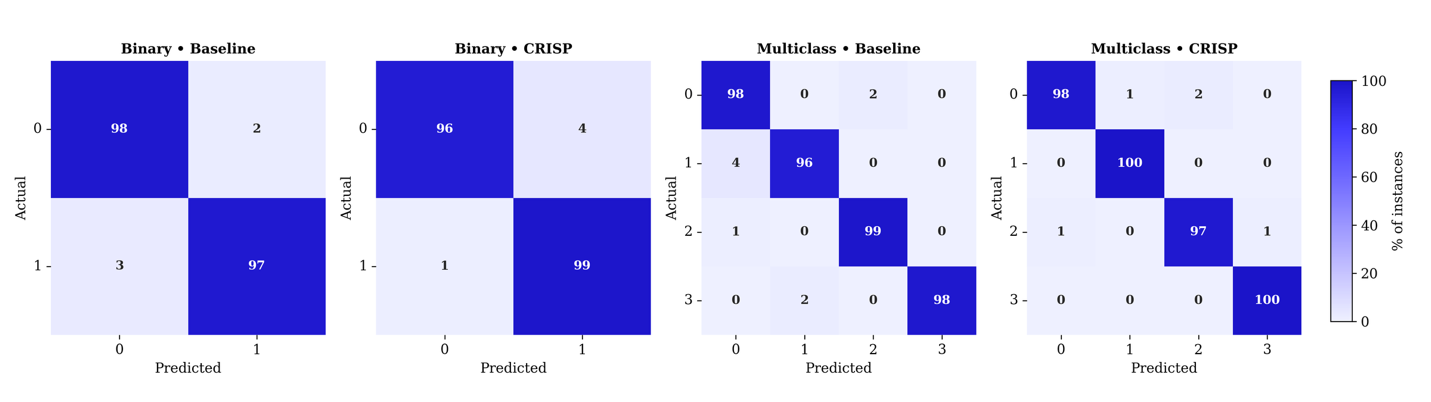


Figure S3. Confusion matrices (percentage view) for the K-Nearest Neighbors (KNN) model under the subject-wise evaluation protocol. Panels are ordered left to right: (1) Binary baseline, (2) Binary with CRISP, (3) Multiclass baseline, (4) Multiclass with CRISP. Hoehn and Yahr grades 0 to 3 are used for severity levels. CRISP reduces binary false-negatives from 3% to 1%, and improves multiclass predictions across all grades, achieving 100% accuracy for both grades 1 and 3. Axes denote true (rows) and predicted (columns) classes.


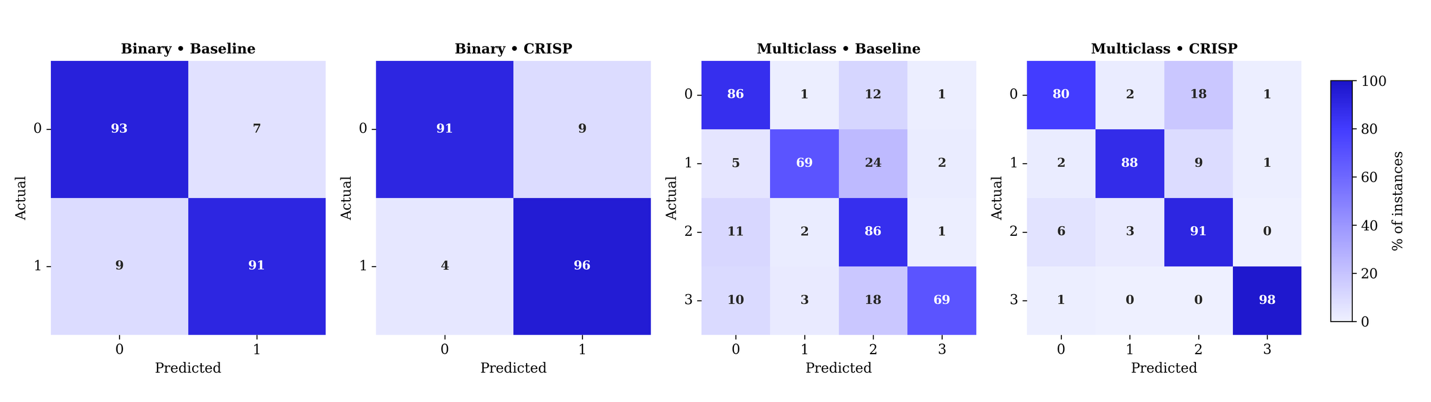


Figure S4. Confusion matrices (percentage view) for the Decision Tree (DT) model under the Overall evaluation protocol. Panels are ordered left → right: (1) Binary baseline, (2) Binary after CRISP, (3) Multiclass baseline, and (4) Multiclass after CRISP (Hoehn–Yahr grades 0–3). Violet-to-indigo shading reflects the proportion of instances in each cell (darker = higher percentage; shared 0–100% colour bar at far right). CRISP improves class separation: binary false-negatives are reduced from 9% to 4%, and multiclass misclassifications between adjacent grades (e.g., 1 ↔ 2 and 2 ↔ 3) are notably compressed. Axes indicate true labels (rows) and predicted labels (columns).


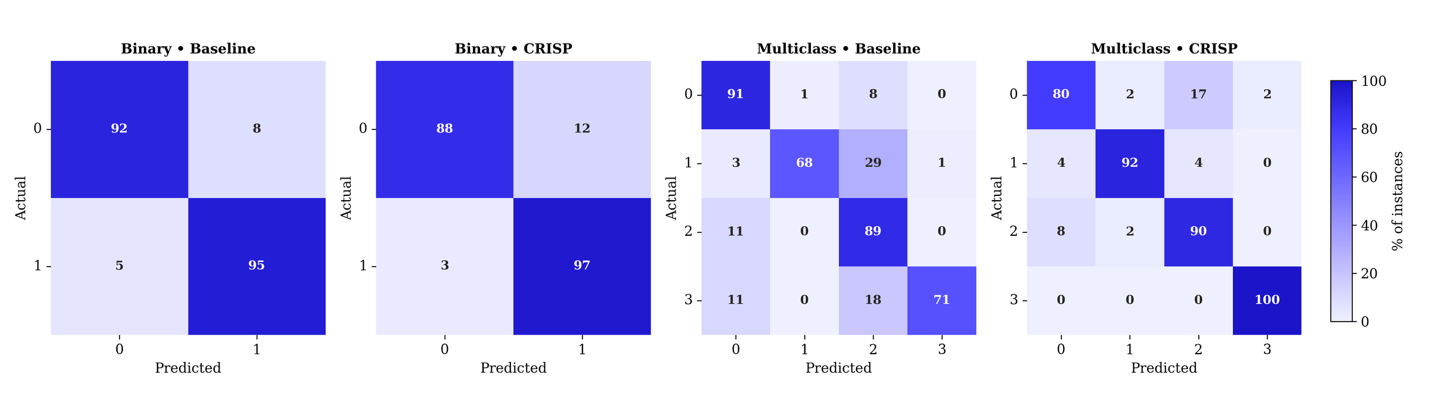


Figure S5. Confusion matrices (percentage view) for the Decision Tree (DT) model under the subject-wise evaluation protocol. Panels are ordered left → right: (1) Binary baseline, (2) Binary with CRISP, (3) Multiclass baseline, (4) Multiclass with CRISP (Hoehn–Yahr grades 0–3). CRISP reduces binary false-negatives from 5% to 3% and improves multiclass separation, especially between grades 1 and 2. Axis labels indicate true (rows) and predicted (columns) classes.


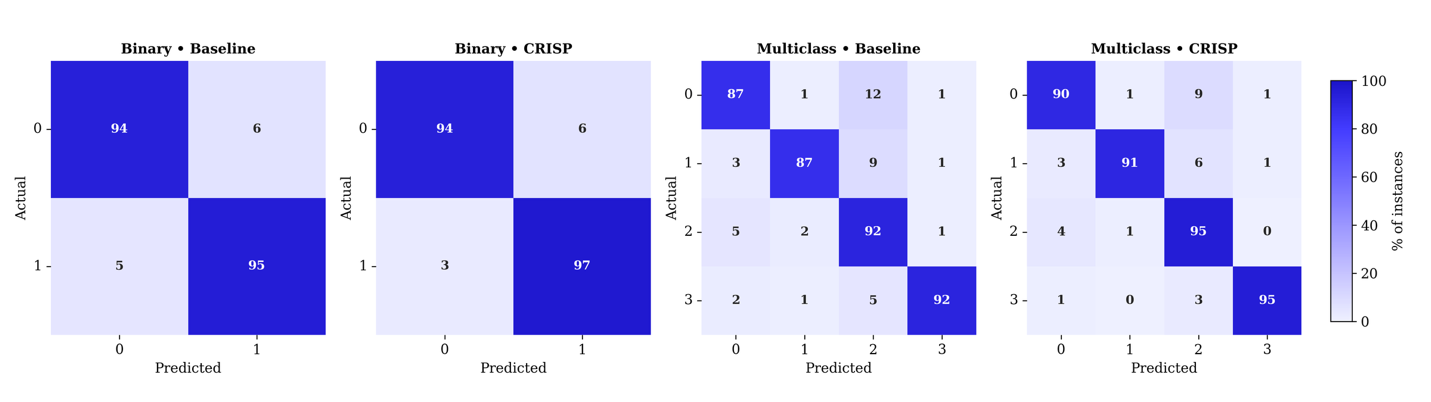


Figure S6. Confusion matrices (percentage view) for the Random Forest (RF) model under the overall evaluation protocol. Panels are ordered left to right: (1) Binary baseline, (2) Binary with CRISP, (3) Multiclass baseline, (4) Multiclass with CRISP. Hoehn and Yahr grades 0 to 3 are used for severity levels. CRISP reduces binary false-negatives from 5% to 3% and leads to cleaner separation between adjacent multiclass grades, particularly grades 0 to 1 and 1 to 2. Axes denote true (rows) and predicted (columns) labels.


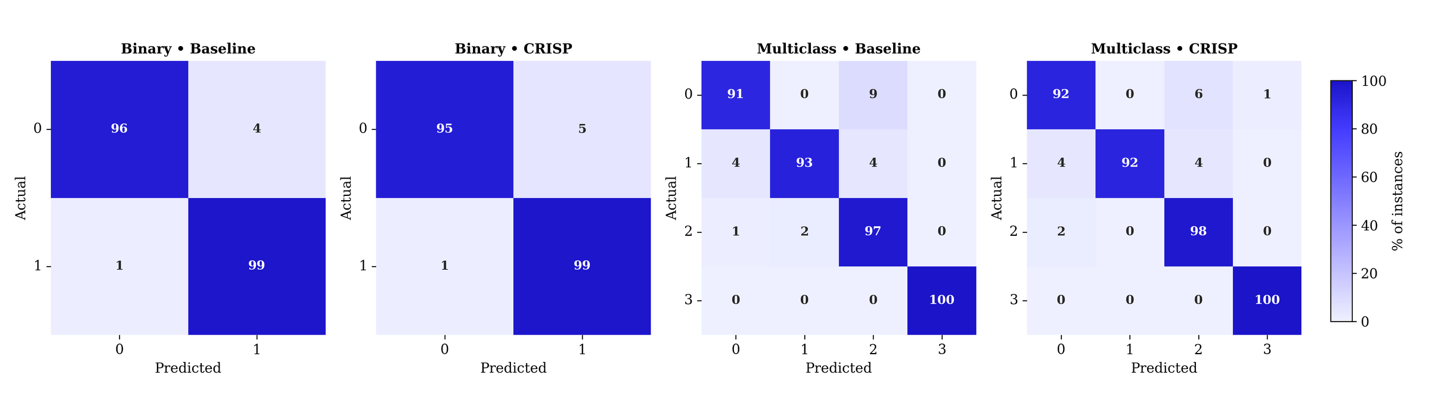


Figure S7. Confusion matrices (percentage view) for the Random Forest (RF) model under the subject-wise evaluation protocol. Panels are ordered left to right: (1) Binary baseline, (2) Binary with CRISP, (3) Multiclass baseline, (4) Multiclass with CRISP. Hoehn and Yahr grades 0 to 3 are used for severity levels. CRISP maintains strong binary accuracy while slightly reducing false-negatives. In the multiclass setting, it improves predictions for grades 0 and 2 and preserves perfect accuracy for grade 3. Axes indicate true (rows) and predicted (columns) labels.


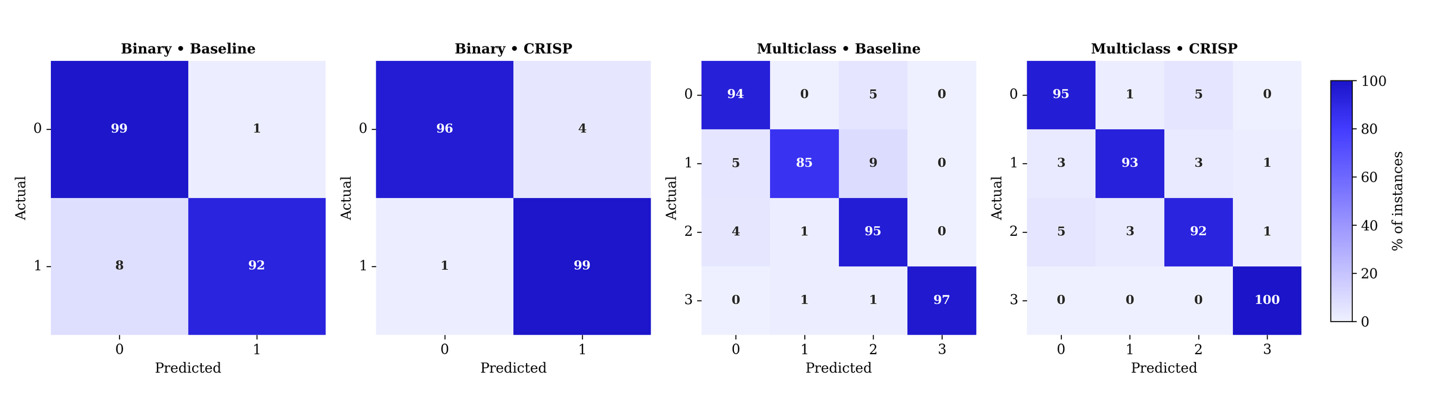


Figure S8. Confusion matrices (percentage view) for the Gradient Boosting (GB) model under the overall evaluation protocol. Panels are ordered left → right: (1) Binary baseline, (2) Binary with CRISP, (3) Multiclass baseline, (4) Multiclass with CRISP (Hoehn–Yahr grades 0–3). CRISP reduces binary false-negatives from 8% to 1%, while multiclass misclassifications between adjacent grades are also lowered particularly for grades 1 and 2. Axes denote true labels (rows) and predicted labels (columns).


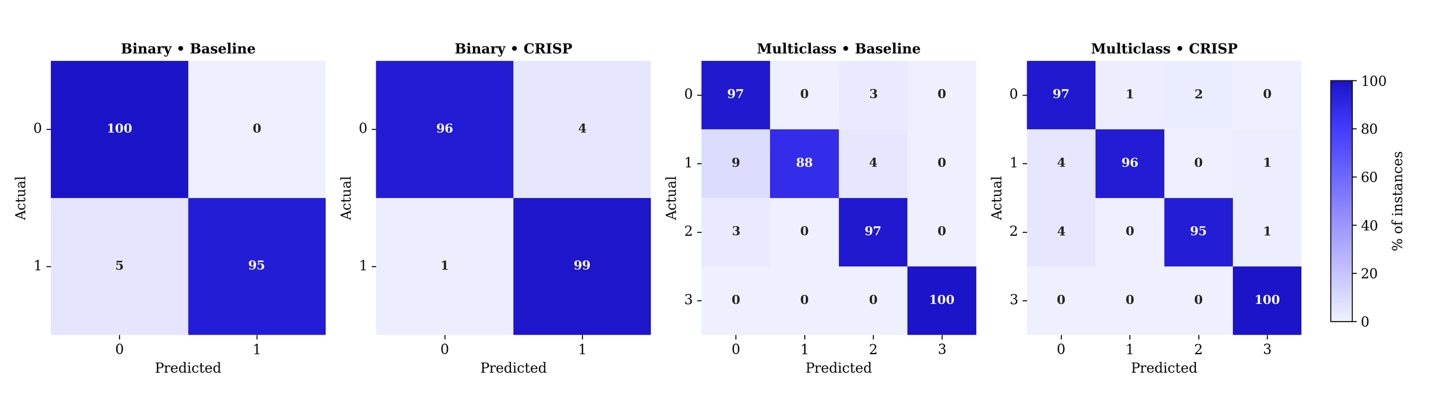


Figure S9. Confusion matrices (percentage view) for the Gradient Boosting (GB) model under the subject-wise evaluation protocol. Panels are ordered left → right: (1) Binary baseline, (2) Binary with CRISP, (3) Multiclass baseline, (4) Multiclass with CRISP (Hoehn–Yahr grades 0–3). CRISP lowers binary false-negatives from 5% to 1% and tightens multiclass predictions particularly for grades 1 and 2. Axes denote true (rows) and predicted (columns) labels.


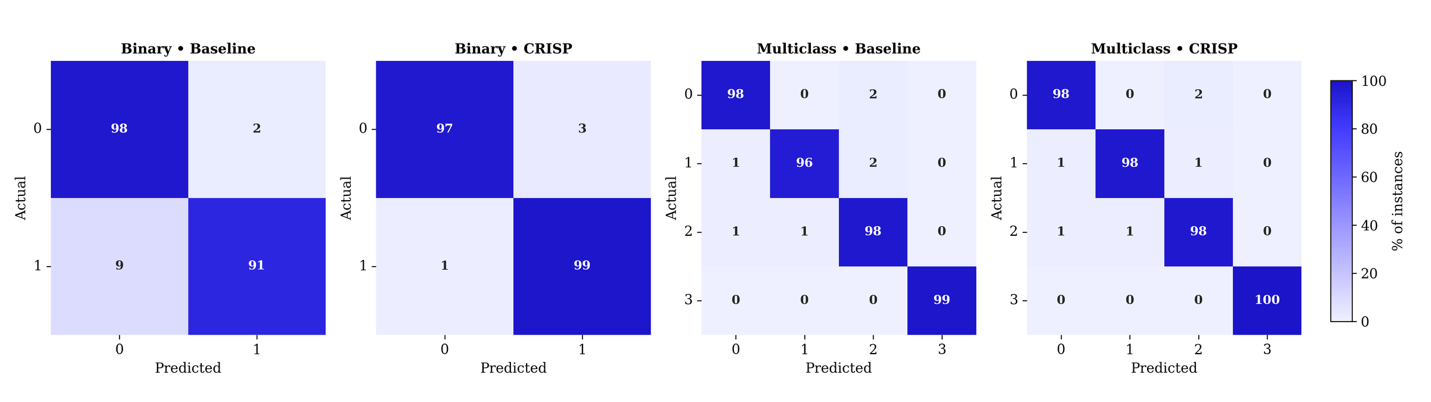


Figure S10. Confusion matrices (percentage view) for the XGBoost (XGB) model under the overall evaluation protocol. Panels are ordered left to right: (1) Binary baseline, (2) Binary with CRISP, (3) Multiclass baseline, (4) Multiclass with CRISP. Hoehn and Yahr grades 0 to 3 are used for severity levels. CRISP reduces binary false-negatives from 9% to 1%, and further sharpens multiclass separation, especially between adjacent grades such as 1 and 2. Grade 3 accuracy improves from 99% to 100%. Axes indicate true (rows) and predicted (columns) classes.


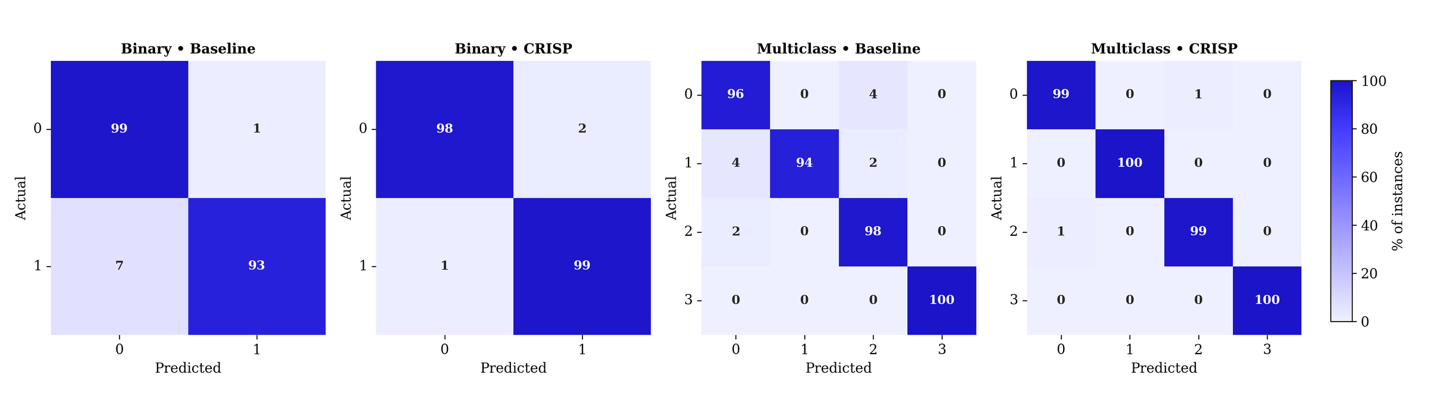


Figure S11. Confusion matrices (percentage view) for the XGBoost (XGB) model under the subject-wise evaluation protocol. Panels are ordered left to right: (1) Binary baseline, (2) Binary with CRISP, (3) Multiclass baseline, (4) Multiclass with CRISP. Hoehn and Yahr grades 0 to 3 are used for severity levels. CRISP reduces binary false-negatives from 7% to 1%, and yields near-perfect multiclass accuracy across all grades, with grades 1 and 3 reaching 100%. Axes indicate true (rows) and predicted (columns) labels.

**S4. Radar Plot Visualizations**


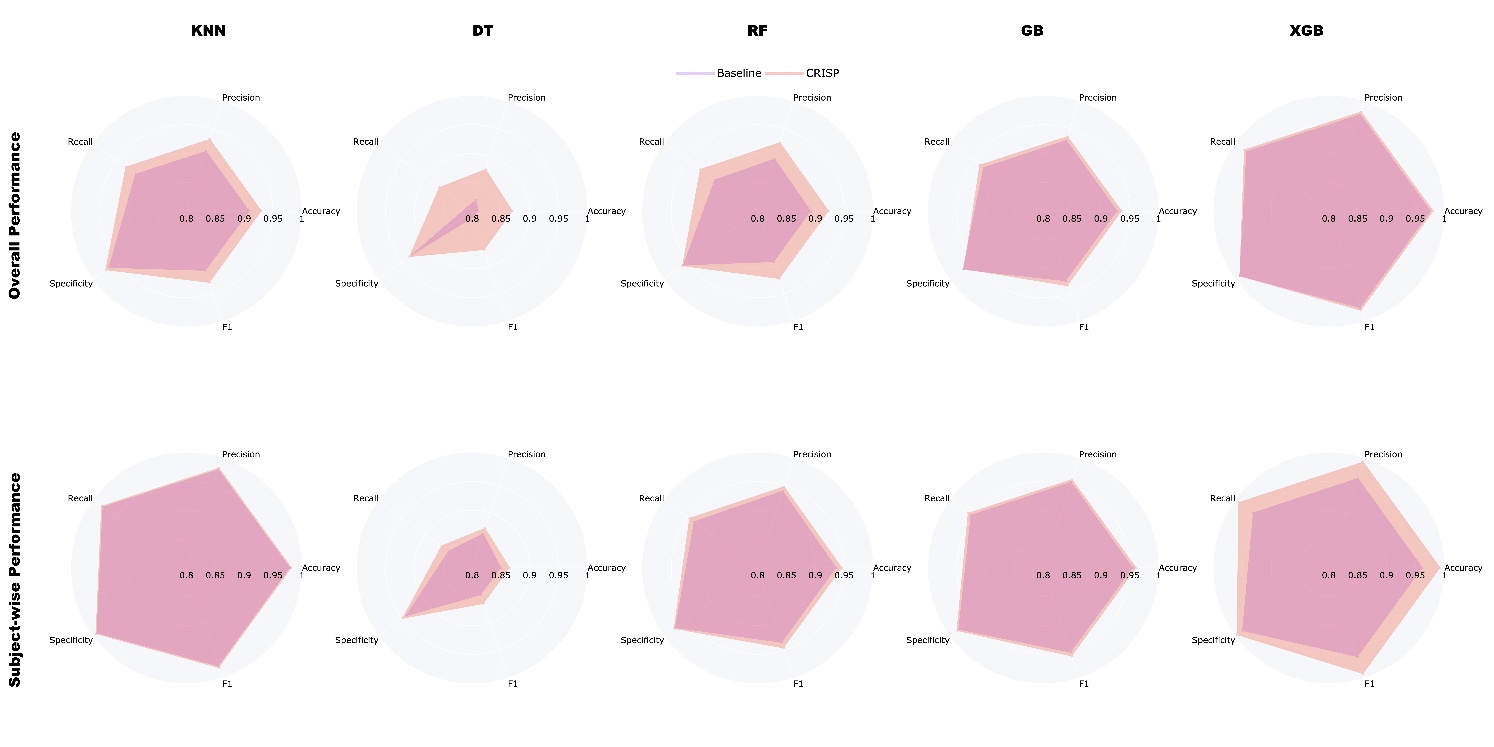


Figure S1. Radar-chart comparison of baseline models (violet) versus the proposed CRISP pipeline (orange-red) across five macro-averaged metrics, Accuracy, Precision, Recall, Specificity and F1-score, for multiclass Parkinson’s-disease severity grading (Hoehn & Yahr scale). The top row displays Overall Performance; the bottom row shows per-patient Subject-wise Performance. Each of the five columns corresponds to one classifier (KNN, DT, RF, GB, XGB). Across all models the CRISP polygons expand beyond the baseline shapes, indicating consistent improvements on every metric, with the most pronounced gains observed for Gradient Boosting and XGBoost.

**S5. Pre Processing**


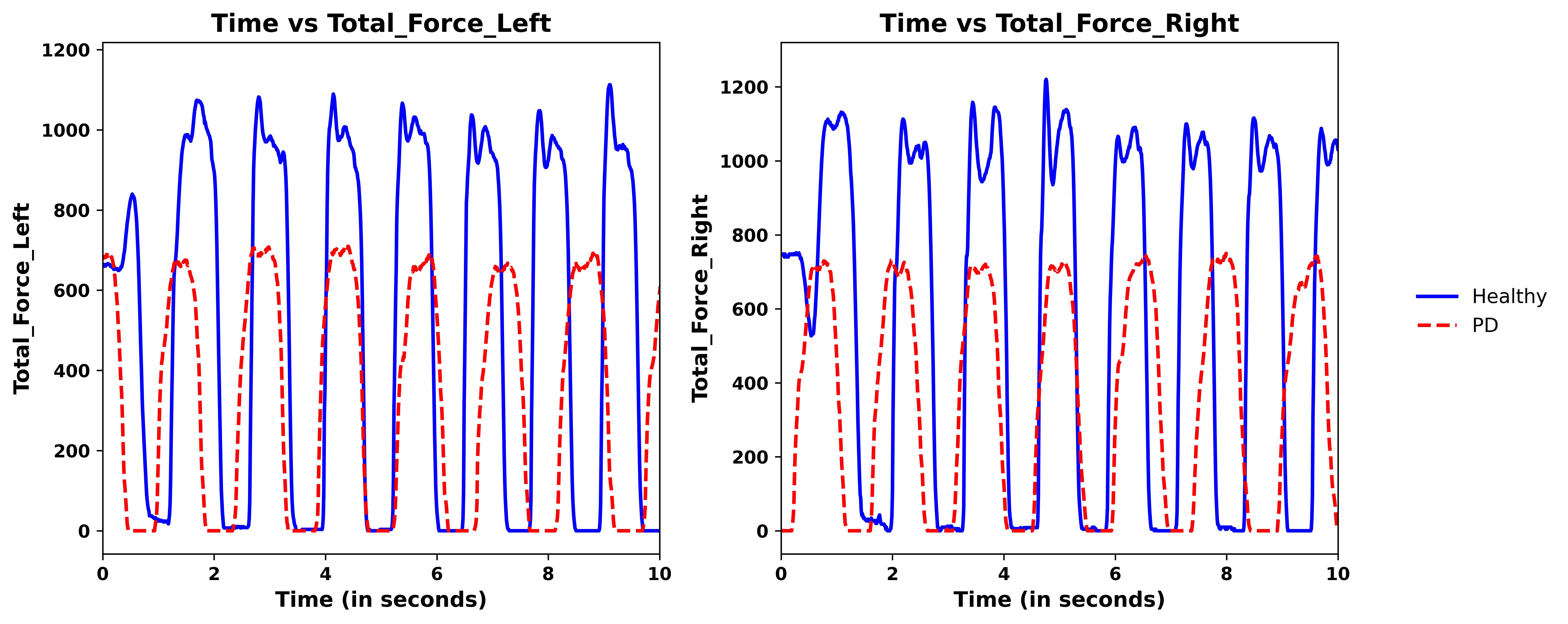


*Figure S12. Comparison of VGRF signals in healthy vs. PD subjects.* VGRF trajectories show lower peak force and irregular step timing in the PD subject, highlighting gait abnormalities.


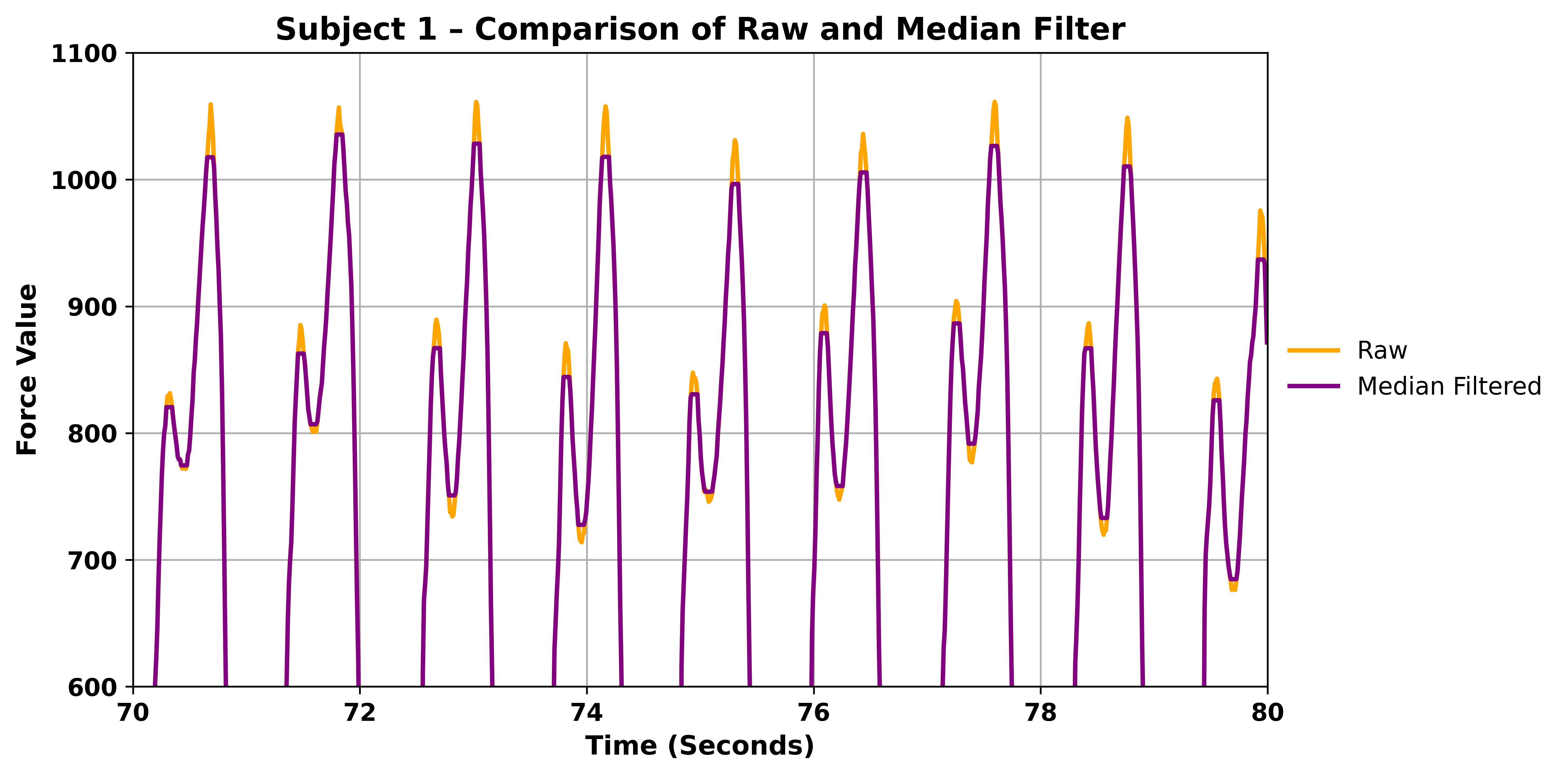


*Figure S13. Enhanced signal quality through median filtering.* Raw total-force signal (orange) from the left foot of a representative trial is superimposed with the signal post 10-point median filtering (magenta). The filtering removes high-frequency artifacts while preserving gait cycle integrity, enabling robust gait-event detection.

**S6. Cohort Demographics**

The final dataset comprised vertical ground-reaction force (VGRF) recordings from 169 participants, including 93 individuals with PD and 76 age- and sex-matched healthy controls. PD cases were evenly distributed across the three datasets Ga, Si, and Ju while healthy controls were slightly more prevalent in the Si and Ju cohorts.

Table S13 summarizes the demographic and clinical characteristics of all subjects. Among PD participants, the average age ranged from 61.6 to 67.2 years across datasets, compared to 57.9 to 64.5 years in the healthy cohort. Notably, Dataset Ju had a younger control group (mean age = 39.3 ± 18.5 years) potentially due to broader recruitment criteria. Height and weight distributions were similar across groups, although PD participants in Ju were slightly taller on average. Gender distribution was balanced overall, with 59 males and 34 females among PD subjects, and 40 males and 32 females among controls. Disease severity was graded using the Hoehn and Yahr (HY) scale: the majority of PD cases fell into stages 2 or 2.5, with a smaller number diagnosed at stage 3, particularly in the Ju cohort. These balanced demographics ensure that subsequent classification analyses are not confounded by age, gender, or dataset-specific biases.

The dataset comprises of demographic and clinical details including age, sex, height, weight, and Hoehn–Yahr scores. It covers three studies (Ga, Si, Ju) with differing demographics, protocols, and gait conditions (e.g., dual-task walking, treadmill, rhythmic stimulation). As shown in Table 1, these subgroups vary in age, sex, height, weight, and severity levels (2–3), capturing intra- and inter-subject diversity that supports generalizability. Stage 0 denotes healthy individuals with no clinical signs of PD. We report demographic breakdowns including age, gender, and Hoehn–Yahr staging to contextualize cohort composition. Each sub-dataset applied strict inclusion criteria (Hoehn–Yahr stage 2–3, no motor fluctuations, MMSE screening) and excluded neurological, cardiovascular, or musculoskeletal comorbidities. Clinical assessments included UPDRS, TUG, Berg Balance Scale, and computerized cognitive tests where applicable. All studies had ethical approval and followed the Declaration of Helsinki. This dataset provides substantial clinical and demographic diversity, supporting gait-based biomarker research in PD.

Table S 13. Summary of demographic and clinical statistics across three datasets (Ga, Si, Ju), including subject counts, gender distribution, age, height, weight (mean ± SD), and Hoehn & Yahr severity levels. This highlights the diversity of the cohort used in our evaluation.

| Variable | Dataset Ga | Dataset Si | Dataset Ju |
| --- | --- | --- | --- |
| Total PD | 29 | 35 | 29 |
| Total Healthy | 18 | 29 | 26 |
| Female PD | 9 | 13 | 13 |
| Male PD | 20 | 22 | 16 |
| Female Healthy | 8 | 11 | 14 |
| Male Healthy | 10 | 18 | 12 |
| PD Age (Mean ± SD) | 61.6 ± 8.8 | 67.2 ± 9.1 | 66.8 ± 10.8 |
| Healthy Age (Mean ± SD) | 57.9 ± 6.7 | 64.5 ± 6.8 | 39.31 ± 18.5 |
| PD Height (m) | 1.67 ± 0.07 | 1.66 ± 0.07 | 1.87 ± 0.15 |
| Healthy Height (m) | 1.68 ± 0.08 | 1.69 ± 0.07 | 1.83 ± 0.08 |
| PD Weight (kg) | 73.1 ± 11.2 | 70.3 ± 8.4 | 75.1 ± 11.0 |
| Healthy Weight (kg) | 74.2 ± 12.7 | 71.5 ± 11.0 | 66.8 ± 11.07 |
| PD Severity 2 | 15 | 29 | 12 |
| PD Severity 2.5 | 8 | 6 | 13 |
| PD Severity 3 | 6 | 4 | 0 |

**S7**. **Spatiotemporal Features**

Table S14. Definitions of the eight spatiotemporal gait features extracted from each cycle.

| Gait Parameters | Spatiotemporal Features | Definition |
| --- | --- | --- |
| 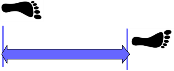 | Step Time Calculation | Time interval between the heel strike of one foot and the heel strike of the opposite foot. |
| 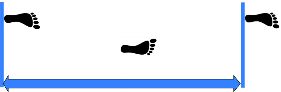 | Stride Time Calculation | Time taken for a full cycle of one foot, from one heel strike to the next heel strike of the same foot. |
| 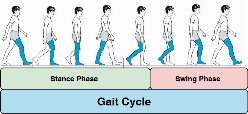 | Stance and Swing Time | Stance Time: Duration the foot is on the ground during a stride. Swing Time: Duration the foot is in the air. |
| 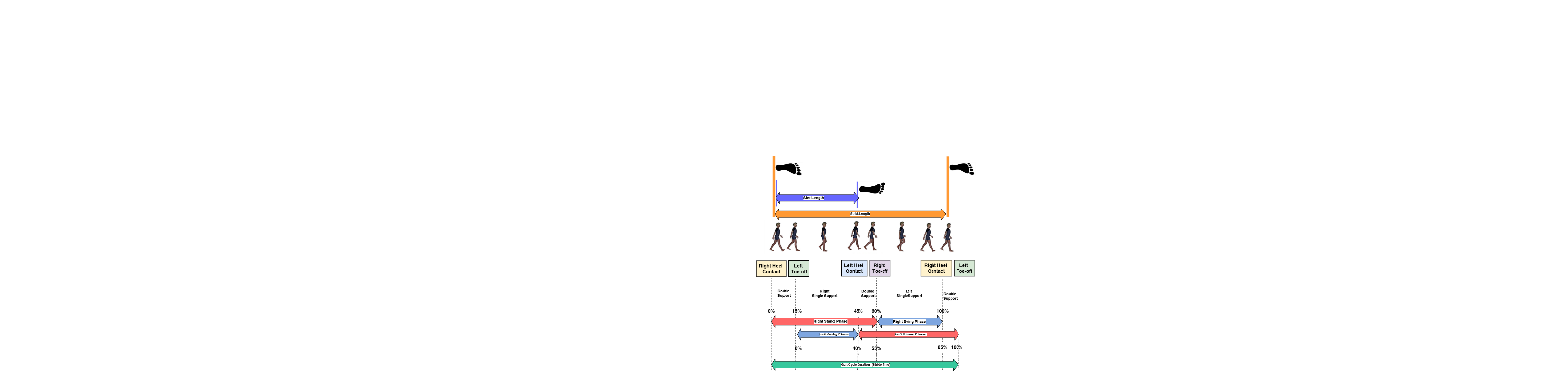 | % Stance & Swing Time | Calculated as a percentage of the total Stride Time. |
| 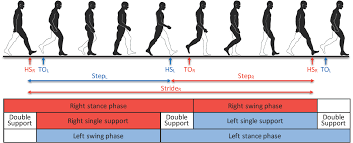 | Cadence | Number of steps taken per minute. |
| 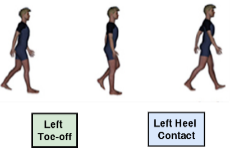 | SS ratio | SS Ratio: Proportion of a stride that one foot is in swing phase while the other foot is in stance. |
| 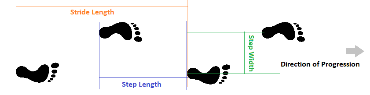 | Stride Length | Stride length is the distance covered in one complete cycle of gait |
| 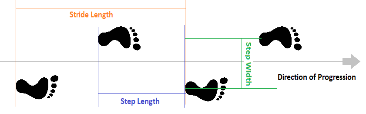 | Step length | Step length is the distance measured from the heel strike of one foot to the heel strike of the opposite foot |

Table S15*. Mean ± SD values of the ten principal gait parameters for healthy controls and Parkinson disease participants.*

| Gait Cycle parameters | Mean± SD (HC) | | Mean± SD (PD) | |
| --- | --- | --- | --- | --- |
| *Spatiotemporal Features* | *Left* | *Right* | *Left* | *Right* |
| Stride Time | 1.09± 0.08 | 1.10±0.09 | 1.10±0.12 | 1.10±0.11 |
| Stance Time Left | 0.63±0.05 | 0.63±0.06 | 0.65±0.09 | 0.65±0.08 |
| Swing Time | 0.46±0.03 | 0.46±0.03 | 0.44±0.04 | 0.44±0.04 |
| Stance % | 57.96±1.52 | 57.77±1.42 | 59.39±2.47 | 59.28±1.96 |
| Swing % | 42.03±1.52 | 42.22±1.42 | 40.60±2.47 | 40.71±1.96 |
| SS Ratio | 0.42±0.01 | 0.42±0.01 | 0.40±0.02 | 0.40±0.01 |
| Cadence | 96.24±25.57 | 96.24±25.57 | 94.19±32.80 | 94.19±32.80 |
| Step Time | 1.02±0.58 | 1.02±0.58 | 1.13±0.74 | 1.13±0.74 |
| Step length | 1.26±0.72 | 1.26±0.72 | 1.16±0.80 | 1.16±0.80 |
| Stride length | 2.52±1.44 | 2.52±1.44 | 2.32±1.61 | 2.32±1.61 |





Figure S14. This illustration summarizes key gait metrics: (a) spatial parameters: step length and stride length; (b) temporal markers: heel-strike and toe-off timings for each foot; (c) gait phases: stance and swing durations, visualized both in absolute and relative terms. These metrics underpin our feature set for PD detection.
